# Supplementary material for: Prospective study to explore changes in quality of care and perinatal outcomes after implementation of perinatal death audit in Uganda
Source: BMJ Open. 2020 Jul 8;10(7):e027504. doi: 10.1136/bmjopen-2018-027504 (PMC7348647; doi:10.1136/bmjopen-2018-027504)
Supplement: Supplementary data [file bmjopen-2018-027504supp001.pdf]

Supplementary Materials

Supplementary Table 1: Stillbirth rate (per 1000 births), early neonatal mortality rate (per 1000 live births) and perinatal mortality rate (per 1000 births).

Year	Total Births	Live Births	Stillbirth	Stillbirth Rate	Early NND	Early NND Rate	Perinatal Mortality	Perinatal Mortality Rate
2006	6468	6020	213	32.9	96	16	309	47.8
2007	7290	6881	210	28.8	193	28.1	403	55.3
2008	8386	8408	291	34.7	145	17.3	436	52.0
2009	8452	7995	207	24.5	114	14.3	321	38.0
2010	9513	8877	229	24.1	132	14.9	362	38.0
2011	8050	7610	220	27.3	166	21.8	386	47.9
2012	6732	6593	149	22.1	167	25.4	316	47.0
2013	6376	6011	183	28.7	117	19.4	300	47.0
2014	6026	5764	136	22.6	110	19	246	40.8
2015	5462	5222	120	22	112	21.5	232	42.6

Supplementary Table 2: **Cause of death for stillbirth and early neonatal death.**

**Cause of Death**	**Macerated Stillbirth n=142 (%)**	**Fresh Stillbirth n=124 (%)**	**Early Neonatal Death n=259 (%)**	**TOTAL n=526 (%)**
Respiratory distress syndrome	-	-	47 (18.0)	**47 (8.9)**
Infections / Septicaemia	1 (0.7)	0	6 (2.3)	**7 (1.3)**
Congenital anomaly	0	4 (3.2)	9 (3.5)	**13 (2.5)**
Meningitis	-	-	1 (0.4)	**1 (0.2)**
Meconium aspiration pneumonia	-	-	40 (15.4)	**40 (7.6)**
Intrapartum-related hypoxia	16 (11.3)	59 (47.6)	111 (42.9)	**186 (35.4)**
Haemorrhagic disease of newborn	-	-	5 (1.9)	**5 (1.0)**
Unknown	125 (88.0)	61 (49.6)	40 (15.4)	**227 (43.2)**

Supplementary Table 3: Case fatality rates (CFR)

Cause of death		2006	2007	2008	2009	2010	2011	2012	2013	2014	2015
**Intrapartum-related hypoxia**	Intrapartum-related hypoxia admissions	202	291	334	302	316	297	247	252	195	187
	Intrapartum-related hypoxia deaths	43	55	47	46	40	51	34	30	19	29
	**Intrapartum-related hypoxia CFR (%)**	**21.3**	**18.9**	**14.1**	**15.2**	**12.7**	**17.2**	**13.8**	**11.9**	**9.7**	**15.5**
**Prematurity**	Prematurity admissions	163	168	194	246	210	328	286	304	336	371
	Premature deaths	43	35	43	36	49	53	60	54	51	41
	**Prematurity CFR (%)**	**26.4**	**20.8**	**22.2**	**14.6**	**23.3**	**16.2**	**21.0**	**17.8**	**15.2**	**11.1**
**Infections**	Number of admissions for infections	104	149	82	128	120	140	129	171	141	87
	Deaths due to infections	2	0	0	4	3	4	4	0	2	5
	**Infections CFR (%)**	**1.9**	**0**	**0**	**3.1**	**2.5**	**2.9**	**3.1**	**0.0**	**1.4**	**5.7**

Supplementary Table 4: Standard of care

**Standard of care**	**MSB (%)**	**FSB (%)**	**ENND (%)**	**Total (%)**
Optimal	82 (67.8)	58 (49.2)	112 (45.7)	262 (53.0)
Probably acceptable	21 (17.4)	28 (23.7)	58 (23.7)	107 (21.7)
Probably sub-optimal	12 (9.9)	24 (20.3)	46 (18.8)	82 (16.6)
Sub-optimal	6 (5.0)	8 (6.8)	29 (11.8)	43 (8.7)
**Total**	**121 (100)**	**118 (100)**	**245 (100)**	**494 (100)**
