# Supplementary material for: Prospective study to explore changes in quality of care and perinatal outcomes after implementation of perinatal death audit in Uganda
Source: BMJ Open. 2020 Jul 8;10(7):e027504. doi: 10.1136/bmjopen-2018-027504 (PMC7348647; doi:10.1136/bmjopen-2018-027504)
Supplement: Author's manuscript [file bmjopen-2018-027504.draft_revisions.pdf]

BMJ Open is committed to open peer review. As part of this commitment we make the peer review history of every article we publish publicly available.

When an article is published we post the peer reviewers' comments and the authors' responses online. We also post the versions of the paper that were used during peer review. These are the versions that the peer review comments apply to.

The versions of the paper that follow are the versions that were submitted during the peer review process. They are not the versions of record or the final published versions. They should not be cited or distributed as the published version of this manuscript.

BMJ Open is an open access journal and the full, final, typeset and author-corrected version of record of the manuscript is available on our site with no access controls, subscription charges or pay-per-view fees (http://bmjopen.bmj.com).

If you have any questions on BMJ Open's open peer review process please email info.bmjopen@bmj.com

BMJ Open

Prospective Study to Explore Changes in Quality of Care and Perinatal Outcomes After Implementation of Perinatal Death Audit in Uganda

Journal:	*BMJ Open*
Manuscript ID	bmjopen-2018-027504
Article Type:	Research
Date Submitted by the Author:	26-Oct-2018

Complete List of Authors:	Kirabira, Victoria; Nsambya Hospital<br>Aminu, Mamuda; Liverpool School of Tropical Medicine, Centre for Maternal and Newborn Health ; Liverpool School of Tropical Medicine<br>Dewez, Juan; Liverpool School of Tropical Medicine, Centre for Maternal and Newborn Health<br>Byaruhanga, Romano; Nsambya Hospital<br>Okong, Pius; Nsambya Hospital<br>van den Broek, Nynke; Liverpool School of Tropical Medicine, Centre for Maternal and Newborn Health

Keywords:	OBSTETRICS, AUDIT, Reproductive medicine < GYNAECOLOGY, Clinical audit < HEALTH SERVICES ADMINISTRATION & MANAGEMENT, Quality in health care < HEALTH SERVICES ADMINISTRATION & MANAGEMENT

SCHOLARONE™
Manuscripts

Prospective Study to Explore Changes in Quality of Care and Perinatal Outcomes After Implementation of Perinatal Death Audit in Uganda

----

Victoria Nakibuuka Kirabira[a], Mamuda Aminu[b§], Juan Dewez[b], Romano Byaruhanga[a], Pius Okong[a], Nynke van den Broek[b]

[a] Nsambya Hospital, P.O. Box 7146, Kampala, Uganda

[b] Centre for Maternal and Newborn Health, Liverpool School of Tropical Medicine, Pembroke Place, Liverpool L3 5QA, UK

[§]Corresponding Author Contact Details:

Dr Mamuda Aminu
Centre for Maternal and Newborn Health,
Liverpool School of Tropical Medicine, Pembroke Place, Liverpool L3 5QA.
Tel: +44(0)151 705 3342
Email: Mamuda.Aminu@lstmed.ac.uk

Author email addresses:

VN Kirabira	nakibuukarv@yahoo.com
JE Dewez	manueldewez@hotmail.com
R Byaruhanga	byaruhangarn@yahoo.com
P Okong	puisokong@yahoo.com
NR van den Broek	Nynke.Broek@lstmed.ac.uk

Running Title: Perinatal death audit in Uganda

Keywords: perinatal death, cause of perinatal death, quality improvement

***

BMJ Global Health (5,000 words total; 300 words in structured abstract; 100 refs; 5 tables/figs)
Word Count (Body Only): 3680

Abstract: 300

For peer review only - http://bmjopen.bmj.com/site/about/guidelines.xhtml

For peer review only

ABSTRACT

Objectives: To describe changes in perinatal outcomes observed after the introduction of perinatal death audit in a tertiary hospital in Kampala.

Setting: Nsambya Hospital, Kampala, is a tertiary hospital receiving 7,000 deliveries per year and provides 24-hour comprehensive emergency obstetric and newborn care services.

Participants: Perinatal deaths from 2006 to 2015.

Primary outcome: Change in perinatal outcomes (stillbirths and neonatal deaths)

Secondary outcome: Change in case fatality rates for selected causes of perinatal mortality.

Results: A total of 526 perinatal deaths were included: 142 (27.0%) fresh stillbirths, 125 (23.8%) macerated stillbirths and 259 (49.2%) early neonatal deaths. The perinatal mortality rate decreased from 48.4/1000 births in 2006 (95% CI: 35.5 – 62.6) to 43/1000 births in 2015 (95% CI: 36.9 – 48.9). Stillbirth rate decreased from 32.9/1000 births (95% CI: 25.0 – 41.5) to 22/1000 births (95% CI: 17.3 – 26.6). However, the early neonatal mortality rate increased from 16 (95% CI: 7.8 – 25.0) to 21 (95% CI: 15.9 – 26.8) per 1000 live births.

Intrapartum-related hypoxia was the leading cause of perinatal mortality (35.4% of all cases), followed by respiratory distress syndrome (8.9%) and meconium aspiration pneumonia (7.6%). Cause of death was unknown in 43.2% of cases. The proportion of deaths with unknown cause decreased from 34.1% (overall) in 2008 to 2.7% in 2013.
Case fatality rates decreased between 2006 and 2015 for intrapartum-related hypoxia (21.3% to 15.5%) and for complications of prematurity (26.4% to 11.1%). However, case fatality rates for infection increased from 1.9% (2006) to 5.7% (2015).

Conclusion: Perinatal death audit helped to identify priority areas for which action was taken to improve the quality of care and resulting outcomes. Healthcare providers should be encouraged to conduct reviews and improve clinical documentation to help reduce proportion of deaths with unknown cause.

For peer review only - http://bmjopen.bmj.com/site/about/guidelines.xhtml

ARTICLE SUMMARY

- Following the implementation of perinatal death audit, perinatal mortality rate decreased from 48.4/1000 births in 2006 (95% CI: 35.5 – 62.6) to 43/1000 births in 2015 (95% CI: 36.9 – 48.9); stillbirth rate decreased from 32.9/1000 births (95% CI: 25.0 – 41.5) to 22/1000 births (95% CI: 17.3 – 26.6). However, the early neonatal mortality rate increased from 16 (95% CI: 7.8 – 25.0) to 21 (95% CI: 15.9 – 26.8) per 1000 live births.
- Perinatal death audit helped to identify priority areas for which action was taken to improve the quality of care and resulting outcomes.

Strengths and limitations of this study

- This study has demonstrated how the introduction of perinatal death audit helped to identify the main causes of death and contributing factors and priorities action.
- While it is difficult to attribute all changes in perinatal outcomes to perinatal death audit alone, the process has helped to identify priority areas for improvement in care.
- Few studies explored the use of perinatal death audit to improve care in low-resource settings.

For peer review only

For peer review only - http://bmjopen.bmj.com/site/about/guidelines.xhtml

BACKGROUND

Currently, about 2.7 million neonatal deaths and 2.6 million stillbirths occur worldwide every year. Of these, the majority occur in low- and middle-income countries, and more than half (55%) of the global stillbirths occur in sub-Saharan Africa and South Asia [1]. Uganda's perinatal mortality is estimated to be 36 deaths per 1000 births and remains unacceptably high. Almost 50% of these are fresh stillbirths [2]. The neonatal mortality rate (21 per 1000 live births) in Uganda is also high, contributing 50% of the infant mortality rate [3]. It is crucial that interventions are put in place to reduce preventable neonatal deaths and stillbirths.

Perinatal death audit (or review) can be used to identify areas of care which can be strengthened to improve perinatal outcomes [4]. It involves reviewing cases of mortality with a view to identifying avoidable factors and implementing changes to prevent future deaths. A population-based cohort study of 943 perinatal mortalities that occurred in 90 hospitals in The Netherlands reported a decrease in substandard care (from 10% to 5%) and perinatal mortality (from 2.3 to 2.0/1000 births; $p<0.00001$) after the introduction of perinatal death audit [5].

Similarly, in a systematic review focusing on low- and middle-income countries, a meta-analysis of 7 before-and-after studies reported up to 30% (95% confidence interval, 21%–38%) reduction in perinatal mortality after introduction of perinatal death audit in healthcare facilities [6]. There is, however, a paucity of data from low-resources settings on the use of and effectiveness of perinatal death audit to improve quality of care provided to mothers and their babies. The systematic review recommended more research in key areas, including exploring the effect of facility-based perinatal death audit on changing the quality of care [6].

This paper describes changes in perinatal outcomes observed after introduction of perinatal death audit in Kampala and explores the trends in case fatality rates for the major identified causes of perinatal mortality.

For peer review only - http://bmjopen.bmj.com/site/about/guidelines.xhtml

METHODS

Study setting

This is a descriptive analysis of data before and after the introduction of perinatal death audit at Nsambya Hospital, a tertiary hospital in Kampala, Uganda, which serves as the referral centre for Kampala and Makindye Health Sub-districts. The facility receives 7,000 deliveries per year and provides 24-hour comprehensive emergency obstetric and newborn care services, with a staff complement of 6 obstetricians, 18 resident doctors, 5 intern doctors and 40 midwives.

The newborn care unit admits 2,500 babies annually and has a paediatrician, two residents and up to four midwives on duty per shift. It has 40 beds, including 10 high care area beds, and provides essential newborn care including; oxygen therapy and low-cost bubble continuous positive airway pressure (CPAP), surfactant administration, kangaroo mother care and phototherapy.

Data collection

Perinatal death audit using locally developed tools and guidelines started in 2008 and was led by a multidisciplinary audit team comprising of obstetricians, paediatricians, resident doctors, midwives and administrators. Team meetings were held weekly, chaired by the most senior obstetrician or paediatrician. Perinatal death was defined as "death of a fetus greater than a birth weight of 1000g if measured, or a gestational age of at least 28 completed weeks or more, or a body length (crown-heel) of at least 35cm or more"[7] and included all stillbirths and deaths within the first week of life (early neonatal deaths). The number of perinatal deaths was documented daily using clinical handover reports and registers in the labour ward and neonatal special care unit. One-quarter of the cases (every 4th case) were selected for audit from each of the following categories: fresh stillbirths, macerated stillbirths and early neonatal deaths.

The number of admissions in the newborn unit, reasons for admission, death and case fatalities rates for intrapartum-related hypoxia, infection and prematurity were collected for the period before the perinatal audit was introduced (2006 – 2007) and subsequently for the period after commencement of the perinatal death audit (2008 – 2015). The data was obtained and aggregated monthly through a retrospective analysis of registers and patient records.

For peer review only - http://bmjopen.bmj.com/site/about/guidelines.xhtml

The audit process

For each case selected for review, an audit form was completed by a resident doctor, who also provided a narrative summary for each case. Sources of information included: clinical handover books, maternity registers, newborn admission register, and clinical notes for mothers and newborn babies. Each case was then presented to the rest of the audit team for detailed discussion. Cause of death was identified by consensus, and care provided was judged against existing local protocols and national guidelines. No clinical autopsies were performed due to lack of a full-time pathologist at the hospital.

The quality of care provided was also graded by consensus and classified into four categories: optimal, probably acceptable, probably suboptimal and suboptimal care. The overall grading of the care provided to the mother and baby, the identified specific areas of suboptimal care and the recommendations for improvement were documented.

The summary of each case included a list of the actions to be taken. In each case, a specific person was appointed to lead the implementation of the relevant actions. During monthly meetings, implementation progress was compared with the action plan by the wider audit team. A number of interventions were thus introduced gradually over the years to address the areas of suboptimal care.

Data management and analysis

For quantitative data, SPSS software (IBM, version 22) was used to conduct standard descriptive analyses to determine proportions. Stillbirth and perinatal mortality rates were expressed per 1000 total births, while early neonatal mortality rate was expressed per 1000 live births. Case fatality rate (CFR) was defined as the proportion of deaths among cases diagnosed with the disease. Statistical significance was calculated at 95% confidence interval or p-value of ≤ 0.05.

Since low birth weight and prematurity are both associated with a higher risk of perinatal mortality, a one-way between-groups analysis of variance (ANOVA) was conducted to explore changes in birthweight and gestational age over time.

A narrative summary description was used to report actions agreed and implemented following an audit. These were documented chronologically as derived from minutes of the audit meetings.

For peer review only - http://bmjopen.bmj.com/site/about/guidelines.xhtml

Patient and public involvement

Approval for the study was obtained from the Nsambya Hospital Ethical Review Committee. Individual patient consent was not applicable as the research team extracted anonymized data from case notes.

RESULTS

Demographic and clinical characteristics:

A total of 58,997 births and 2,616 perinatal deaths occurred between 2008 and 2015. Of these, 603 (23%) perinatal deaths were selected for audit. After review, 526 were found to meet the criteria for perinatal mortality: 142 (27.0%) fresh stillbirths, 125 (23.8%) macerated stillbirths and 259 (49.2%) early neonatal deaths.

The majority of cases came to the hospital directly, with 17% referrals (Table 1). Mothers had a mean age of 27.3 years (SD: 5.1); median parity of 2 (SD of 1.6). Just over one-third of fresh stillbirths and early neonatal deaths were delivered by Caesarean section. The mean birth weight of babies was 2610 grams (SD: 969.4). Between 23.2% and 39.4% were considered to have been born prematurely.

Table 1: Demographic and clinical characteristics

Characteristics	Fresh stillbirth n=125 (%)	Macerated stillbirth n=142 (%)	Early neonatal death n=259 (%)	Total n=526 (%)
**Referral**				
From other healthcare facility	32 (25.6)	15 (10.6)	37 (14.3)	84 (16.0)
From Traditional Birth Attendant	1 (0.8)	1 (0.7)	1 (0.4)	3 (0.6)
**Parity**				
1	35 (28.0)	48 (33.8)	129 (49.8)	212 (40.3)
2 to 4	64 (51.2)	67 (47.2)	84 (32.4)	215 (40.9)
5 or more	10 (8.0)	15 (10.6)	18 (6.9)	43 (8.2)
**Antenatal care**				
At least 1 ANC visit	107 (85.6)	129 (90.8)	234 (90.3)	470 (89.4)
4 or more ANC visits	38 (30.4)	47 (33.1)	98 (37.8)	183 (34.8)
**Type of pregnancy**				
Single pregnancy	113 (90.4)	131 (92.3)	227 (87.6)	471 (89.5)
Multiple pregnancy	2 (1.6)	5 (3.5)	14 (5.4)	21 (4.0)

For peer review only - http://bmjopen.bmj.com/site/about/guidelines.xhtml

**Mode of delivery**				
Spontaneous vaginal delivery	67 (53.6)	123 (86.6)	143 (55.2)	333 (63.3)
Assisted breech delivery	6 (4.8)	2 (1.4)	10 (3.9)	18 (3.4)
Instrumental delivery	4 (3.2)	1 (0.7)	3 (1.2)	8 (1.5)
Caesarean section	45 (36.0)	10 (7.0)	92 (35.5)	147 (27.9)
**Gestational age at delivery**				
<37 weeks	29 (23.2)	56 (39.4)	74 (28.6)	159 (30.2)
37-41 weeks	60 (48.0)	57 (40.1)	132 (51.0)	249 (47.3)
>41 weeks	5 (4.0)	4 (2.8)	9 (3.5)	18 (3.4)
**Baby's sex**				
Male	68 (54.4)	80 (56.3)	146 (56.4)	294 (55.9)
Female	52 (41.6)	57 (40.1)	99 (38.2)	208 (39.5)
**Birthweight**				
<1000g	1 (0.8)	16 (11.3)	9 (3.5)	26 (4.9)
1000-2499g	30 (24.0)	52 (36.6)	81 (31.3)	163 (31.0)
2500-4000g	77 (61.6)	61 (43.0)	149 (57.5)	287 (54.6)
>4000g	3 (2.4)	7 (4.9)	6 (2.3)	16 (3.0)

Trend in perinatal mortality rate:

A downward trend was observed for stillbirth rate and perinatal mortality rate (PNMR). However, early neonatal mortality rate (ENMR) showed a slight increase, which declined between 2007 and 2009 only to rise again between 2010 and 2012, and progress has been slow since then (Figure 1 and Supplementary Table 1). Overall, PNMR showed a slowly decreasing trend from 48.4/1000 births in 2006 (95% CI: 35.5 – 62.6) to 43/1000 births in 2015 (95% CI: 36.9 – 48.9) and stillbirth rate from 32.9 (95% CI: 25.0 – 41.5) to 22 (95% CI: 17.3 – 26.6). However, the ENMR showed an increase from 16 (95% CI: 7.8 – 25.0) to 21 (95% CI: 15.9 – 26.8) per 1000 live births.

An ANOVA to explore changes in birthweight and gestational age over time showed no statistically significant change in birthweight over time ($p = 0.872$), and no change in gestational age at birth over time ($p = 0.219$).

Cause of death:

Overall, intrapartum-related hypoxia was the leading cause of perinatal mortality in this setting, accounting for 35.4% of all cases (Figure 2 and Supplementary Table 2). This is followed by respiratory distress syndrome (8.9%) and meconium aspiration pneumonia (7.6%). Cause of death in 43.2% could not

For peer review only - http://bmjopen.bmj.com/site/about/guidelines.xhtml

be ascertained: 15.4% for neonatal deaths, 49.2% for fresh stillbirths and 88.0% for macerated stillbirths. However, the proportion of deaths for which cause of death could not be established ("cause unknown") decreased from 34.1% (overall) in 2008 to 2.7% in 2013.

Case fatality rates:

Figure 3 and Supplementary Table 3 show the trend of case fatality rates. Over the years, a downward trend in fatality rates was observed for intrapartum-related hypoxia and complications of prematurity. However, case fatality rates for infection showed a slight increase.

Quality improvement:

More than half of the cases (53%) were adjudged to have had optimal care. The rest had probably acceptable care (21.7%), probably sub-optimal care (16.6%) or sub-optimal care (8.7%) (Supplementary Table 4). Among the different types of mortalities, sub-optimal care was most common among early neonatal deaths the most (11.8%), as compared to fresh stillbirth (6.8%) and macerated stillbirth (5.0%).

Table 2 summarizes key recommendations for action agreed as a result of perinatal death audit. In 2007 and 2008, the perinatal death review team observed many cases of delay between the decision for, and actual conduct of, Caesarean Section, which was associated with high numbers intrapartum-related hypoxia. To reduce the time taken to prepare mothers for the procedure, the team recommended the involvement of doctors in the preparation. This ensured mothers were catheterized and blood samples were taken for grouping and cross-matching at the time of decision. During the same period, a new dedicated operating theatre was built close to the maternity ward.

For peer review only - http://bmjopen.bmj.com/site/about/guidelines.xhtml

Table 2: Summary of key recommendations from audit process and time of implementation

Problem identified	Action(s) taken	Time recommendation was made	Time of implementation
Delayed referral from the lower health units	Discussed problem with referring health facilities.	September 2008	February 2009
Delay in performing Caesarean section when needed	Doctors got involved in preparing mothers before Caesarean section: they assisted in catheterisation of the mothers; taking blood sample for grouping and cross-matching; calling anesthetist and even wheeling mothers to theatre.	March 2008	June 2008
	Built a separate maternity theatre near the labour ward	March 2008	January 2014
Poor neonatal resuscitation skills among maternity staff	30 midwives, 12 intern doctors and 18 residents are trained in neonatal resuscitation every three months/in-service training.	February 2008	June 2008 (Ongoing)
	Midwives and junior doctors conduct resuscitation with skilled personnel like neonatologist, pediatricians and pediatric residents to further enhance skills. Paediatricians/residents assist during high risk deliveries in theatre and labour ward.	November 2009	December 2013
	Procured resuscitation equipment, including Ambu bags and masks and resuscitaires placed in labour ward and theatre	March 2008	October 2008 & March 2014
Poor use of the partograph and fetal heart monitoring	Trained all the midwives in the use of the partograph. Quality of partograph use monitored during the audit sessions.	January 2008	March 2008 (Ongoing)
	Increased number of staff available to complete partograph in the labour ward.	February 2009	October 2009 (Ongoing)
Hypothermia and poor management of preterm babies	Introduced kangaroo mother care for stable, preterm babies.	April 2008	September 2009
	Encouraged skin-to-skin contact at birth; LED phototherapy.	April 2008	August 2012
	Introduced continuous positive air way pressure (CPAP)	October 2008	April 2009
	Encouraged dexamethasone use for mothers in preterm labor	January 2009	October 2010
	Procured radiant warmers for the theatre, labour ward and newborn unit	April 2008	January 2014
	Made surfactant available	June 2009	February 2014

For peer review only

For peer review only - http://bmjopen.bmj.com/site/about/guidelines.xhtml

In 2008, two major factors contributing to perinatal mortality were identified to have recurred consistently: poor neonatal resuscitation and poor use of the partographs. Both of these were thought to contribute to perinatal death due to intrapartum-related hypoxia. To tackle the problem of poor resuscitation skills, maternity staff were re-trained in resuscitation and mentored by senior more staff. In addition, senior staff were involved earlier in deliveries among high-risk patients. The hospital also acquired more resuscitation equipment, including dedicated Ambu bags, masks and Resuscitaires for the labour ward and theatre. Midwives were re-trained in the use of the partograph. Human resource challenges were addressed with more midwives assigned to the maternity unit.

Training in neonatal resuscitation was done on a three-monthly basis. In 2007, asphyxiated babies were only given 10% dextrose. In subsequent years after the training, the care was expanded to include providing warmth, stimulation and ventilation with Ambu bag and provision of oxygen. This was implemented both in the labour wards and theatre.

In 2009, the case fatality rate for complications of prematurity was observed to begin to rise (Figure 3). This prompted action to address the problem of hypothermia, which was observed to be related to many of the cases. The team introduced Kangaroo Mother Care (KMC) for stable preterm babies. They also recommended the administration of dexamethasone to mothers at risk of preterm birth, and use of radiant warmers in the theatre, labour room and the newborn unit to improve thermal control. For babies with respiratory distress, Continuous Positive Airway Pressure (CPAP) was introduced, and rescue surfactant was made available for use in 2010.

In 2012, light-emitting diode phototherapy was introduced for the management of babies with neonatal jaundice. No additional specific actions to improve the management of neonatal infections were documented as this was not considered a major cause of mortality when compared to intrapartum-related hypoxia and complications of prematurity. However, all interventions were observed to be sustained throughout the period under study.

DISCUSSION

Main findings

For peer review only - http://bmjopen.bmj.com/site/about/guidelines.xhtml

For peer review only

This study explores the effectiveness of the use of perinatal death audit to improve the quality of perinatal care and perinatal outcomes at a tertiary hospital in Uganda over a period of 10 years. The perinatal mortality rate decreased from 48.4/1000 births in 2006 to 43/1000 births in 2015. Stillbirth rate decreased from 32.9/1000 births to 22/1000 births. However, the early neonatal mortality rate showed an increase from 16 to 21 per 1000 live births.

Intrapartum-related hypoxia was the leading cause of perinatal mortality (accounting for 35.4%), followed by respiratory distress syndrome (8.9%) and meconium aspiration pneumonia (7.6%). Cause of death was unknown in 43.2% of cases. The proportion of deaths with unknown cause decreased from 34.1% (overall) in 2008 to 2.7% in 2013.

More than half of the cases (53%) were adjudged to have had optimal care, with the rest of the cases having varying levels of acceptability. Early neonatal deaths had the highest proportion of cases with sub-optimal care (11.8%). Case fatality rates decreased between 2006 and 2015 for intrapartum-related hypoxia (21.3% to 15.5%) and for complications of prematurity (26.4% to 11.1%). However, case fatality rates for infection increased from 1.9% in 2006 to 5.7% in 2015.

Strengths and limitations

This study has demonstrated how the introduction of perinatal death audit helped to identify the main causes of death and contributing factors and priorities action. In the study period, quality of care improved, perinatal mortality reduced and CFR for intrapartum-related hypoxia and prematurity decreased. It is hoped that this would encourage more health care providers to undertake perinatal death reviews, especially in low-resource settings where the majority of global perinatal mortality occurs [8,9].

While it is difficult to attribute all changes in perinatal outcomes to perinatal death audit alone, the process has helped to identify priority areas for improvement in care.

There are few studies exploring the use of perinatal death audit to improve care in low-resource settings. In a similar study of 305 perinatal deaths in Tanzania, suboptimal care was identified in 80% of the cases. However, there was no report of changes implemented or on the effect of the changes [10].

Implications for future practice and research

For peer review only - http://bmjopen.bmj.com/site/about/guidelines.xhtml

Problems identified through audit:

Perinatal death audit was integrated as part of routine care to help identify deficiencies in the clinical care pathway and to formulate priority actions to ensure improved quality of care and reduced loss of life. Poor use of the partograph and fetal heart monitoring, delay in performing cesarean sections when these were indicated, poor neonatal resuscitation skills, and, poor management of preterm babies, were identified as areas requiring improvement in the quality of care. In addition, there was evidence of significant delays incurred when a referral from a lower level healthcare facility occurred. These findings are similar to a study from Tanzania where 47% of perinatal deaths audited had poor fetal heart monitoring, and 23% had delays in performing Caesarean section [10,11].

The interventions for improvement included: training in the use of the partograph, fetal heart monitoring, neonatal resuscitation, measures to reduce delay in performing a cesarean section by involving doctors in the timely preparation of mothers before transfer to the theatre and the building of a separate maternity theatre. Training in neonatal resuscitation has been conducted in Malaysia and Malawi with similar success in the acquisition of skills [12,13].

Interventions for prematurity-related complications:

The interventions implemented included: reduction of hypothermia through the provision of radiant warmers and incubators for the critically-ill babies in the labour wards, theatre and the newborn unit. KMC and immediate skin-to-skin care at birth were initiated in 2009 and 2012, respectively. The use of skin-to-skin care was noted to improve from 40% to 80% of live births after repeated training. KMC has been adopted and is used for all stable low birth weight babies in the newborn unit. A Cochrane systematic review has established that KMC is associated with reduced risk of neonatal mortality (by 51%), nosocomial infection or sepsis (by 58%) and hypothermia (by 77%) [14]. The interventions for reduction of respiratory distress included: use of antenatal steroids, bubble CPAP and use of rescue surfactant. Antenatal steroids, bubble CPAP and rescue surfactant have been found to have low coverage in low-resource settings despite their potential to reduce mortality by 48%, 31% and 32%, respectively [15,16,17].

Intrapartum-related hypoxia and prematurity-related deaths:

For peer review only - http://bmjopen.bmj.com/site/about/guidelines.xhtml

In this study, the case fatality rate for intrapartum-related hypoxia was reduced from 21.3% to 15.5%. For stillbirth, intrapartum-related hypoxia has been reported to account for between 3.1% and 25% of all cases of stillbirths in low- and middle-income countries [18]. The change may be attributable to improvement in resuscitation practices, following training and provision of equipment. Training in neonatal resuscitation has been reported to reduce deaths in babies with intrapartum-related hypoxia by 30% and early neonatal deaths by 38% [19,20]. An analysis of seven facility-based studies estimated that training in neonatal resuscitation reduced the neonatal mortality rate from 43% to 17% (14, 16-21). However, a study in Malawi reported that resuscitation training did not reduce mortality [13]. In Malawi, factors such as availability and replacement of resuscitation equipment and mentorship after training were not part of the improvement package. Additionally, in our study, the training was repeated on a three-monthly basis and continuous feedback and mentoring were provided which helped ensure continuous improvement in quality.

It is noteworthy that intrapartum-related hypoxia was documented as a cause of death even for macerated stillbirth. Since maceration could set in only six hours post-mortem [21], it is not uncommon in low-resource settings to find a macerated intrapartum death due to delays in intervention during labour. It could also be because healthcare providers often misclassify fresh stillbirth as macerated stillbirth [22].

Other factors that might have contributed to the reduction in case fatality rates were partograph use, improved fetal heart monitoring and having a dedicated maternity theatre to reduce waiting times for Caesarean section.

The case fatality rate for prematurity was reduced by more than half. One of the major interventions was the introduction of KMC, which has been shown to reduce mortality and morbidity in low-resource settings by 40% and 34%, respectively [14]. Moreover, CPAP was also introduced, which has been reported to improve survival of preterm babies by 27% among resource-limited settings [23]. Finally, in 2014 surfactant administration was introduced, which may have further reduced the mortality in prematurity-related deaths. Although surfactant is not yet provided in many developing countries as demonstrated in this study, it can be given safely under the supervision of an experienced personnel.

This study suggests that a significant reduction in case fatality rate for intrapartum-related hypoxia can be expected to occur after the implementation of multiple interventions to improve the inpatient care of

For peer review only - http://bmjopen.bmj.com/site/about/guidelines.xhtml

small and sick newborns. It further confirms the findings of a Delphi study that estimated supportive care in a Special Care Baby Unit could avert 70% of neonatal deaths due to preterm birth complications, and that 90% could be averted with the availability of Neonatal Intensive Care Units (NICUs) [24].

The fact that only 2.3% of early neonatal deaths were attributed to infection is a surprising finding. In another study, infections accounted for around 14% of early neonatal deaths [25]. In this study, 15.4% of early neonatal deaths had an unknown cause of death. It is possible that this represents an underestimation of the proportion of deaths attributable to infection. In addition, some cases of respiratory distress syndrome may actually have been cases of neonatal infection, as it is often very difficult to differentiate between the two conditions without sophisticated diagnostic tests.

The relatively large sample size in this study provided a good opportunity to document the distribution of the causes and factors contributing to perinatal mortality. Nevertheless, in 43% of the deaths, a cause of death could not be established. This proportion was highest for macerated stillbirths (88% unknown) with 49% of fresh stillbirths and 15% of early neonatal deaths assigned to the category "unknown". This may be related to the availability of information about the deaths: the longer the baby died before birth, the more difficult it becomes to obtain information about the circumstances of the death. Furthermore, diagnosis is easier for newborns than for stillbirths since clinicians usually have more time to examine and monitor a sick newborn baby during the period of admission and before death. Another possible reason for the inability to establish the cause of death may be the lack of diagnostic capacity, relating to both staff and equipment. To reduce the proportion of deaths with an unknown cause in low-resource settings, health care providers should keep clinical records as complete and as accurate as possible.

<u>Effectiveness of perinatal death audit:</u>

More and more studies have demonstrated that there is some gain in practising perinatal death audit. The population-based cohort study of 943 perinatal mortalities that occurred in 90 hospitals in The Netherlands showed how introduction of perinatal death audit resulted in a 50% reduction in substandard care (from 10% to 5%) and a lower but significant reduction in perinatal mortality (from 2.3 to 2.0/1000 births; $p<0.00001$) [5]. An earlier meta-analysis also reported a 30% reduction in perinatal mortality after introduction of perinatal death audit in healthcare facilities [6].

For peer review only - http://bmjopen.bmj.com/site/about/guidelines.xhtml

Moreover, a Cochrane review of 140 studies concluded that the audit could lead to improvements in professional practice, but this depends on how well facilities perform at baseline and how the feedback is provided [26].

Although perinatal death audit can be effective in improving quality of care, there are some reports where this did not improve perinatal outcomes. In a five-year study of health facilities in South Africa, it was observed that among the 54 health facilities that started perinatal death audit, only 30% had a decrease in perinatal mortality, while 35% had an increase [27]. The study identified several facility-related factors that contributed to the increase in perinatal mortality, including lack of use of antenatal steroids, lack of nursing personnel, fetal distress not detected during the antepartum period and incorrect use of the partograph.

More data is needed to enable identification of the exact role of audit in improving maternal and newborn health outcomes and services. Further research should also aim to determine the proportion of perinatal deaths that should be reviewed in order to have a representative view of areas of care that need immediate attention.

CONCLUSION

This study highlights the potential of perinatal death review to identify how care pathways can be improved over time to reduce adverse perinatal outcomes in low-resource settings. Healthcare providers in low-resource settings should be encouraged to conduct reviews. There is the need to improve clinical documentation to reduce the proportion of perinatal deaths with an unknown cause.

For peer review only - http://bmjopen.bmj.com/site/about/guidelines.xhtml

DECLARATIONS

Ethical approval and consent to publish and patient involvement

Approval for the study was obtained from the Nsambya Hospital Ethical Review Committee. Individual patient consent was not applicable as the research team extracted anonymized data from case notes.

Acknowledgements

We would like to thank all healthcare providers who took part in the review process. We particularly appreciate the contributions of Dr. Musana Othniel, Dr. Sebunya Robert, Dr. Nazziwa Rita, Dr. Catherine Nyagabyaki, Sr. Nabwami Immaculate, Dr. Tumwine Gilbert and Dr. Zaake.

We are also grateful to Robert Lloyd for his contribution to data processing, to Prof. Mathews Mathai for reviewing the manuscript and to Caroline Hercod for editing the manuscript.

Disclosure of Interests

All authors declare no competing interests.

Funding

No funding was received for the data collection. However, the cost of data processing, analysis and publication was sponsored by the 'Making it Happen' Programme funded by DFID/UKAid.

Availability of data and materials

The datasets used and/or analysed during the current study are available from the corresponding author upon reasonable request.

Contributions to Authorship

VN contributed to study design, data collection, analysis and writing up. MA processed and analyzed the data and contributed to all drafts of the paper. JD contributed to the conception of the paper as well as writing of the paper. RB took part in writing the study proposal, data collection and review of cases. PO

For peer review only - http://bmjopen.bmj.com/site/about/guidelines.xhtml

contributed to the study proposal, data collection and case reviews. NVDB oversaw the conception and analysis and contributed to the initial and final drafts of the paper.

REFERENCES

1. World Health Organization. WHO Fact Sheet Stillbirths. World Health Organization: Geneva, 2015.
2. Uganda Ministry of Health, Reproductive Health Division. Annual Maternal and Perinatal Death Review Report 2013/2014. Ministry of Health: Kampala, 2014.
3. Uganda Bureau of Statistics. Uganda Demographic and Health Survey 2011. Uganda Bureau of Statistics: Kampala 2011. https://dhsprogram.com/pubs/pdf/fr264/fr264.pdf
4. Buchmann EJ. Towards greater effectiveness of perinatal death audit in low- and middle-income countries. *BJOG*. 2014;**121**(Supp 4):S134-6. doi: 10.1111/1471-0528.12904
5. Eskes M, Waelput AJM, Erwich JJHM, *et al*. Term perinatal mortality audit in the Netherlands 2010–2012: a population based cohort study. *BMJ Open*. 2014;**4**:e005652. doi: 10.1136/bmjopen-2014-005652
6. Pattinson R, Keber K, Waiswa P, *et al*. Perinatal Mortality Audit: Counting, accountability, and overcoming challenges in scaling up in low- and middle-income countries. *Int J Gynaecol Obstet*. 2009;**109**(Supp 1):113-22. doi: 10.1016/j.ijgo.2009.07.011.
7. World Health Organization. ICD-10 International statistical classification of diseases and related health problems. World Health Organization: Geneva, 2004.
8. Lawn JE, Blencowe H, Pattinson R, *et al*. Stillbirths: Where? When? Why? How to make the data count? *Lancet*. 2011;**377**:1448-63. doi: 10.1016/S0140-6736(10)62187-3
9. Lawn JE, Blencowe H, Waiswa P, *et al*. Stillbirths: rates, risk factors, and acceleration towards 2030. *Lancet*. 2016;**387**:587-603. doi: 10.1016/S0140-6736(15)00837-5.
10. Kidanto HL, Mogren I, van Roosmalen J, *et al*. Introduction of a qualitative perinatal audit at Muhimbili National Hospital, Dar es Salaam, Tanzania. *BMC Pregn Childb*. 2009;**9**:45. doi: 10.1186/1471-2393-9-45
11. Kidanto HL, Massawe SN, Nyström L, *et al*. Analysis of perinatal mortality at a teaching hospital in Dar es Salaam, Tanzania. *Afr J Reprod Health*. 2006;**10**:72-80.
12. Boo NY. Neonatal resuscitation programme in Malaysia: an eight-year experience. *Singapore Med J*. 2009;**50**:152-9.
13. Hole MK, Olmsted K, Kiromera A, *et al*. Neonatal Resuscitation Curriculum in Malawi, Africa: Did It Change In-Hospital Mortality? *Int J Pediatr*. 2012;**2012**:408689. doi: 10.1155/2012/408689
14. Conde-Agudelo A, Belizán JM, Diaz-Rossello J. Kangaroo mother care to reduce morbidity and mortality in low birthweight infants. *Cochrane Database Syst Rev*. 2011;**3**:CD002771. doi: 10.1002/14651858.CD002771.pub2.
15. Mwansa-Kambafwile J, Cousens S, Hansen T, *et al*. Antenatal steroids in preterm labour for the prevention of neonatal deaths due to complications of preterm birth. *Int J Epidemiol*. 2010;**39**(Supp 1):i122–33. doi: 10.1093/ije/dyq029.
16. Ho JJ, Subramaniam P, Henderson-Smart DJ, *et al*. Continuous distending pressure for respiratory distress syndrome in preterm infants. *Cochrane Database Syst Rev*. 2002;**2**:CD002271.
17. Soll R, Özek E. Prophylactic animal derived surfactant extract for preventing morbidity and mortality in preterm infants. *Cochrane Database Syst Rev*. 2002;**4**:CD000511.
18. Aminu M, Unkels R, Mdegela M, *et al*. Causes of and factors associated with stillbirth in low- and middle-income countries: a systematic literature review. *BJOG*. 2014;**121**(Supp 4):141-53. doi: 10.1111/1471-0528.12995.

For peer review only - http://bmjopen.bmj.com/site/about/guidelines.xhtml

19. Lawn J, Shibuya K, Stein C. No cry at birth: Global estimates of intrapartum stillbirths and intrapartum-related neonatal deaths. *Bull World Health Organ*. 2005;**83**:409-17.
20. Lee ACC, Cousens S, Wall SN, *et al*. Neonatal resuscitation and immediate newborn assessment and stimulation for the prevention of neonatal deaths. *BMC Public Health*. 2011;**11**(Supp 3):S12. doi: 10.1186/1471-2458-11-S3-S12
21. Genest DR, Singer DB. Estimating the time of death in stillborn fetuses: III. External fetal examination; a study of 86 stillborns. *Obstet Gynecol*. 1992;**80**:593-600.
22. Gold KJ, Abdul-Mumin AS, Boggs ME, *et al*. Assessment of “fresh” versus “macerated” as accurate markers of time since intrauterine fetal demise in low income countries. *Int J Gynaecol Obstet*. 2014;**125**:223-7. doi: 10.1016/j.ijgo.2013.12.006
23. Kawaza K, Machen HE, Brown J, *et al*. Efficacy of a low-cost bubble CPAP system in treatment of respiratory distress in a neonatal ward in Malawi. *PLoS One*. 2014;**9**:e86327. doi: 10.1371/journal.pone.0086327
24. Bhutta ZA, Das JK, Bahl R, *et al*. Can available interventions end preventable deaths in mothers, newborn babies, and stillbirths, and at what cost? *Lancet*. 2014;**384**:347-70. doi: 10.1016/S0140-6736(14)60792-3
25. Oza S, Lawn LE, Hogan DR, *et al*. Neonatal cause-of-death estimates for the early and late neonatal periods for 194 countries: 2000-2013. *Bull World Health Organ*. 2015;**93**:19-28. doi: 10.2471/BLT.14.139790
26. Ivers N, Jamtvedt G, Flottorp S, *et al*. Audit and feedback: effects on professional practice and healthcare outcomes. *Cochrane Database Syst Rev*. 2012;**6**:CD000259. doi: 10.1002/14651858.CD000259.pub3
27. Allanson ER, Pattinson RC. Quality-of-care audits and perinatal mortality in South Africa. *Bull World Health Organ*. 2015;**93**:424-8. doi: 10.2471/BLT.14.144683

For peer review only

For peer review only - http://bmjopen.bmj.com/site/about/guidelines.xhtml

Figure and Table Captions

Figure 1: Stillbirth rate (per 1000 births), early neonatal mortality rate (per 1000 live births) and perinatal mortality rate (per 1000 births) for the years 2006 to 2015

Figure 2: Cause of death for macerated stillbirths (n=142), fresh stillbirths (n=125) and early neonatal deaths (n=261)

Figure 3: Trend in case fatality rates (CFR) for intrapartum-related hypoxia, prematurity and infections
Figure 3 Legend: KMC=Kangaroo mother care; CPAP=Continuous positive airway pressure

Supplementary Table 1: Stillbirth rate (per 1000 births), early neonatal mortality rate (per 1000 live births) and perinatal mortality rate (per 1000 births)

Supplementary Table 2: Cause of death for stillbirth and early neonatal death

Supplementary Table 3: Case fatality rates (CFR)

Supplementary Table 4: Standard of care

For peer review only

For peer review only - http://bmjopen.bmj.com/site/about/guidelines.xhtml

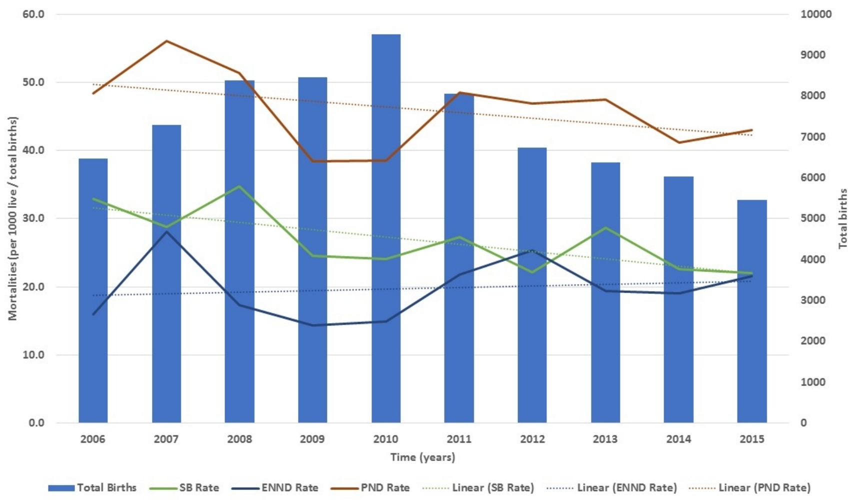

However, early neonatal mortality rate (ENMR) showed a slight increase, which declined between 2007 and 2009 only to rise again between 2010 and 2012, and progress has been slow since then (Figure 1).

338x190mm (96 x 96 DPI)

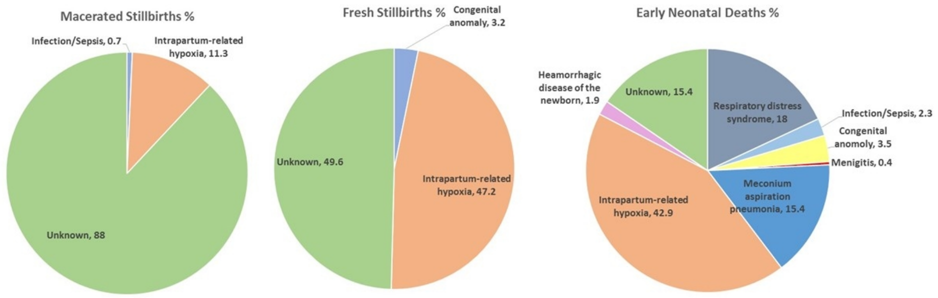

Figure 2: Cause of death for macerated stillbirths (n=142), fresh stillbirths (n=125) and early neonatal deaths (n=261)

338x190mm (96 x 96 DPI)

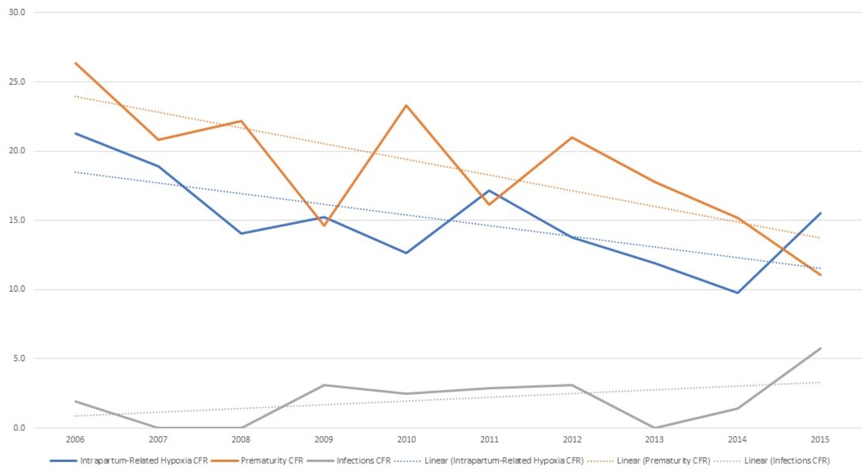

Figure 3: Trend in case fatality rates (CFR) for intrapartum-related hypoxia, prematurity and infections
Figure 3 Legend: KMC=Kangaroo mother care; CPAP=Continuous positive airway pressure

338x190mm (96 x 96 DPI)

Supplementary Materials

Supplementary Table 1: Stillbirth rate (per 1000 births), early neonatal mortality rate (per 1000 live births) and perinatal mortality rate (per 1000 births).

Year	Total Births	Stillbirth Rate	Early NND Rate	Perinatal Mortality Rate
2006	6468	32.9	16.0	48.4
2007	7290	28.8	28.1	56.1
2008	8386	34.7	17.3	51.4
2009	8452	24.5	14.3	38.5
2010	9513	24.1	14.9	38.6
2011	8050	27.3	21.8	48.6
2012	6732	22.1	25.4	46.9
2013	6376	28.7	19.4	47.5
2014	6026	22.6	19.0	41.2
2015	5462	22.0	21.5	43.0

Supplementary Table 2: Cause of death for stillbirth and early neonatal death.

Cause of Death	Macerated Stillbirth n=142 (%)	Fresh Stillbirth n=125 (%)	Early Neonatal Death n=259 (%)	TOTAL n=526 (%)
Respiratory distress syndrome	-	-	47 (18.0)	**47 (8.9)**
Infections / Septicaemia	1 (0.7)	0	6 (2.3)	**7 (1.3)**
Congenital anomaly	0	4 (3.2)	9 (3.5)	**13 (2.5)**
Meningitis	-	-	1 (0.4)	**1 (0.2)**
Meconium aspiration pneumonia	-	-	40 (15.4)	**40 (7.6)**
Intrapartum-related hypoxia	16 (11.3)	59 (47.2)	111 (42.9)	**186 (35.4)**
Haemorrhagic disease of newborn	-	-	5 (1.9)	**5 (1.0)**
Unknown	125 (88.0)	61 (49.6)	40 (15.4)	**227 (43.2)**

For peer review only

Supplementary Table 3: Case fatality rates (CFR)

Cause of death		2006	2007	2008	2009	2010	2011	2012	2013	2014	2015
**Intrapartum-related hypoxia**	Intrapartum-related hypoxia admissions	202	291	334	302	316	297	247	252	195	187
	Intrapartum-related hypoxia deaths	43	55	47	46	40	51	34	30	19	29
	**Intrapartum-related hypoxia CFR (%)**	**21.3**	**18.9**	**14.1**	**15.2**	**12.7**	**17.2**	**13.8**	**11.9**	**9.7**	**15.5**
**Prematurity**	Prematurity admissions	163	168	194	246	210	328	286	304	336	371
	Premature deaths	43	35	43	36	49	53	60	54	51	41
	**Prematurity CFR (%)**	**26.4**	**20.8**	**22.2**	**14.6**	**23.3**	**16.2**	**21.0**	**17.8**	**15.2**	**11.1**
**Infections**	Number of admissions for infections	104	149	82	128	120	140	129	171	141	87
	Deaths due to infections	2	0	0	4	3	4	4	0	2	5
	**Infections CFR (%)**	**1.9**	**0**	**0**	**3.1**	**2.5**	**2.9**	**3.1**	**0.0**	**1.4**	**5.7**

Supplementary Table 4: Standard of care

**Standard of care**	**MSB (%)**	**FSB (%)**	**ENND (%)**	**Total (%)**
Optimal	82 (67.8)	58 (49.2)	112 (45.7)	262 (53.0)
Probably acceptable	21 (17.4)	28 (23.7)	58 (23.7)	107 (21.7)
Probably sub-optimal	12 (9.9)	24 (20.3)	46 (18.8)	82 (16.6)
Sub-optimal	6 (5.0)	8 (6.8)	29 (11.8)	43 (8.7)
**Total**	**121 (100)**	**118 (100)**	**245 (100)**	**494 (100)**

For peer review only

BMJ Open

Prospective Study to Explore Changes in Quality of Care and Perinatal Outcomes After Implementation of Perinatal Death Audit in Uganda

Journal:	*BMJ Open*
Manuscript ID	bmjopen-2018-027504.R1
Article Type:	Research
Date Submitted by the Author:	31-May-2019
Complete List of Authors:	Kirabira, Victoria; Nsambya Hospital<br>Aminu, Mamuda; Liverpool School of Tropical Medicine, Centre for Maternal and Newborn Health ; Liverpool School of Tropical Medicine<br>Dewez, Juan; Liverpool School of Tropical Medicine, Centre for Maternal and Newborn Health<br>Byaruhanga, Romano; Nsambya Hospital<br>Okong, Pius; Nsambya Hospital<br>van den Broek, Nynke; Liverpool School of Tropical Medicine, Centre for Maternal and Newborn Health
**Primary Subject Heading**:	Obstetrics and gynaecology
Secondary Subject Heading:	Global health
Keywords:	OBSTETRICS, AUDIT, Reproductive medicine < GYNAECOLOGY, Clinical audit < HEALTH SERVICES ADMINISTRATION & MANAGEMENT, Quality in health care < HEALTH SERVICES ADMINISTRATION & MANAGEMENT

SCHOLARONE™
Manuscripts

I, the Submitting Author has the right to grant and does grant on behalf of all authors of the Work (as defined in the below author licence), an exclusive licence and/or a non-exclusive licence for contributions from authors who are: i) UK Crown employees; ii) where BMJ has agreed a CC-BY licence shall apply, and/or iii) in accordance with the terms applicable for US Federal Government officers or employees acting as part of their official duties; on a worldwide, perpetual, irrevocable, royalty-free basis to BMJ Publishing Group Ltd ("BMJ") its licensees and where the relevant Journal is co-owned by BMJ to the co-owners of the Journal, to publish the Work in this journal and any other BMJ products and to exploit all rights, as set out in our licence.

The Submitting Author accepts and understands that any supply made under these terms is made by BMJ to the Submitting Author unless you are acting as an employee on behalf of your employer or a postgraduate student of an affiliated institution which is paying any applicable article publishing charge ("APC") for Open Access articles. Where the Submitting Author wishes to make the Work available on an Open Access basis (and intends to pay the relevant APC), the terms of reuse of such Open Access shall be governed by a Creative Commons licence – details of these licences and which Creative Commons licence will apply to this Work are set out in our licence referred to above.

Other than as permitted in any relevant BMJ Author's Self Archiving Policies, I confirm this Work has not been accepted for publication elsewhere, is not being considered for publication elsewhere and does not duplicate material already published. I confirm all authors consent to publication of this Work and authorise the granting of this licence.

For peer review only

Prospective Study to Explore Changes in Quality of Care and Perinatal Outcomes After Implementation of Perinatal Death Audit in Uganda

---

Victoria Nakibuuka Kirabira[a], Mamuda Aminu[b§], Juan Emmanuel Dewez[b], Romano Byaruhanga[a], Pius Okong[a], Nynke van den Broek[b]

[a] Nsambya Hospital, P.O. Box 7146, Kampala, Uganda

[b] Centre for Maternal and Newborn Health, Liverpool School of Tropical Medicine, Pembroke Place, Liverpool L3 5QA, UK

[§]Corresponding Author Contact Details:

Dr Mamuda Aminu
Centre for Maternal and Newborn Health,
Liverpool School of Tropical Medicine, Pembroke Place, Liverpool L3 5QA.
Tel: +44(0)151 705 3342
Email: Mamuda.Aminu@lstmed.ac.uk

Author email addresses:

VN Kirabira	nakibuukarv@yahoo.com
JE Dewez	manuel.dewez@lshtm.ac.uk
R Byaruhanga	byaruhangarn@yahoo.com
P Okong	puisokong@yahoo.com
NR van den Broek	Nynke.Broek@lstmed.ac.uk

Running Title: Perinatal death audit in Uganda

Keywords: perinatal death, cause of perinatal death, quality improvement

***

Word Count (Body Only): 3680

Abstract: 300

For peer review only - http://bmjopen.bmj.com/site/about/guidelines.xhtml

ABSTRACT

Objective: To assess the effects of perinatal death audit on perinatal outcomes in a tertiary hospital in Kampala

Design: Interrupted time series (ITS) analysis

Setting: Nsambya Hospital, Uganda

Participants: Live births and stillbirths

Interventions: Perinatal death audit

Primary and secondary outcome measures: Primary outcomes: perinatal mortality rate, stillbirth rate, early neonatal mortality rate. Secondary outcomes: case fatality rates (CFR) for asphyxia, complications of prematurity and neonatal sepsis.

Results

526 perinatal deaths were included: 142 (27.0%) fresh stillbirths, 125 (23.8%) macerated stillbirths and 259 (49.2%) early neonatal deaths.

The ITS analysis showed a decrease in perinatal death (PND) rates without the introduction of perinatal death audits (IRR (95% CI) for time=0.94, p<0.001), but an increase in PND (IRR (95% CI) =1.17 (1.0,-1.34), p=0.0021) following the intervention. However, when over-dispersion was included in the model, there were not statistically significant differences in PND with or without the intervention (p=0.06 and p=0.44 respectively).

Stillbirth rates exhibited a similar pattern. By contrast, early neonatal death rates showed an overall upward trend without the intervention (IRR (95% CI)= 1.09 (1.01-1.17), p=0.01), but a decrease following the introduction of the perinatal death audits (IRR (95% CI)= 0.35 (0.22-0.56), p<0.001), when over-dispersion was included.

The CFR for prematurity showed a downward trend over time (IRR (95% CI) =0.94 (0.88-0.99), p=0.04) but not for the intervention. With regards CFRs for intrapartum-related hypoxia or infection, no statistically significant effect was detected for either time or the intervention.

Conclusion

The introductuion of perinatal death audit showed no statistically significant effect on perinatal mortality or stillbirth rate, but a significantly decrease in early neonatal mortality rate. No effect was detected on case fatality rates for prematurity, intra-partum-related hypoxia or infections.

For peer review only - http://bmjopen.bmj.com/site/about/guidelines.xhtml

ARTICLE SUMMARY

- The study assesses the effect of introducing perinatal death audit on perinatal outcomes in a tertiary hospital in Uganda.
- Perinatal death audit helped to identify priority areas for which action was taken to improve the quality of care.
- Early neonatal death rates decreased as a result of the intervention (IRR (95% CI)= 0.35 (0.22-0.56), p<0.001). The intervention did not had an effect on perinatal death rates, nor stillbirth rates.

Strengths and limitations of this study

- This study is one of the few studies assessing the effect of perinatal death audits in a resource-limited setting.
- The interrupted time series design was useful to assess the effect of the intervention, but additional studies, such as stepped wedge randomised controlled trials, must confirm the findings of this study.

For peer review only

For peer review only - http://bmjopen.bmj.com/site/about/guidelines.xhtml

BACKGROUND

Currently, about 2.7 million neonatal deaths and 2.6 million stillbirths occur worldwide every year. Of these, the majority occur in low- and middle-income countries, and more than half (55%) of the global stillbirths occur in sub-Saharan Africa and South Asia [1]. Uganda's perinatal mortality is estimated to be 36 deaths per 1000 births and remains unacceptably high. Almost 50% of these are fresh stillbirths [2]. The neonatal mortality rate (21 per 1000 live births) in Uganda is also high, contributing 50% of the infant mortality rate [3]. It is crucial that interventions are put in place to reduce preventable neonatal deaths and stillbirths.

Perinatal death audit (or review) can be used to identify areas of care which can be strengthened to improve perinatal outcomes [4]. It involves reviewing cases of mortality with a view to identifying avoidable factors and implementing changes to prevent future deaths. A population-based cohort study of 943 perinatal mortalities that occurred in 90 hospitals in The Netherlands reported a decrease in substandard care (from 10% to 5%) and perinatal mortality (from 2.3 to 2.0/1000 births; $p<0.00001$) after the introduction of perinatal death audit [5].

Similarly, in a systematic review focusing on low- and middle-income countries, a meta-analysis of 7 before-and-after studies reported a 30% (95% confidence interval, 21%–38%) reduction in perinatal mortality after introduction of perinatal death audit in healthcare facilities [6]. There is, however, a paucity of data from low-resource settings on the use of and effectiveness of perinatal death audit to improve quality of care provided to mothers and their babies. The systematic review recommended more research in key areas, including exploring the effect of facility-based perinatal death audit on changing the quality of care [6].

The aim of this study is to assess the changes in perinatal outcomes observed after the introduction of perinatal death audit in a tertiary hospital in Uganda, and to describe the changes in healthcare provided to mother and babies resulting from the perinatal death audits.

METHODS

Study setting

For peer review only - http://bmjopen.bmj.com/site/about/guidelines.xhtml

This study was conducted at Nsambya Hospital, a private not-for-profit tertiary hospital in Kampala, Uganda, which serves as a referral centre for Makindye Health Sub-district in Kampala. The facility receives 7,000 deliveries per year and provides 24-hour comprehensive emergency obstetric and newborn care services, with a staff of 6 obstetricians, 18 resident doctors, 5 intern doctors and 40 midwives.

The newborn care unit admits 2,500 babies annually and has a paediatrician/neonatologist, two residents and up to four midwives on duty per shift. It has 40 beds, including 10 neonatal intensive beds, and provides essential newborn care including; oxygen therapy, surfactant and low-cost bubble continuous positive airway pressure (CPAP), kangaroo mother care and phototherapy.

Data collection

Perinatal death audit using locally developed tools and guidelines started in 2008 and was led by a multidisciplinary audit team comprising of obstetricians, paediatricians, resident doctors, midwives and administrators. Team meetings were held weekly, chaired by the most senior obstetrician or paediatrician. Perinatal death was defined as "death of a fetus greater than a birth weight of 1000g if measured, or a gestational age of at least 28 completed weeks or more, or a body length (crown-heel) of at least 35cm or more"[7] and included all stillbirths and deaths within the first week of life (early neonatal deaths). The number of perinatal deaths was documented daily using clinical handover reports and registers in the labour ward and neonatal special care unit. Due to large numbers of death, one-quarter of the cases (every 4th case) were selected for audit from each of the following categories: fresh stillbirths, macerated stillbirths and early neonatal deaths.

The number of admissions in the newborn unit, reasons for admission, death and case fatalities rates for intrapartum-related hypoxia, infection and prematurity were collected for the period before the perinatal audit was introduced (2006-2007) and subsequently for the period after commencement of the perinatal death audit (2008-2015). The data was obtained and aggregated monthly through a retrospective analysis of registers and patient records. Numbers of live and total births were also documented to enable rates calculation.

Intervention: the audit process

For each case selected for review, an audit form was completed by a resident doctor, who also provided a narrative summary for each case. Sources of information included: clinical handover books, maternity

For peer review only - http://bmjopen.bmj.com/site/about/guidelines.xhtml

registers, newborn admission register, and clinical notes for mothers and newborns. Each case was then presented to the rest of the audit team for detailed discussion. Cause of death was identified by consensus, and care provided was judged against existing local protocols and national guidelines. No clinical autopsies were performed due to lack of a full-time pathologist at the hospital.

The quality of care provided at the hospital was also graded by consensus and classified into four categories:

- Optimal care generally accepted practice and all standards of care were followed.
- Suboptimal care but different management would have made no difference to the outcome (probably acceptable)
- Suboptimal care where different management might have made a difference to the outcome - an avoidable factor of uncertain clarity or influence on the outcome (probably suboptimal)
- Different management would reasonably be expected to have made a difference to the Outcome. A clearly avoidable factor implying that the adverse outcome could have been prevented) (suboptimal)

The overall grading of the care provided to the mother and baby, the identified specific areas of suboptimal care and the recommendations for improvement were documented. This grading included referred mothers as well, whereas there were delays from the reffering site, sub optimal care can also occur in tertiary hospital as well, such as delay in performing a cesearean section

The summary of each case included a list of the actions to be taken. In each case, a specific person was appointed to lead the implementation of the relevant actions. During monthly meetings, implementation progress was compared with the action plan by the wider audit team. A number of interventions, such as neonatal resuscitation training, introduction of KMC, building of a new maternity theater, were thus introduced gradually over the years to address the gaps in care.

Data analysis

SPSS© (IBM, version 22) was used for descriptive analyses.Stillbirth and perinatal mortality rates were expressed per 1000 total births, while early neonatal mortality rate was expressed per 1000 live births. Case fatality rate (CFR) was defined as the proportion of deaths among cases diagnosed with the disease. Statistical significance was calculated at 95% confidence interval or p-value of ≤0.05.

Data for the six primary and secondary outcomes were each analysed using interrupted time series (ITS) models [8]. describe various modifications to the model which can be considered. Over-dispersion was

For peer review only - http://bmjopen.bmj.com/site/about/guidelines.xhtml

considered (by using an additional option: scale(x2)) since it often arises in the context of count data. Auto-correlation was also inspected using plots. The 5% level was used to determine statistical significance. The ITS was performed with STATA©. For each outcome considered, the autocorrelation and partial correlation plots provided no evidence that there was autocorrelation not accounted for in the models used.

Since low birth weight and prematurity are both associated with a higher risk of perinatal mortality, a one-way between-groups analysis of variance (ANOVA) was conducted to explore changes in birthweight and gestational age over time.

A narrative summary description was used to report actions agreed and implemented following an audit. These were documented chronologically as derived from minutes of the audit meetings.

Ethical approval

Approval for the study was obtained from the Nsambya Hospital Ethical Review Committee. Individual patient consent was not applicable as the research team extracted anonymized data from case notes.

RESULTS

Demographic and clinical characteristics:

A total of 58,997 births and 2,616 perinatal deaths occurred between 2008 and 2015 (Supplementary table 1). Of these, 603 (23%) perinatal deaths were selected for audit. After review, 526 were found to meet the definition of perinatal mortality: 142 (27.0%) fresh stillbirths, 125 (23.8%) macerated stillbirths and 259 (49.2%) early neonatal deaths (Table 1).

The majority of cases came to the hospital directly, with 17% referrals . Mothers had a mean age of 27.3 years (SD: 5.1); median parity of 2 (SD of 1.6). Just over one-third of fresh stillbirths and early neonatal deaths were delivered by Caesarean section. The mean birth weight of babies was 2610 grams (SD: 969.4). Between 23.2% and 39.4% were born prematurely (Table 1).

Cause of death:

For peer review only - http://bmjopen.bmj.com/site/about/guidelines.xhtml

Overall, intrapartum-related hypoxia was the leading cause of perinatal mortality in this setting, accounting for 35.4% of all cases (Figure 1 and Supplementary Table 2). This is followed by respiratory distress syndrome (8.9%) and meconium aspiration pneumonia (7.6%). Cause of death in 43.2% could not be ascertained: 15.4% for neonatal deaths, 49.2% for fresh stillbirths and 88.0% for macerated stillbirths. However, the proportion of deaths for which cause of death could not be established ("cause unknown") decreased from 34.1% (overall) in 2008 to 2.7% in 2013.

Trend in perinatal mortality rate:

The ITS analysis showed that, without the intervention, there would have been a downward trend (a decrease) in perinatal death rates (IRR (95% CI) for time=0.94, p<0.001), while a statistically significant increase (IRR (95% CI) =1.17 (1.0,-1.34), p=0.0021) was observed following the introduction of perinatal death audits. However, when over-dispersion was included in the model, both effects were not statistically significant (Table 2, Figures 2 and 3, and Supplementary Table 3).

Stillbirth rates exhibited a similar pattern. By contrast, early neonatal death rates showed an overall upward trend without the intervention (IRR (95% CI)= 1.09 (1.01-1.17), p=0.01) but a a decrease with the introduction of the perinatal death audits (IRR (95% CI) = 0.35 (0.22-0.56), p<0.001) when over-dispersion was included in the model (Table 2, and Figures 2 and 3). The ANOVA to explore changes in birthweight and gestational age over time showed no statistically significant change in birthweight over time (p = 0.872), and no change in gestational age at birth over time (p = 0.219).

The CFR for prematurity showed a downward trend over time (IRR (95% CI)=0.94 (0.88-0.99), p=0.04) but a downward trend was not observed with the intervention. With regards the CFRs for intrapartum-related hypoxia and infections, no statistically significant effect was detected with or without the intervention (Table 2, Figures 2 and 3, and Supplementary Table 3).

Quality improvement:

More than half of the cases (53%) were deemed to have had optimal care. The rest had probably acceptable care (21.7%), probably sub-optimal care (16.6%) or sub-optimal care (8.7%) (Supplementary Table 4). Among the different types of mortalities, sub-optimal care was most common among early neonatal deaths the most (11.8%), as compared to fresh stillbirth (6.8%) and macerated stillbirth (5.0%).

For peer review only - http://bmjopen.bmj.com/site/about/guidelines.xhtml

In 2007 and 2008, the perinatal death review team observed many cases of delay between the decision for, and actual conduct of, Caesarean Section, which was associated with high numbers of intrapartum-related hypoxia. To reduce the time taken to prepare mothers for the procedure, the team recommended the involvement of doctors in the preparation. This ensured mothers were catheterized and blood samples were taken for grouping and cross-matching at the time of decision. During the same period, a new dedicated operating theatre was built close to the maternity ward.

In 2008, two major factors contributing to perinatal mortality were identified to have recurred consistently: poor neonatal resuscitation and poor use of the partographs. Both of these were thought to contribute to perinatal death due to intrapartum-related hypoxia. To tackle the problem of poor resuscitation skills, maternity staff were re-trained in resuscitation and mentored by the most senior staff. In addition, senior staff were involved earlier in deliveries among high-risk patients. The hospital also acquired more resuscitation equipment, including dedicated Ambu bags, masks and Resuscitaires for the labour ward and theatre. Midwives were re-trained in the use of the partograph. Human resource challenges were addressed with more midwives assigned to the maternity unit.

Training in neonatal resuscitation was done on a three-monthly basis. In 2007, asphyxiated babies were only given 10% dextrose. In subsequent years after the training, the care was expanded to include providing warmth, stimulation and ventilation with Ambu bag and provision of oxygen. This was implemented both in the labour wards and theatre.

In 2009, the case fatality rate for complications of prematurity was observed to begin to rise (Figure 3). This prompted action to address the problem of hypothermia, which was observed to be related to many of the cases. The team introduced Kangaroo Mother Care (KMC) for stable preterm babies. They also recommended the administration of dexamethasone to mothers at risk of preterm birth, and use of radiant warmers in the theatre, labour room and the newborn unit to improve thermal control. For babies with respiratory distress, Continuous Positive Airway Pressure (CPAP) was introduced, and rescue surfactant was made available for use in 2014.

In 2012, light-emitting diode phototherapy was introduced for the management of babies with neonatal jaundice. No additional specific actions to improve the management of neonatal infections were documented as this was not considered a major cause of mortality when compared to intrapartum-related

For peer review only - http://bmjopen.bmj.com/site/about/guidelines.xhtml

hypoxia and complications of prematurity. However, all interventions were observed to be sustained throughout the period under study.

DISCUSSION

Main findings

This study assessed the effect of perinatal death audit to improve the quality of perinatal care and perinatal outcomes in a tertiary hospital in Uganda over a period of 10 years.

Intrapartum-related hypoxia was the leading cause of perinatal mortality (accounting for 35.4%), followed by respiratory distress syndrome (8.9%) and meconium aspiration pneumonia (7.6%). Cause of death was unknown in 43.2% of cases. The proportion of deaths with unknown cause decreased from 34.1% (overall) in 2008 to 2.7% in 2013.

There was no effect of perinatal death audit on perinatal mortality or stillbirth rates. However, the interventin led to a decrease in neonatal mortality rate [IRR (95% CI)= 0.35 (0.22-0.56), p<0.001)].

The CFR for prematurity showed a downward trend over time (IRR (95% CI)=0.94 (0.88-0.99), p=0.04), but this was not related to the perinatal death audits. The CFRs for intrapartum-related hypoxia and infections were not affected by the intervention.

More than half of the cases (53%) were deemed to have had optimal care, with the rest of the cases having varying levels of acceptability. Early neonatal deaths had the highest proportion of cases with sub-optimal care (11.8%). Case fatality rates decreased between 2006 and 2015 for intrapartum-related hypoxia (21.3% to 15.5%) and for complications of prematurity (26.4% to 11.1%). However, case fatality rates for infection increased from 1.9% in 2006 to 5.7% in 2015.

Strengths and limitations

This study assessed then introduction of pernatal audits in a low resource setting. There are few studies exploring the use of perinatal death audit to improve care in low-resource settings. In a similar study of 305 perinatal deaths in Tanzania, suboptimal care was identified in 80% of the cases. However, there was no report of changes implemented or on the effect of the changes [9]. Moreove, While it is difficult to attribute all changes in perinatal outcomes to perinatal death audit alone, the process has helped to

For peer review only - http://bmjopen.bmj.com/site/about/guidelines.xhtml

identify areas of healthcare that need support for improvement in outcomes.The results should be interpreted in light of the limitations of interrupted time series as the methodology used to assess the effect of an intervention. Ideally, the results of the study should be confirmed by randomised controlled trials conducted in the same context of care.

Implications for future practice and research

Problems identified through audit:

Perinatal death audit was integrated as part of routine care to help identify deficiencies in the clinical care pathway and to formulate priority actions to ensure improved quality of care and reduced loss of life. Poor use of the partograph and fetal heart monitoring, delay in performing cesarean sections when these were indicated, poor neonatal resuscitation skills, and, poor management of preterm babies, were identified as areas requiring improvement in the quality of care. In addition, there was evidence of significant delays incurred when a referral from a lower level healthcare facility occurred. These findings are similar to a study from Tanzania where 47% of perinatal deaths audited had poor fetal heart monitoring, and 23% had delays in performing Caesarean section [9,10].

The interventions for improvement included: training in the use of the partograph, fetal heart monitoring, neonatal resuscitation, measures to reduce delay in performing a cesarean section by involving doctors in the timely preparation of mothers before transfer to the theatre and the building of a separate maternity theatre. Training in neonatal resuscitation has been conducted in Malaysia and Malawi with similar success in the acquisition of skills [11,12].

Interventions for prematurity-related complications:

The interventions implemented included: reduction of hypothermia through the provision of radiant warmers and incubators for the critically-ill babies in the labour wards, theatre and the newborn unit. KMC and immediate skin-to-skin care at birth were initiated in 2009 and 2012, respectively. The use of skin-to-skin care was noted to improve from 40% to 80% of live births after repeated training. KMC has been adopted and is used for all stable low birth weight babies in the newborn unit. A Cochrane systematic review has established that KMC is associated with reduced risk of neonatal mortality (by 51%), nosocomial infection or sepsis (by 58%) and hypothermia (by 77%) [13]. The interventions for reduction of respiratory distress included: use of antenatal steroids, bubble CPAP and use of rescue surfactant.

For peer review only - http://bmjopen.bmj.com/site/about/guidelines.xhtml

Antenatal steroids, bubble CPAP and rescue surfactant have been found to have low coverage in low-resource settings despite their potential to reduce mortality by 48%, 31% and 32%, respectively [14,15,16].

Intrapartum-related hypoxia and prematurity-related deaths:

In this study, the case fatality rate for intrapartum-related hypoxia was reduced from 21.3% to 15.5%. For stillbirth, intrapartum-related hypoxia has been reported to account for between 3.1% and 25% of all cases of stillbirths in low- and middle-income countries [17]. The change may be attributable to improvement in resuscitation practices, following training and provision of equipment. Training in neonatal resuscitation has been reported to reduce deaths in babies with intrapartum-related hypoxia by 30% and early neonatal deaths by 38% [18,19]. An analysis of seven facility-based studies estimated that training in neonatal resuscitation reduced the neonatal mortality rate from 43% to 17%. However, a study in Malawi reported that resuscitation training did not reduce mortality [12]. In Malawi, factors such as availability and replacement of resuscitation equipment and mentorship after training were not part of the improvement package. Additionally, in our study, the training was repeated on a three-monthly basis and continuous feedback and mentoring were provided which helped ensure continuous improvement in quality.

It is noteworthy that intrapartum-related hypoxia was documented as a cause of death even for macerated stillbirth. Since maceration could set in only six hours post-mortem [20], it is not uncommon in low-resource settings to find a macerated intrapartum death due to delays in intervention during labour. It could also be because healthcare providers often misclassify fresh stillbirth as macerated stillbirth [21].

Other factors that might have contributed to the reduction in case fatality rates were partograph use, improved fetal heart monitoring and having a dedicated maternity theatre to reduce waiting times for Caesarean section.

The case fatality rate for prematurity was reduced by more than half. One of the major interventions was the introduction of KMC, which has been shown to reduce mortality and morbidity in low-resource settings by 40% and 34%, respectively [13]. Moreover, CPAP was also introduced, which has been reported to improve survival of preterm babies by 27% among resource-limited settings [22]. Finally, in 2014 surfactant administration was introduced, which may have further reduced the mortality in prematurity-

For peer review only - http://bmjopen.bmj.com/site/about/guidelines.xhtml

related deaths. Although surfactant is not yet provided in many developing countries as demonstrated in this study, it can be given safely under the supervision of an experienced personnel.

This study suggests that a significant reduction in case fatality rate for intrapartum-related hypoxia can be expected to occur after the implementation of multiple interventions to improve the inpatient care of small and sick newborns. It further confirms the findings of a Delphi study that estimated supportive care in a Special Care Baby Unit could avert 70% of neonatal deaths due to preterm birth complications, and that 90% could be averted with the availability of Neonatal Intensive Care Units (NICUs) [23].

The fact that only 2.3% of early neonatal deaths were attributed to infection is a surprising finding. In another study, infections accounted for around 14% of early neonatal deaths [24]. In this study, 15.4% of early neonatal deaths had an unknown cause of death. It is possible that this represents an underestimation of the proportion of deaths attributable to infection. In addition, some cases of respiratory distress syndrome may actually have been cases of neonatal infection, as it is often very difficult to differentiate between the two conditions without sophisticated diagnostic tests.

The relatively large sample size in this study provided a good opportunity to document the distribution of the causes and factors contributing to perinatal mortality. Nevertheless, in 43% of the deaths, a cause of death could not be established. This proportion was highest for macerated stillbirths (88% unknown) with 49% of fresh stillbirths and 15% of early neonatal deaths assigned to the category “unknown”. This may be related to the availability of information about the deaths: the longer the baby died before birth, the more difficult it becomes to obtain information about the circumstances of the death. Furthermore, diagnosis is easier for newborns than for stillbirths since clinicians usually have more time to examine and monitor a sick newborn baby during the period of admission and before death. Another possible reason for the inability to establish the cause of death may be the lack of diagnostic capacity, relating to both staff and equipment. To reduce the proportion of deaths with an unknown cause in low-resource settings, health care providers should keep clinical records as complete and as accurate as possible.

Effectiveness of perinatal death audit:

More and more studies have demonstrated that there is some gain in practising perinatal death audit. The population-based cohort study of 943 perinatal mortalities that occurred in 90 hospitals in The Netherlands showed how introduction of perinatal death audit resulted in a 50% reduction in substandard care (from 10% to 5%) and a lower but significant reduction in perinatal mortality (from 2.3 to 2.0/1000

For peer review only - http://bmjopen.bmj.com/site/about/guidelines.xhtml

births; p<0.00001) [5]. An earlier meta-analysis also reported a 30% reduction in perinatal mortality after introduction of perinatal death audit in healthcare facilities [6].

Moreover, a Cochrane review of 140 studies concluded that the audit could lead to improvements in professional practice, but this depends on how well facilities perform at baseline and how the feedback is provided [25].

Although perinatal death audit can be effective in improving quality of care, there are some reports where this did not improve perinatal outcomes. In a five-year study of health facilities in South Africa, it was observed that among the 54 health facilities that started perinatal death audit, only 30% had a decrease in perinatal mortality, while 35% had an increase [26]. The study identified several facility-related factors that contributed to the increase in perinatal mortality, including lack of use of antenatal steroids, lack of nursing personnel, fetal distress not detected during the antepartum period and incorrect use of the partograph.

Additional studies, ideally randomised controlled trials using a stepped wedge design, are needed to assess the effect of perinatal death audit on perinatal outcomes.

CONCLUSION

This study highlights the potential of perinatal death review to identify how care pathways can be improved over time to reduce adverse perinatal outcomes in low-resource settings. This study shows that the introduction of perinatal death allowed identifying the main causes of death and contributing factors, and prioritise areas in the provision of healtchre that need improvement. The findings of the study should encourage more healthcare providers to undertake perinatal death reviews, especially in low-resource settings where the majority of global perinatal mortality occurs [27,28].

For peer review only - http://bmjopen.bmj.com/site/about/guidelines.xhtml

DECLARATIONS

Ethical approval and consent to publish

Approval for the study was obtained from the Nsambya Hospital Ethical Review Committee. Individual patient consent was not applicable as the research team extracted anonymized data from case notes.

Patient and public involvement

No patient involved.

Acknowledgements

We would like to thank all healthcare providers who took part in the review process. We particularly appreciate the contributions of Dr. Musana Othniel, Dr. Sebunya Robert, Dr. Nazziwa Rita, Dr. Catherine Nyagabyaki, Sr. Nabwami Immaculate, Dr. Tumwine Gilbert and Dr. Zaake.

We are also grateful to Robert Lloyd for his contribution to data processing, to Prof. Mathews Mathai for reviewing the manuscript and to Caroline Hercod for editing the manuscript.

Disclosure of Interests

All authors declare no competing interests.

Funding

No funding was received for the data collection. However, the cost of data processing, analysis and publication was sponsored by the 'Making it Happen' Programme funded by DFID/UKAid.

Availability of data and materials

The datasets used and/or analysed during the current study are available from the corresponding author upon reasonable request.

Contributions to Authorship

For peer review only - http://bmjopen.bmj.com/site/about/guidelines.xhtml

For peer review only

VN contributed to study design, data collection, analysis and writing up. MA processed and analyzed the data and contributed to all drafts of the paper. JED contributed to the conception of the paper, to the analysis, and writing of the paper. RB took part in writing the study proposal, data collection and review of cases. PO contributed to the study proposal, data collection and case reviews. NVDB oversaw the conception and analysis and contributed to the initial and final drafts of the paper.

REFERENCES

1. World Health Organization. WHO Fact Sheet Stillbirths. World Health Organization: Geneva, 2015.
2. Uganda Ministry of Health, Reproductive Health Division. Annual Maternal and Perinatal Death Review Report 2013/2014. Ministry of Health: Kampala, 2014.
3. Uganda Bureau of Statistics. Uganda Demographic and Health Survey 2011. Uganda Bureau of Statistics: Kampala 2011. https://dhsprogram.com/pubs/pdf/fr264/fr264.pdf
4. Buchmann EJ. Towards greater effectiveness of perinatal death audit in low- and middle-income countries. *BJOG*. 2014;**121**(Supp 4):S134-6. doi: 10.1111/1471-0528.12904
5. Eskes M, Waelput AJM, Erwich JJHM, *et al*. Term perinatal mortality audit in the Netherlands 2010–2012: a population based cohort study. *BMJ Open*. 2014;**4**:e005652. doi: 10.1136/bmjopen-2014-005652
6. Pattinson R, Keber K, Waiswa P, *et al*. Perinatal Mortality Audit: Counting, accountability, and overcoming challenges in scaling up in low- and middle-income countries. *Int J Gynaecol Obstet*. 2009;**109**(Supp 1):113-22. doi: 10.1016/j.ijgo.2009.07.011.
7. World Health Organization. ICD-10 International statistical classification of diseases and related health problems. World Health Organization: Geneva, 2004.
8. Bernal JL, Cummins S, Gasparrini A. Interrupted time series regression for the evaluation of public health interventions: a tutorial. *Int J Epidemiol.* 2017;**46**:348-55 doi: 10.1093/ije/dyw098
9. Kidanto HL, Mogren I, van Roosmalen J, *et al*. Introduction of a qualitative perinatal audit at Muhimbili National Hospital, Dar es Salaam, Tanzania. *BMC Pregn Childb*. 2009;**9**:45. doi: 10.1186/1471-2393-9-45
10. Kidanto HL, Massawe SN, Nyström L, *et al*. Analysis of perinatal mortality at a teaching hospital in Dar es Salaam, Tanzania. *Afr J Reprod Health*. 2006;**10**:72-80.
11. Boo NY. Neonatal resuscitation programme in Malaysia: an eight-year experience. *Singapore Med J*. 2009;**50**:152-9.
12. Hole MK, Olmsted K, Kiromera A, *et al*. Neonatal Resuscitation Curriculum in Malawi, Africa: Did It Change In-Hospital Mortality? *Int J Pediatr*. 2012;**2012**:408689. doi: 10.1155/2012/408689
13. Conde-Agudelo A, Belizán JM, Diaz-Rossello J. Kangaroo mother care to reduce morbidity and mortality in low birthweight infants. *Cochrane Database Syst Rev*. 2011;**3**:CD002771. doi: 10.1002/14651858.CD002771.pub2.
14. Mwansa-Kambafwile J, Cousens S, Hansen T, *et al*. Antenatal steroids in preterm labour for the prevention of neonatal deaths due to complications of preterm birth. *Int J Epidemiol*. 2010;**39**(Supp 1):i122–33. doi: 10.1093/ije/dyq029.

For peer review only - http://bmjopen.bmj.com/site/about/guidelines.xhtml

15. Ho JJ, Subramaniam P, Henderson-Smart DJ, *et al*. Continuous distending pressure for respiratory distress syndrome in preterm infants. *Cochrane Database Syst Rev*. 2002;**2**:CD002271.
16. Soll R, Özek E. Prophylactic animal derived surfactant extract for preventing morbidity and mortality in preterm infants. *Cochrane Database Syst Rev*. 2002;**4**:CD000511.
17. Aminu M, Unkels R, Mdegela M, *et al*. Causes of and factors associated with stillbirth in low- and middle-income countries: a systematic literature review. *BJOG*. 2014;**121**(Supp 4):141-53. doi: 10.1111/1471-0528.12995.
18. Lawn J, Shibuya K, Stein C. No cry at birth: Global estimates of intrapartum stillbirths and intrapartum-related neonatal deaths. *Bull World Health Organ*. 2005;**83**:409-17.
19. Lee ACC, Cousens S, Wall SN, *et al*. Neonatal resuscitation and immediate newborn assessment and stimulation for the prevention of neonatal deaths. *BMC Public Health*. 2011;**11**(Supp 3):S12. doi: 10.1186/1471-2458-11-S3-S12
20. Genest DR, Singer DB. Estimating the time of death in stillborn fetuses: III. External fetal examination; a study of 86 stillborns. *Obstet Gynecol*. 1992;**80**:593-600.
21. Gold KJ, Abdul-Mumin AS, Boggs ME, *et al*. Assessment of "fresh" versus "macerated" as accurate markers of time since intrauterine fetal demise in low income countries. *Int J Gynaecol Obstet*. 2014;**125**:223-7. doi: 10.1016/j.ijgo.2013.12.006
22. Kawaza K, Machen HE, Brown J, *et al*. Efficacy of a low-cost bubble CPAP system in treatment of respiratory distress in a neonatal ward in Malawi. *PLoS One*. 2014;**9**:e86327. doi: 10.1371/journal.pone.0086327
23. Bhutta ZA, Das JK, Bahl R, *et al*. Can available interventions end preventable deaths in mothers, newborn babies, and stillbirths, and at what cost? *Lancet*. 2014;**384**:347-70. doi: 10.1016/S0140-6736(14)60792-3
24. Oza S, Lawn LE, Hogan DR, *et al*. Neonatal cause-of-death estimates for the early and late neonatal periods for 194 countries: 2000-2013. *Bull World Health Organ*. 2015;**93**:19-28. doi: 10.2471/BLT.14.139790
25. Ivers N, Jamtvedt G, Flottorp S, *et al*. Audit and feedback: effects on professional practice and healthcare outcomes. *Cochrane Database Syst Rev*. 2012;**6**:CD000259. doi: 10.1002/14651858.CD000259.pub3
26. Allanson ER, Pattinson RC. Quality-of-care audits and perinatal mortality in South Africa. *Bull World Health Organ*. 2015;**93**:424-8. doi: 10.2471/BLT.14.144683
27. Lawn JE, Blencowe H, Pattinson R, *et al*. Stillbirths: Where? When? Why? How to make the data count? *Lancet*. 2011;**377**:1448-63. doi: 10.1016/S0140-6736(10)62187-3
28. Lawn JE, Blencowe H, Waiswa P, *et al*. Stillbirths: rates, risk factors, and acceleration towards 2030. *Lancet*. 2016;**387**:587-603. doi: 10.1016/S0140-6736(15)00837-5.

For peer review only

For peer review only - http://bmjopen.bmj.com/site/about/guidelines.xhtml

Figure and Table Captions

Figure 1: Cause of death for macerated stillbirths (n=142), fresh stillbirths (n=125) and early neonatal deaths (n=261)

Figure 2: Interrupted time series without overdispersion

Figure 3: Interrupted time series with overdispersion

Table 1: Demographic and clinical characteristics

Table 2: Interrupted time series analysis for selected perinatal outcomes

Supplementary Table 1: Stillbirth rate (per 1000 births), early neonatal mortality rate (per 1000 live births) and perinatal mortality rate (per 1000 births)

Supplementary Table 2: Cause of death for stillbirth and early neonatal death

Supplementary Table 3: Case fatality rates (CFR)

Supplementary Table 4: Standard of care

For peer review only

Table 1: Demographic and clinical characteristics

Characteristics	Fresh stillbirth n=125 (%)	Macerated stillbirth n=142 (%)	Early neonatal death n=259 (%)	Total n=526 (%)
**Referral**				
From other healthcare facility	32 (25.6)	15 (10.6)	37 (14.3)	84 (16.0)
From Traditional Birth Attendant	1 (0.8)	1 (0.7)	1 (0.4)	3 (0.6)
**Parity**				
1	35 (28.0)	48 (33.8)	129 (49.8)	212 (40.3)
2 to 4	64 (51.2)	67 (47.2)	84 (32.4)	215 (40.9)
5 or more	10 (8.0)	15 (10.6)	18 (6.9)	43 (8.2)
**Antenatal care**				
At least 1 ANC visit	107 (85.6)	129 (90.8)	234 (90.3)	470 (89.4)
4 or more ANC visits	38 (30.4)	47 (33.1)	98 (37.8)	183 (34.8)
**Type of pregnancy**				
Single pregnancy	113 (90.4)	131 (92.3)	227 (87.6)	471 (89.5)
Multiple pregnancy	2 (1.6)	5 (3.5)	14 (5.4)	21 (4.0)
**Mode of delivery**				
Spontaneous vaginal delivery	67 (53.6)	123 (86.6)	143 (55.2)	333 (63.3)
Assisted breech delivery	6 (4.8)	2 (1.4)	10 (3.9)	18 (3.4)
Instrumental delivery	4 (3.2)	1 (0.7)	3 (1.2)	8 (1.5)
Caesarean section	45 (36.0)	10 (7.0)	92 (35.5)	147 (27.9)
**Gestational age at delivery**				
<37 weeks	29 (23.2)	56 (39.4)	74 (28.6)	159 (30.2)
37-41 weeks	60 (48.0)	57 (40.1)	132 (51.0)	249 (47.3)
>41 weeks	5 (4.0)	4 (2.8)	9 (3.5)	18 (3.4)
**Baby's sex**				
Male	68 (54.4)	80 (56.3)	146 (56.4)	294 (55.9)
Female	52 (41.6)	57 (40.1)	99 (38.2)	208 (39.5)
**Birthweight**				
<1000g	1 (0.8)	16 (11.3)	9 (3.5)	26 (4.9)
1000-2499g	30 (24.0)	52 (36.6)	81 (31.3)	163 (31.0)
2500-4000g	77 (61.6)	61 (43.0)	149 (57.5)	287 (54.6)
>4000g	3 (2.4)	7 (4.9)	6 (2.3)	16 (3.0)

For peer review only

For peer review only - http://bmjopen.bmj.com/site/about/guidelines.xhtml

Table 2: Interrupted time series analysis for selected perinatal outcomes

	Without over-dispersion				With over-dispersion			
	Intervention		Time		Intervention		Time	
**Outcome**	**IRR (95% CI)**	**p-value**	**IRR (95% CI)**	**p-value**	**IRR (95% CI)**	**p-value**	**IRR (95% CI)**	**p-value**
Perinatal death rate	1.17 (1.02, 1.34)	**0.021**	0.94 (0.92,0.96)	**<0.001**	1.17 (0.79, 1.74)	0.44	0.94 (0.89,1.00)	0.06
Stillbirth rate	1.31 (1.14,1.51)	**<0.001**	0.93 (0.91,0.95)	**<0.001**	1.31 (0.84,2.06)	0.23	0.93 (0.87,1.00)	**0.039**
Early neonatal death rate	0.35 (0.22,0.55)	**<0.001**	1.09 (1.01,1.17)	**0.01**	0.35 (0.22,0.56)	**<0.001**	1.09 (1.01,1.17)	**0.01**
CFR for intrapartum-related hypoxia	0.77 (0.56,1.06)	0.11	0.98 (0.93,1.03)	0.42	0.77 (0.57,1.04)	0.09	0.98 (0.93,1.03)	0.40
CFR for prematurity	1.01 (0.73,1.41)	0.95	0.94 (0.90,0.98)	**0.004**	1.01 (0.64,1.59)	0.96	0.94 (0.88,0.99)	**0.04**
CFR for infections	2.25 (0.38,13.3)	0.37	1.047 (0.86,1.27)	0.68	2.25 (0.17,28.9)	0.54	1.04 (0.78,1.39)	0.77

IRR: Incidence risk ratio

For peer review only

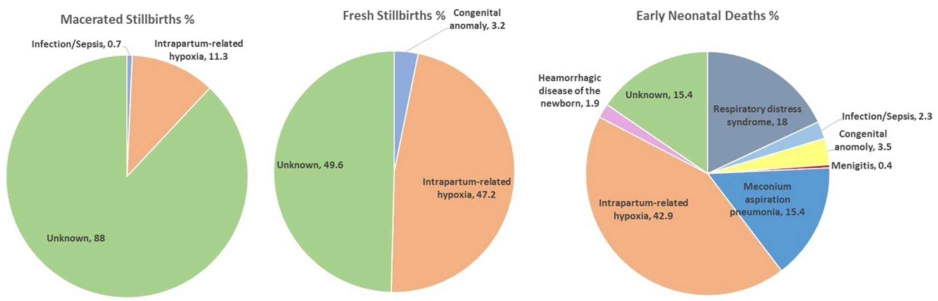

Figure 1: Cause of death for macerated stillbirths (n=142), fresh stillbirths (n=125) and early neonatal deaths (n=261)

338x190mm (96 x 96 DPI)

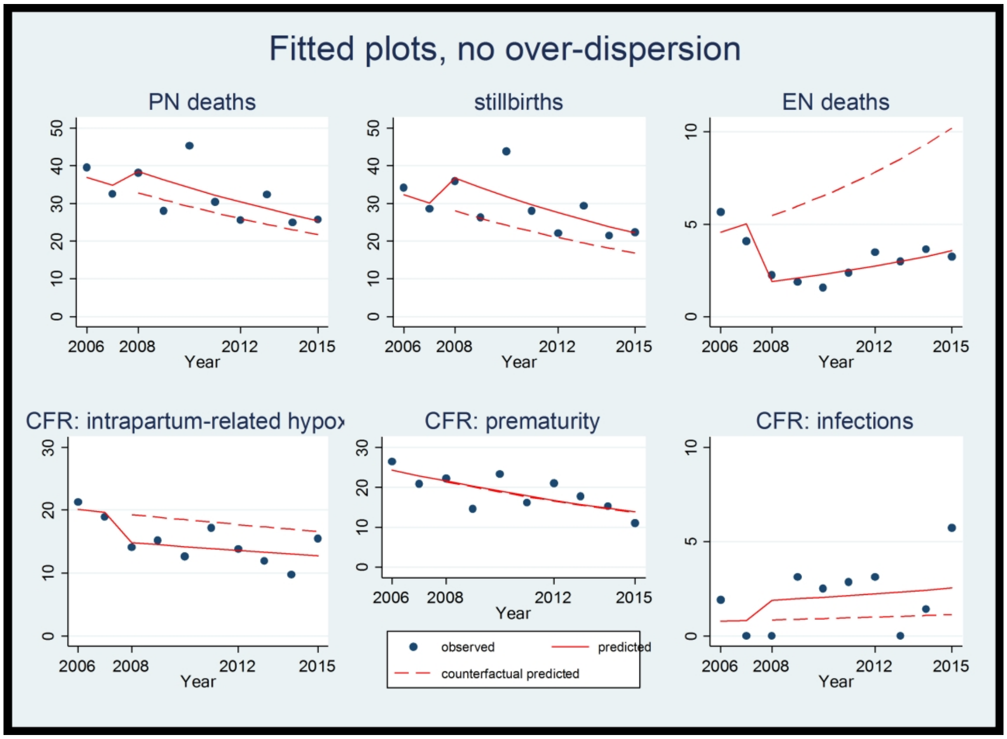

Figure 2: Interrupted time series without overdispersion

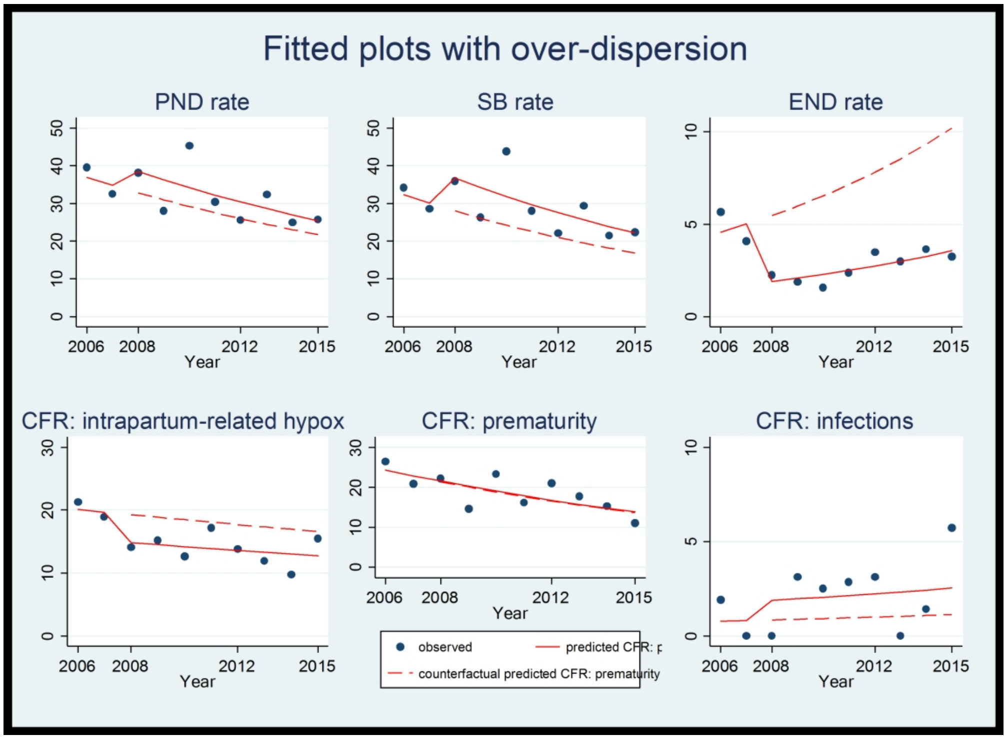

Figure 3: Interrupted time series with overdispersion

Supplementary Materials

Supplementary Table 1: Stillbirth rate (per 1000 births), early neonatal mortality rate (per 1000 live births) and perinatal mortality rate (per 1000 births).

Year	Total Births	Live Births	Stillbirth	Stillbirth Rate	Early NND	Early NND Rate	Perinatal Mortality	Perinatal Mortality Rate
2006	6468	6020	213	32.9	96	16	309	47.8
2007	7290	6881	210	28.8	193	28.1	403	55.3
2008	8386	8408	291	34.7	145	17.3	436	52.0
2009	8452	7995	207	24.5	114	14.3	321	38.0
2010	9513	8877	229	24.1	132	14.9	362	38.0
2011	8050	7610	220	27.3	166	21.8	386	47.9
2012	6732	6593	149	22.1	167	25.4	316	47.0
2013	6376	6011	183	28.7	117	19.4	300	47.0
2014	6026	5764	136	22.6	110	19	246	40.8
2015	5462	5222	120	22	112	21.5	232	42.6

For peer review only

Supplementary Table 2: Cause of death for stillbirth and early neonatal death.

Cause of Death	Macerated Stillbirth n=142 (%)	Fresh Stillbirth n=124 (%)	Early Neonatal Death n=259 (%)	TOTAL n=526 (%)
Respiratory distress syndrome	-	-	47 (18.0)	**47 (8.9)**
Infections / Septicaemia	1 (0.7)	0	6 (2.3)	**7 (1.3)**
Congenital anomaly	0	4 (3.2)	9 (3.5)	**13 (2.5)**
Meningitis	-	-	1 (0.4)	**1 (0.2)**
Meconium aspiration pneumonia	-	-	40 (15.4)	**40 (7.6)**
Intrapartum-related hypoxia	16 (11.3)	59 (47.6)	111 (42.9)	**186 (35.4)**
Haemorrhagic disease of newborn	-	-	5 (1.9)	**5 (1.0)**
Unknown	125 (88.0)	61 (49.6)	40 (15.4)	**227 (43.2)**

For peer review only

Supplementary Table 3: Case fatality rates (CFR)

Cause of death		2006	2007	2008	2009	2010	2011	2012	2013	2014	2015
**Intrapartum-related hypoxia**	Intrapartum-related hypoxia admissions	202	291	334	302	316	297	247	252	195	187
	Intrapartum-related hypoxia deaths	43	55	47	46	40	51	34	30	19	29
	**Intrapartum-related hypoxia CFR (%)**	**21.3**	**18.9**	**14.1**	**15.2**	**12.7**	**17.2**	**13.8**	**11.9**	**9.7**	**15.5**
**Prematurity**	Prematurity admissions	163	168	194	246	210	328	286	304	336	371
	Premature deaths	43	35	43	36	49	53	60	54	51	41
	**Prematurity CFR (%)**	**26.4**	**20.8**	**22.2**	**14.6**	**23.3**	**16.2**	**21.0**	**17.8**	**15.2**	**11.1**
**Infections**	Number of admissions for infections	104	149	82	128	120	140	129	171	141	87
	Deaths due to infections	2	0	0	4	3	4	4	0	2	5
	**Infections CFR (%)**	**1.9**	**0**	**0**	**3.1**	**2.5**	**2.9**	**3.1**	**0.0**	**1.4**	**5.7**

For peer review only

Supplementary Table 4: Standard of care

**Standard of care**	**MSB (%)**	**FSB (%)**	**ENND (%)**	**Total (%)**
Optimal	82 (67.8)	58 (49.2)	112 (45.7)	262 (53.0)
Probably acceptable	21 (17.4)	28 (23.7)	58 (23.7)	107 (21.7)
Probably sub-optimal	12 (9.9)	24 (20.3)	46 (18.8)	82 (16.6)
Sub-optimal	6 (5.0)	8 (6.8)	29 (11.8)	43 (8.7)
**Total**	**121 (100)**	**118 (100)**	**245 (100)**	**494 (100)**

For peer review only

BMJ Open

Prospective Study to Explore Changes in Quality of Care and Perinatal Outcomes After Implementation of Perinatal Death Audit in Uganda

Journal:	*BMJ Open*
Manuscript ID	bmjopen-2018-027504.R2
Article Type:	Research
Date Submitted by the Author:	12-Sep-2019
Complete List of Authors:	Kirabira, Victoria; Nsambya Hospital<br>Aminu, Mamuda; Liverpool School of Tropical Medicine, Centre for Maternal and Newborn Health ; Liverpool School of Tropical Medicine<br>Dewez, Juan; Liverpool School of Tropical Medicine, Centre for Maternal and Newborn Health<br>Byaruhanga, Romano; Nsambya Hospital<br>Okong, Pius; Nsambya Hospital<br>van den Broek, Nynke; Liverpool School of Tropical Medicine, Centre for Maternal and Newborn Health
**Primary Subject Heading**:	Obstetrics and gynaecology
Secondary Subject Heading:	Global health, Paediatrics, Public health, Reproductive medicine
Keywords:	AUDIT, Reproductive medicine < GYNAECOLOGY, Clinical audit < HEALTH SERVICES ADMINISTRATION & MANAGEMENT, Quality in health care < HEALTH SERVICES ADMINISTRATION & MANAGEMENT, stillbirth, neonatal death

SCHOLARONE™
Manuscripts

I, the Submitting Author has the right to grant and does grant on behalf of all authors of the Work (as defined in the below author licence), an exclusive licence and/or a non-exclusive licence for contributions from authors who are: i) UK Crown employees; ii) where BMJ has agreed a CC-BY licence shall apply, and/or iii) in accordance with the terms applicable for US Federal Government officers or employees acting as part of their official duties; on a worldwide, perpetual, irrevocable, royalty-free basis to BMJ Publishing Group Ltd ("BMJ") its licensees and where the relevant Journal is co-owned by BMJ to the co-owners of the Journal, to publish the Work in this journal and any other BMJ products and to exploit all rights, as set out in our licence.

The Submitting Author accepts and understands that any supply made under these terms is made by BMJ to the Submitting Author unless you are acting as an employee on behalf of your employer or a postgraduate student of an affiliated institution which is paying any applicable article publishing charge ("APC") for Open Access articles. Where the Submitting Author wishes to make the Work available on an Open Access basis (and intends to pay the relevant APC), the terms of reuse of such Open Access shall be governed by a Creative Commons licence – details of these licences and which Creative Commons licence will apply to this Work are set out in our licence referred to above.

Other than as permitted in any relevant BMJ Author's Self Archiving Policies, I confirm this Work has not been accepted for publication elsewhere, is not being considered for publication elsewhere and does not duplicate material already published. I confirm all authors consent to publication of this Work and authorise the granting of this licence.

For peer review only

Prospective Study to Explore Changes in Quality of Care and Perinatal Outcomes After Implementation of Perinatal Death Audit in Uganda

----

Victoria Nakibuuka Kirabira[a], Mamuda Aminu[b§], Juan Emmanuel Dewez[b], Romano Byaruhanga[a], Pius Okong[a], Nynke van den Broek[b]

[a] Nsambya Hospital, P.O. Box 7146, Kampala, Uganda

[b] Centre for Maternal and Newborn Health, Liverpool School of Tropical Medicine, Pembroke Place, Liverpool L3 5QA, UK

[§]Corresponding Author Contact Details:

Dr Mamuda Aminu
Centre for Maternal and Newborn Health,
Liverpool School of Tropical Medicine, Pembroke Place, Liverpool L3 5QA.
Tel: +44(0)151 705 3342
Email: Mamuda.Aminu@lstmed.ac.uk

Author email addresses:

VN Kirabira	nakibuukarv@yahoo.com
JE Dewez	manuel.dewez@lshtm.ac.uk
R Byaruhanga	byaruhangarn@yahoo.com
P Okong	puisokong@yahoo.com
NR van den Broek	Nynke.Broek@lstmed.ac.uk

Running Title: Perinatal death audit in Uganda

Keywords: perinatal death, cause of perinatal death, quality improvement

***

Word Count (Body Only): 3680

Abstract: 300

For peer review only - http://bmjopen.bmj.com/site/about/guidelines.xhtml

ABSTRACT

Objective: To assess the effects of perinatal death audit on perinatal outcomes in a tertiary hospital in Kampala

Design: Interrupted time series (ITS) analysis

Setting: Nsambya Hospital, Uganda

Participants: Live births and stillbirths

Interventions: Perinatal death audit

Primary and secondary outcome measures: Primary outcomes: perinatal mortality rate, stillbirth rate, early neonatal mortality rate. Secondary outcomes: case fatality rates (CFR) for asphyxia, complications of prematurity and neonatal sepsis.

Results

526 perinatal deaths were audited: 142 (27.0%) fresh stillbirths, 125 (23.8%) macerated stillbirths and 259 (49.2%) early neonatal deaths.

The ITS analysis showed a decrease in perinatal death (PND) rates without the introduction of perinatal death audits (IRR (95% CI) for time=0.94, p<0.001), but an increase in PND (IRR (95% CI) =1.17 (1.0,-1.34), p=0.0021) following the intervention. However, when over-dispersion was included in the model, there were no statistically significant differences in PND with or without the intervention (p=0.06 and p=0.44 respectively).

Stillbirth rates exhibited a similar pattern. By contrast, early neonatal death rates showed an overall upward trend without the intervention (IRR (95% CI)= 1.09 (1.01-1.17), p=0.01), but a decrease following the introduction of the perinatal death audits (IRR (95% CI)= 0.35 (0.22-0.56), p<0.001), when over-dispersion was included.

The CFR for prematurity showed a downward trend over time (IRR (95% CI) =0.94 (0.88-0.99), p=0.04) but not for the intervention. With regards CFRs for intrapartum-related hypoxia or infection, no statistically significant effect was detected for either time or the intervention.

Conclusion

The introduction of perinatal death audit showed no statistically significant effect on perinatal mortality or stillbirth rate, but a significant decrease in early neonatal mortality rate. No effect was detected on case fatality rates for prematurity, intrapartum-related hypoxia or infections. These findings should encourage more healthcare workers to undertake research to assess the effectiveness of perinatal death reviews in low-resource settings.

Strengths and limitations of this study

For peer review only - http://bmjopen.bmj.com/site/about/guidelines.xhtml

- This study is one of the few studies assessing the effect of perinatal death audits in a resource-limited setting.
- The interrupted time series design was useful to assess the effect of the intervention, but additional studies, such as stepped-wedge randomised controlled trials, must confirm the findings of this study.
- The inherent limitation of the methods used to assess the cause of death was the dependence on hospital records, which were often incomplete, inaccurate or both.
- Only two timepoints of data were documented before the introduction of the audit, which could have influenced the effect on outcomes of interest.

For peer review only

For peer review only - http://bmjopen.bmj.com/site/about/guidelines.xhtml

BACKGROUND

Currently, about 2.7 million neonatal deaths and 2.6 million stillbirths occur worldwide every year. Of these, the majority occur in low- and middle-income countries, and more than half (55%) of the global stillbirths occur in sub-Saharan Africa and South Asia [1]. Uganda's perinatal mortality is estimated to be 36 deaths per 1000 births and remains unacceptably high. Almost 50% of these are fresh stillbirths [2]. The neonatal mortality rate (21 per 1000 live births) in Uganda is also high, contributing 50% of the infant mortality rate [3]. It is crucial that interventions are put in place to reduce preventable neonatal deaths and stillbirths.

Perinatal death audit (or review) can be used to identify areas of care which can be strengthened to improve perinatal outcomes [4]. It involves reviewing cases of mortality with a view to identifying avoidable factors and implementing changes to prevent future deaths. A population-based cohort study of 943 perinatal mortalities that occurred in 90 hospitals in the Netherlands reported a decrease in substandard care (from 10% to 5%) and perinatal mortality (from 2.3 to 2.0/1000 births; $p<0.00001$) after the introduction of perinatal death audit [5].

Similarly, in a systematic review focusing on low- and middle-income countries, a meta-analysis of 7 before-and-after studies reported a 30% (95% confidence interval, 21%–38%) reduction in perinatal mortality after introduction of perinatal death audit in healthcare facilities [6]. There is, however, a paucity of data from low-resource settings on the use of and effectiveness of perinatal death audit to improve the quality of care provided to mothers and their babies. The systematic review recommended more research in key areas, including exploring the effect of facility-based perinatal death audit on changing the quality of care [6].

The aim of thisstudy is to assess the changes in perinatal outcomes observed after the introduction of perinatal death audit in a tertiary hospital in Uganda and to describe the changes in healthcare provided to mother and babies resulting from the perinatal death audits.

METHODS

Study setting

For peer review only - http://bmjopen.bmj.com/site/about/guidelines.xhtml

This study was conducted at Nsambya Hospital, a private not-for-profit tertiary hospital in Kampala, Uganda, which serves as a referral centre for Makindye Health Sub-district in Kampala. The facility receives 7,000 deliveries per year and provides 24-hour comprehensive emergency obstetric and newborn care services, with a staff of 6 obstetricians, 18 resident doctors, 5 intern doctors and 40 midwives.

The newborn care unit admits 2,500 babies annually and has a paediatrician/neonatologist, two residents and up to four midwives on duty per shift. It has 40 beds, including 10 neonatal intensive beds, and provides essential newborn care including; oxygen therapy, surfactant and low-cost bubble continuous positive airway pressure (CPAP), kangaroo mother care and phototherapy.

Data collection

Perinatal death audit using locally developed tools and guidelines started in 2008 and was led by a multidisciplinary audit team comprising of obstetricians, paediatricians, resident doctors, midwives and administrators. Team meetings were held weekly, chaired by the most senior obstetrician or paediatrician. Perinatal death was defined as "death of a fetus greater than a birth weight of 1000g if measured, or a gestational age of at least 28 completed weeks or more, or a body length (crown-heel) of at least 35cm or more"[7] and included all stillbirths and deaths within the first week of life (early neonatal deaths). The number of perinatal deaths was documented daily using clinical handover reports and registers in the labour ward and neonatal special care unit. Due to the large numbers of deaths, one-quarter of the cases (every 4th case) were selected for audit from each of the following categories: fresh stillbirths, macerated stillbirths and early neonatal deaths.

The number of admissions in the newborn unit, reasons for admission, death and case fatalities rates for intrapartum-related hypoxia, infection and prematurity were collected for the period before the perinatal audit was introduced (2006-2007) and subsequently for the period after commencement of the perinatal death audit (2008-2015). The data was obtained and aggregated monthly through a retrospective analysis of registers and patient records. Numbers of live and total births were also documented to enable rates calculation.

Intervention: the audit process

For each case selected for review, an audit form was completed by a resident doctor, who also provided a narrative summary for each case. Sources of information included: clinical handover books, maternity

For peer review only - http://bmjopen.bmj.com/site/about/guidelines.xhtml

registers, newborn admission register, and clinical notes for mothers and newborns. Each case was then presented to the rest of the audit team for detailed discussion. The information presented to the team allowed reviewing the cause of death and assigning a more precise cause of death by consensus. C are provided was judged against existing local protocols and national guidelines. No clinical autopsies were performed due to lack of a full-time pathologist at the hospital.

The quality of care provided at the hospital was also graded by consensus and classified into four categories:

- Optimal care generally accepted practice and all standards of care were followed.
- Suboptimal care but different management would have made no difference to the outcome (probably acceptable)
- Suboptimal care where different management might have made a difference to the outcome - an avoidable factor of uncertain clarity or influence on the outcome (probably suboptimal)
- Different management would reasonably be expected to have made a difference to the outcome. A clearly avoidable factor implying that the adverse outcome could have been prevented) (suboptimal)

The overall grading of the care provided to the mother and baby, the identified specific areas of suboptimal care and the recommendations for improvement were documented. This grading included referred mothers as well, whereas there were delays from the referring site, suboptimal care can also occur in tertiary hospital as well, such as delay in performing a Caesarean section

The summary of each case included a list of the actions to be taken. In each case, a specific person was appointed to lead the implementation of the relevant actions. During monthly meetings, implementation progress was compared with the action plan by the wider audit team. A number of interventions, such as neonatal resuscitation training, the introduction of Kangaroo Mother Care (KMC), building of a new maternity theatre, were thus introduced gradually over the years to address the gaps in care.

Data analysis

SPSS© (IBM, version 22) was used for descriptive analyses. Stillbirth and perinatal mortality rates were expressed per 1000 total births, while early neonatal mortality rate was expressed per 1000 live births. Case fatality rate (CFR) was defined as the proportion of deaths among cases diagnosed with the disease. Statistical significance was calculated at 95% confidence interval or p-value of <0.05.

For peer review only - http://bmjopen.bmj.com/site/about/guidelines.xhtml

Data for the six primary and secondary outcomes were each analysed using interrupted time series (ITS) models in Stata©. Poisson regression models of counts were fitted with covariates for intervention and year, and the relevant population size (log-transformed) used as the offset. As proposed by Bernal et al [8] Over-dispersion was considered (by using an additional option: scale(x2)) since it often arises in the context of count data. Auto-correlation was also inspected using plots. The 5% level was used to determine statistical significance.

Since low birth weight and prematurity are both associated with a higher risk of perinatal mortality, a one-way between-groups analysis of variance (ANOVA) was conducted to explore changes in birth weight and gestational age over time.

A narrative summary description was used to report actions agreed and implemented following an audit. These were documented chronologically as derived from minutes of the audit meetings.

Ethical approval

Approval for the study was obtained from the Nsambya Hospital Ethical Review Committee.

Patient and public involvement

Individual patient consent was not applicable as the research team extracted anonymized data from case notes. Patients and the public were not involved in this study.

RESULTS

Demographic and clinical characteristics:

A total of 58,997 births and 2,616 perinatal deaths occurred between 2008 and 2015 (Supplementary Table 1). Of these, 603 (23%) perinatal deaths were selected for audit. After review, 526 were found to meet the definition of perinatal mortality: 142 (27.0%) fresh stillbirths, 125 (23.8%) macerated stillbirths and 259 (49.2%) early neonatal deaths (Table 1).

The majority of cases came to the hospital directly, with 17% referrals. Mothers had a mean age of 27.3 years (SD: 5.1); median parity of 2 (SD of 1.6). Just over one-third of fresh stillbirths and early neonatal

For peer review only - http://bmjopen.bmj.com/site/about/guidelines.xhtml

deaths were delivered by Caesarean section. The mean birth weight of babies was 2610 grams (SD: 969.4). Between 23.2% and 39.4% were born prematurely (Table 1).

Cause of death:

Overall, intrapartum-related hypoxia was the leading cause of perinatal mortality in this setting, accounting for 35.4% of all cases (Figure 1 and Supplementary Table 2). This is followed by respiratory distress syndrome (8.9%) and meconium aspiration pneumonia (7.6%). Cause of death in 43.2% could not be ascertained: 15.4% for neonatal deaths, 49.2% for fresh stillbirths and 88.0% for macerated stillbirths. However, the proportion of deaths for which cause of death could not be established ("cause unknown") decreased from 34.1% (overall) in 2008 to 2.7% in 2013.

Trend in perinatal mortality rate:

The ITS analysis showed that, without modelling over-dispersion, there would have been a downward trend (a decrease) in perinatal death rates (**Incidence risk ratio** (IRR) (95% CI) for time=0.94, p<0.001), while a statistically significant increase (IRR (95% CI) =1.17 (1.0,-1.34), p=0.0021) was observed following the introduction of perinatal death audits. However, when over-dispersion was included in the model, both effects were not statistically significant (Table 2, Figure 2, and Supplementary Table 3).

Stillbirth rates exhibited a similar pattern. By contrast, early neonatal death rates showed an overall upward trend (IRR (95% CI)= 1.09 (1.01-1.17), p=0.01) but a a decrease with the introduction of the perinatal death audits (IRR (95% CI) = 0.35 (0.22-0.56), p<0.001) when over-dispersion was included in the model (Table 2, and Figures 2). The ANOVA to explore changes in birth weight and gestational age over time showed no statistically significant change in birthweight over time (p = 0.872), and no change in gestational age at birth over time (p = 0.219).

The CFR for prematurity showed a downward trend over time (IRR (95% CI)=0.94 (0.88-0.99), p=0.04) but a downward trend was not observed with the intervention. With regards the CFRs for intrapartum-related hypoxia and infections, no statistically significant effect was detected with or without the intervention (Table 2, Figures 2, and Supplementary Table 3).

For each outcome considered, the autocorrelation and partial correlation plots provided no evidence that there was autocorrelation not accounted for in the models used.

For peer review only - http://bmjopen.bmj.com/site/about/guidelines.xhtml

Quality improvement:

More than half of the cases (53%) were deemed to have had optimal care. The rest had probably acceptable care (21.7%), probably sub-optimal care (16.6%) or sub-optimal care (8.7%) (Supplementary Table 4). Among the different types of mortalities, sub-optimal care was most common among early neonatal deaths (11.8%), as compared to fresh stillbirth (6.8%) and macerated stillbirth (5.0%).

DISCUSSION

Main findings

This study assessed the effect of perinatal death audit to improve the quality of perinatal care and perinatal outcomes in a tertiary hospital in Uganda over a period of 10 years.

Intrapartum-related hypoxia was the leading cause of perinatal mortality (accounting for 35.4%), followed by respiratory distress syndrome (8.9%) and meconium aspiration pneumonia (7.6%). Cause of death was unknown in 43.2% of cases. The proportion of deaths with unknown cause decreased from 34.1% (overall) in 2008 to 2.7% in 2013.

There was no effect of perinatal death audit on perinatal mortality or stillbirth rates. However, the intervention led to a decrease in neonatal mortality rate [IRR (95% CI)= 0.35 (0.22-0.56), p<0.001)]. It was noted that the high numbers of stillbirth inflated the numbers of perinatal death. Thus, even though the audit made a difference to early neonatal deaths, this difference is neutralised when combined with stillbirths.

The CFR for prematurity showed a downward trend over time (IRR (95% CI)=0.94 (0.88-0.99), p=0.04), but this was not related to the perinatal death audits. The CFRs for intrapartum-related hypoxia and infections were not affected by the intervention.

More than half of the cases (53%) were deemed to have had optimal care, with the rest of the cases having varying levels of acceptability. Early neonatal deaths had the highest proportion of cases with suboptimal care (11.8%). Case fatality rates decreased between 2006 and 2015 for intrapartum-related hypoxia (21.3% to 15.5%) and for complications of prematurity (26.4% to 11.1%). However, case fatality rates for infection increased from 1.9% in 2006 to 5.7% in 2015.

Quality improvement

For peer review only - http://bmjopen.bmj.com/site/about/guidelines.xhtml

In 2007 and 2008, the perinatal death review team observed many cases of delay between the decision for, and the actual conduct of, Caesarean Section, which was associated with high numbers of intrapartum-related hypoxia. To reduce the time taken to prepare mothers for the procedure, the team recommended the involvement of doctors in the preparation. These ensured mothers were catheterized and blood samples were taken for grouping and cross-matching at the time of decision. During the same period, a new dedicated operating theatre was built close to the maternity ward.

In 2008, two major factors contributing to perinatal mortality were identified to have recurred consistently: poor neonatal resuscitation and poor use of the partographs. Both of these were thought to contribute to perinatal death due to intrapartum-related hypoxia. To tackle the problem of poor resuscitation skills, maternity staff were re-trained in resuscitation and mentored by the most senior staff. In addition, senior staff were involved earlier in deliveries among high-risk patients. The hospital also acquired more resuscitation equipment, including dedicated Ambu bags, masks and Resuscitaires for the labour ward and theatre. Midwives were re-trained in the use of the partograph. Human resource challenges were addressed with more midwives assigned to the maternity unit.

Training in neonatal resuscitation was done on a three-monthly basis. In 2007, asphyxiated babies were only given 10% dextrose. In subsequent years after the training, the care was expanded to include providing warmth, stimulation and ventilation with Ambu bag and provision of oxygen. This was implemented both in the labour wards and theatre.

In 2009, the case fatality rate for complications of prematurity was observed to begin to rise (Figure 2). This prompted action to address the problem of hypothermia, which was observed to be related to many of the cases. The team introduced Kangaroo Mother Care (KMC) for stable preterm babies. They also recommended the administration of dexamethasone to mothers at risk of preterm birth, and use of radiant warmers in the theatre, labour room and the newborn unit to improve thermal control. For babies with respiratory distress, Continuous Positive Airway Pressure (CPAP) was introduced, and rescue surfactant was made available for use in 2014.

In 2012, light-emitting diode phototherapy was introduced for the management of babies with neonatal jaundice. No additional specific actions to improve the management of neonatal infections were documented as this was not considered a major cause of mortality when compared to intrapartum-

For peer review only - http://bmjopen.bmj.com/site/about/guidelines.xhtml

related hypoxia and complications of prematurity. However, all interventions were observed to be sustained throughout the period under study.Strengths and limitations

This study assessed the introduction of perinatal audits in a low resource setting. There are few studies exploring the use of perinatal death audit to improve care in low-resource settings. In a similar study of 305 perinatal deaths in Tanzania, suboptimal care was identified in 80% of the cases. However, there was no report of changes implemented or on the effect of the changes [9]. While it is difficult to attribute all changes in perinatal outcomes to perinatal death audit alone, the process has helped to identify areas of healthcare that need support for improvement in outcomes. The results should be interpreted in light of the limitations of interrupted time series as the methodology used to assess the effect of an intervention.

Moreover, as there were only two timepoints of data before the introduction of the audits, this limits the capacity of the analysis to examine evidence of a change in slope with the adoption of the intervention.. A third timepoint, as suggested in the Cochrane standards for ITS, would have addressed this issue. Ideally, the results of the study should be confirmed by randomised controlled trials conducted in the same context of care.

Implications for future practice and research

Problems identified through audit:

Perinatal death audit was integrated as part of routine care to help identify deficiencies in the clinical care pathway and to formulate priority actions to ensure improved quality of care and reduced loss of life. Poor use of the partograph and fetal heart monitoring, delay in performing Caesarean sections when these were indicated, poor neonatal resuscitation skills and poor management of preterm babies, were identified as areas requiring improvement in the quality of care. In addition, there was evidence of significant delays incurred when a referral from a lower level healthcare facility occurred. These findings are similar to a study from Tanzania where 47% of perinatal deaths audited had poor fetal heart monitoring, and 23% had delays in performing Caesarean section [9,10].

The interventions for improvement included: training in the use of the partograph, fetal heart monitoring, neonatal resuscitation, measures to reduce delay in performing a Caesarean section by involving doctors in the timely preparation of mothers before transfer to the theatre and the building of a separate maternity theatre. The implementation of these interventions was monitored during the weekly audit

For peer review only - http://bmjopen.bmj.com/site/about/guidelines.xhtml

meetings. Training in neonatal resuscitation has been conducted in Malaysia and Malawi with similar success in the acquisition of skills [11,12].

Interventions for prematurity-related complications:

The interventions implemented included: reduction of hypothermia through the provision of radiant warmers and incubators for the critically-ill babies in the labour wards, theatre and the newborn unit. KMC and immediate skin-to-skin care at birth were initiated in 2009 and 2012, respectively. The use of skin-to-skin care was noted to improve from 40% to 80% of live births after repeated training. KMC has been adopted and is used for all stable low birth weight babies in the newborn unit. A Cochrane systematic review has established that KMC is associated with reduced risk of neonatal mortality (by 51%), nosocomial infection or sepsis (by 58%) and hypothermia (by 77%) [13]. The interventions for reduction of respiratory distress included: use of antenatal steroids, bubble CPAP and use of rescue surfactant. Antenatal steroids, bubble CPAP and rescue surfactant have been found to have low coverage in low-resource settings despite their potential to reduce mortality by 48%, 31% and 32%, respectively [14,15,16]. The majority (98%) of the preterm babies who needed it were able to pay for it and actually received it.This is a private not for profit hospital, majority of the patients that are admitted are able to afford the rates offered by the hospital. Low-cost surfactant was obtained from South Africa / India which cost about 200 dollars per vial. Surfactant is a product that is paid for in our hospital just like other commodities like antibiotics or Caesarean section. The cost of surfactant is less than an operation like Caesarean section.

Intrapartum-related hypoxia and prematurity-related deaths:

In this study, the case fatality rate for intrapartum-related hypoxia was reduced from 21.3% to 15.5%. For stillbirth, intrapartum-related hypoxia has been reported to account for between 3.1% and 25% of all cases of stillbirths in low- and middle-income countries [17]. The change may be attributable to the improvement in resuscitation practices, following training and provision of equipment. Training in neonatal resuscitation has been reported to reduce deaths in babies with intrapartum-related hypoxia by 30% and early neonatal deaths by 38% [18,19]. An analysis of seven facility-based studies estimated that training in neonatal resuscitation reduced the neonatal mortality rate from 43% to 17%. However, a study in Malawi reported that resuscitation training did not reduce mortality [12]. In Malawi, factors such as availability and replacement of resuscitation equipment and mentorship after training were not part of

For peer review only - http://bmjopen.bmj.com/site/about/guidelines.xhtml

the improvement package. Additionally, in our study, the training was repeated on a three-monthly basis and continuous feedback and mentoring were provided which helped ensure continuous improvement in quality. Although these interventions had already been known to work, audit acted as a precursor to implement them.

It is noteworthy that intrapartum-related hypoxia was documented as a cause of death even for macerated stillbirth. Since maceration could set in only six hours post-mortem [20], it is not uncommon in low-resource settings to find a macerated intrapartum death due to delays in an intervention during labour. It could also be because healthcare providers often misclassify fresh stillbirth as macerated stillbirth [21].

Other factors that might have contributed to the reduction in case fatality rates were partograph use, improved fetal heart monitoring and having a dedicated maternity theatre to reduce waiting times for Caesarean section.

The case fatality rate for prematurity was reduced by more than half. One of the major interventions was the introduction of KMC, which has been shown to reduce mortality and morbidity in low-resource settings by 40% and 34%, respectively [13]. Moreover, CPAP was also introduced, which has been reported to improve survival of preterm babies by 27% among resource-limited settings [22]. Finally, in 2014 surfactant administration was introduced, which may have further reduced the mortality in prematurity-related deaths. Although surfactant is not yet provided in many developing countries as demonstrated in this study, it can be given safely under the supervision of experienced personnel.

This study suggests that a significant reduction in case fatality rate for intrapartum-related hypoxia can be expected to occur after the implementation of multiple interventions to improve the inpatient care of small and sick newborns. It further confirms the findings of a Delphi study that estimated supportive care in a Special Care Baby Unit could avert 70% of neonatal deaths due to preterm birth complications, and that 90% could be averted with the availability of Neonatal Intensive Care Units (NICUs) [23].

The fact that only 2.3% of early neonatal deaths were attributed to infection is a surprising finding. In another study, infections accounted for around 14% of early neonatal deaths [24]. Infection is commonest in the lowest socio-economic groups and this could be because most cases of neonatal sepsis were late neonatal deaths (after 1 week) and so would be missed by the audit. Furthermore , 15.4% of

For peer review only - http://bmjopen.bmj.com/site/about/guidelines.xhtml

early neonatal deaths had an unknown cause of death. It is possible that this represents an underestimation of the proportion of deaths attributable to infection. In addition, some cases of respiratory distress syndrome may actually have been cases of neonatal infection, as it is often very difficult to differentiate between the two conditions without sophisticated diagnostic tests.

The relatively large sample size in this study provided a good opportunity to document the distribution of the causes and factors contributing to perinatal mortality. Nevertheless, in 43% of the deaths, a cause of death could not be established. This proportion was highest for macerated stillbirths (88% unknown) with 49% of fresh stillbirths and 15% of early neonatal deaths assigned to the category “unknown”. This may be related to the availability of information about the deaths: the longer the baby died before birth, the more difficult it becomes to obtain information about the circumstances of the death. Furthermore, diagnosis is easier for newborns than for stillbirths since clinicians usually have more time to examine and monitor a sick newborn baby during the period of admission and before death. Another possible reason for the inability to establish the cause of death may be the lack of diagnostic capacity, relating to both staff and equipment. To reduce the proportion of deaths with an unknown cause in low-resource settings, health care providers should keep clinical records as complete and as accurate as possible.

Effectiveness of perinatal death audit:

More and more studies [5,6] have demonstrated that there is some gain in practising perinatal death audit, includinga Cochrane review of 140 studies concluded that the audit could lead to improvements in professional practice. However, review concluded that this depends on how well facilities perform at baseline and how the feedback is provided [25].

Other reports found that perinatal death audit did not improve perinatal outcomes. In a five-year study of health facilities in South Africa, it was observed that among the 54 health facilities that started perinatal death audit, only 30% had a decrease in perinatal mortality, while 35% had an increase [26]. The study identified several facility-related factors that contributed to the increase in perinatal mortality, including lack of use of antenatal steroids, lack of nursing personnel, fetal distress not detected during the antepartum period and incorrect use of the partograph.Additional ITS studies with longer pre-intervention time points of data collection in low and middle-income countries, should confirm or refute our findings. Moreover, perinatal audits studies should include post mortem autopsies, particularly for stillbirth.

For peer review only - http://bmjopen.bmj.com/site/about/guidelines.xhtml

Finally, randomised controlled trials using a stepped wedge design, are needed to assess the effect of perinatal death audit on perinatal outcomes.

CONCLUSION

This study highlights the potential of perinatal death review to identify how care pathways can be improved over time to reduce adverse perinatal outcomes in low-resource settings. This study shows that the introduction of perinatal death audit allowed for the identification of the main causes of death and contributing factors, and prioritise areas in the provision of healthcare that need improvement. However, although the intervention showed a decrease in early neonatal death, the perinatal audit process did not have an impact on PMR and SBR. These findings should encourage more healthcare providers to undertake research on perinatal death reviews, especially in low-resource settings to assess the effectiveness of perinatal death audit.

For peer review only

For peer review only - http://bmjopen.bmj.com/site/about/guidelines.xhtml

DECLARATIONS

Ethical approval and consent to publish

Approval for the study was obtained from the Nsambya Hospital Ethical Review Committee. Individual patient consent was not applicable as the research team extracted anonymized data from case notes.

Acknowledgements

We would like to thank all healthcare providers who took part in the review process. We particularly appreciate the contributions of Dr. Musana Othniel, Dr. Sebunya Robert, Dr. Nazziwa Rita, Dr. Catherine Nyagabyaki, Sr. Nabwami Immaculate, Dr. Tumwine Gilbert and Dr. Zaake.

We are also grateful to Robert Lloyd for his contribution to data processing, to Dr Sarah White for help with part of the statistical analysis, to Prof. Mathews Mathai for reviewing the manuscript and to Caroline Hercod for editing the manuscript.

Disclosure of Interests

All authors declare no competing interests.

Funding

No funding was received for the data collection. However, the cost of data processing, analysis and publication was sponsored by the 'Making it Happen' Programme funded by DFID/UKAid.

Availability of data and materials

The datasets used and/or analysed during the current study are available from the corresponding author upon reasonable request.

Contributions to Authorship

VN contributed to study design, data collection, analysis and writing up. MA processed and analyzed the data and contributed to all drafts of the paper. JED contributed to the conception of the paper, to the analysis, and writing of the paper. RB took part in writing the study proposal, data collection and review

For peer review only - http://bmjopen.bmj.com/site/about/guidelines.xhtml

of cases. PO contributed to the study proposal, data collection and case reviews. NVDB oversaw the conception and analysis and contributed to the initial and final drafts of the paper.

REFERENCES

1. Lawn JE, Blencowe H, Pattinson R, *et al*. Stillbirths: Where? When? Why? How to make the data count? *Lancet*. 2011;**377**:1448-63. doi: 10.1016/S0140-6736(10)62187-3
2. Uganda Bureau of Statistics. Uganda Demographic and Health Survey 2011. Uganda Bureau of Statistics: Kampala 2011. https://dhsprogram.com/pubs/pdf/fr264/fr264.pdf
3. Uganda Ministry of Health, Reproductive Health Division. Annual Maternal and Perinatal Death Review Report 2013/2014. Ministry of Health: Kampala, 2014.
4. Buchmann EJ. Towards greater effectiveness of perinatal death audit in low- and middle-income countries. *BJOG*. 2014;**121**(Supp 4):S134-6. doi: 10.1111/1471-0528.12904
5. Eskes M, Waelput AJM, Erwich JJHM, *et al*. Term perinatal mortality audit in the Netherlands 2010–2012: a population based cohort study. *BMJ Open*. 2014;**4**:e005652. doi: 10.1136/bmjopen-2014-005652
6. Pattinson R, Keber K, Waiswa P, *et al*. Perinatal Mortality Audit: Counting, accountability, and overcoming challenges in scaling up in low- and middle-income countries. *Int J Gynaecol Obstet*. 2009;**109**(Supp 1):113-22. doi: 10.1016/j.ijgo.2009.07.011.
7. World Health Organization. ICD-10 International statistical classification of diseases and related health problems. World Health Organization: Geneva, 2004.
8. Bernal JL, Cummins S, Gasparrini A. Interrupted time series regression for the evaluation of public health interventions: a tutorial. *Int J Epidemiol.* 2017;**46**:348-55 doi: 10.1093/ije/dyw098
9. Kidanto HL, Mogren I, van Roosmalen J, *et al*. Introduction of a qualitative perinatal audit at Muhimbili National Hospital, Dar es Salaam, Tanzania. *BMC Pregn Childb*. 2009;**9**:45. doi: 10.1186/1471-2393-9-45
10. Kidanto HL, Massawe SN, Nyström L, *et al*. Analysis of perinatal mortality at a teaching hospital in Dar es Salaam, Tanzania. *Afr J Reprod Health*. 2006;**10**:72-80.
11. Boo NY. Neonatal resuscitation programme in Malaysia: an eight-year experience. *Singapore Med J*. 2009;**50**:152-9.
12. Hole MK, Olmsted K, Kiromera A, *et al*. Neonatal Resuscitation Curriculum in Malawi, Africa: Did It Change In-Hospital Mortality? *Int J Pediatr*. 2012;**2012**:408689. doi: 10.1155/2012/408689
13. Conde-Agudelo A, Belizán JM, Diaz-Rossello J. Kangaroo mother care to reduce morbidity and mortality in low birthweight infants. *Cochrane Database Syst Rev*. 2011;**3**:CD002771. doi: 10.1002/14651858.CD002771.pub2.
14. Mwansa-Kambafwile J, Cousens S, Hansen T, *et al*. Antenatal steroids in preterm labour for the prevention of neonatal deaths due to complications of preterm birth. *Int J Epidemiol*. 2010;**39**(Supp 1):i122–33. doi: 10.1093/ije/dyq029.
15. Ho JJ, Subramaniam P, Henderson-Smart DJ, *et al*. Continuous distending pressure for respiratory distress syndrome in preterm infants. *Cochrane Database Syst Rev*. 2002;**2**:CD002271.

For peer review only - http://bmjopen.bmj.com/site/about/guidelines.xhtml

16. Soll R, Özek E. Prophylactic animal derived surfactant extract for preventing morbidity and mortality in preterm infants. *Cochrane Database Syst Rev*. 2002;**4**:CD000511.
17. Aminu M, Unkels R, Mdegela M, *et al*. Causes of and factors associated with stillbirth in low- and middle-income countries: a systematic literature review. *BJOG*. 2014;**121**(Supp 4):141-53. doi: 10.1111/1471-0528.12995.
18. Lawn J, Shibuya K, Stein C. No cry at birth: Global estimates of intrapartum stillbirths and intrapartum-related neonatal deaths. *Bull World Health Organ*. 2005;**83**:409-17.
19. Lee ACC, Cousens S, Wall SN, *et al*. Neonatal resuscitation and immediate newborn assessment and stimulation for the prevention of neonatal deaths. *BMC Public Health*. 2011;**11**(Supp 3):S12. doi: 10.1186/1471-2458-11-S3-S12
20. Genest DR, Singer DB. Estimating the time of death in stillborn fetuses: III. External fetal examination; a study of 86 stillborns. *Obstet Gynecol*. 1992;**80**:593-600.
21. Gold KJ, Abdul-Mumin AS, Boggs ME, *et al*. Assessment of “fresh” versus “macerated” as accurate markers of time since intrauterine fetal demise in low income countries. *Int J Gynaecol Obstet*. 2014;**125**:223-7. doi: 10.1016/j.ijgo.2013.12.006
22. Kawaza K, Machen HE, Brown J, *et al*. Efficacy of a low-cost bubble CPAP system in treatment of respiratory distress in a neonatal ward in Malawi. *PLoS One*. 2014;**9**:e86327. doi: 10.1371/journal.pone.0086327
23. Bhutta ZA, Das JK, Bahl R, *et al*. Can available interventions end preventable deaths in mothers, newborn babies, and stillbirths, and at what cost? *Lancet*. 2014;**384**:347-70. doi: 10.1016/S0140-6736(14)60792-3
24. Oza S, Lawn LE, Hogan DR, *et al*. Neonatal cause-of-death estimates for the early and late neonatal periods for 194 countries: 2000-2013. *Bull World Health Organ*. 2015;**93**:19-28. doi: 10.2471/BLT.14.139790
25. Ivers N, Jamtvedt G, Flottorp S, *et al*. Audit and feedback: effects on professional practice and healthcare outcomes. *Cochrane Database Syst Rev*. 2012;**6**:CD000259. doi: 10.1002/14651858.CD000259.pub3
26. Allanson ER, Pattinson RC. Quality-of-care audits and perinatal mortality in South Africa. *Bull World Health Organ*. 2015;**93**:424-8. doi: 10.2471/BLT.14.144683

For peer review only

For peer review only - http://bmjopen.bmj.com/site/about/guidelines.xhtml

Figure and Table Captions

Figure 1: Cause of death for macerated stillbirths (n=142), fresh stillbirths (n=125) and early neonatal deaths (n=261)

Figure 2: Interrupted time series with overdispersion

Table 1: Demographic and clinical characteristics

Table 2: Interrupted time series analysis for selected perinatal outcomes

Supplementary Table 1: Stillbirth rate (per 1000 births), early neonatal mortality rate (per 1000 live births) and perinatal mortality rate (per 1000 births)

Supplementary Table 2: Cause of death for stillbirth and early neonatal death

Supplementary Table 3: Case fatality rates (CFR)

Supplementary Table 4: Standard of care

For peer review only

Table 1: Demographic and clinical characteristics

Characteristics	Fresh stillbirth n=125 (%)	Macerated stillbirth n=142 (%)	Early neonatal death n=259 (%)	Total n=526 (%)
**Referral**				
From other healthcare facility	32 (25.6)	15 (10.6)	37 (14.3)	84 (16.0)
From Traditional Birth Attendant	1 (0.8)	1 (0.7)	1 (0.4)	3 (0.6)
**Parity**				
1	35 (28.0)	48 (33.8)	129 (49.8)	212 (40.3)
2 to 4	64 (51.2)	67 (47.2)	84 (32.4)	215 (40.9)
5 or more	10 (8.0)	15 (10.6)	18 (6.9)	43 (8.2)
**Antenatal care**				
At least 1 ANC visit	107 (85.6)	129 (90.8)	234 (90.3)	470 (89.4)
4 or more ANC visits	38 (30.4)	47 (33.1)	98 (37.8)	183 (34.8)
**Type of pregnancy**				
Single pregnancy	113 (90.4)	131 (92.3)	227 (87.6)	471 (89.5)
Multiple pregnancy	2 (1.6)	5 (3.5)	14 (5.4)	21 (4.0)
**Mode of delivery**				
Spontaneous vaginal delivery	67 (53.6)	123 (86.6)	143 (55.2)	333 (63.3)
Assisted breech delivery	6 (4.8)	2 (1.4)	10 (3.9)	18 (3.4)
Instrumental delivery	4 (3.2)	1 (0.7)	3 (1.2)	8 (1.5)
Caesarean section	45 (36.0)	10 (7.0)	92 (35.5)	147 (27.9)
**Gestational age at delivery**				
<37 weeks	29 (23.2)	56 (39.4)	74 (28.6)	159 (30.2)
37-41 weeks	60 (48.0)	57 (40.1)	132 (51.0)	249 (47.3)
>41 weeks	5 (4.0)	4 (2.8)	9 (3.5)	18 (3.4)
**Baby's sex**				
Male	68 (54.4)	80 (56.3)	146 (56.4)	294 (55.9)
Female	52 (41.6)	57 (40.1)	99 (38.2)	208 (39.5)
**Birthweight**				
<1000g	1 (0.8)	16 (11.3)	9 (3.5)	26 (4.9)
1000-2499g	30 (24.0)	52 (36.6)	81 (31.3)	163 (31.0)
2500-4000g	77 (61.6)	61 (43.0)	149 (57.5)	287 (54.6)
>4000g	3 (2.4)	7 (4.9)	6 (2.3)	16 (3.0)

For peer review only - http://bmjopen.bmj.com/site/about/guidelines.xhtml

Table 2: Interrupted time series analysis for selected perinatal outcomes

Outcome	Without over-dispersion				With over-dispersion			
	Intervention		Time		Intervention		Time	
	IRR (95% CI)	p-value	IRR (95% CI)	p-value	IRR (95% CI)	p-value	IRR (95% CI)	p-value
Perinatal death rate	1.17 (1.02, 1.34)	**0.021**	0.94 (0.92,0.96)	**<0.001**	1.17 (0.79, 1.74)	0.44	0.94 (0.89,1.00)	0.06
Stillbirth rate	1.31 (1.14,1.51)	**<0.001**	0.93 (0.91,0.95)	**<0.001**	1.31 (0.84,2.06)	0.23	0.93 (0.87,1.00)	**0.039**
Early neonatal death rate	0.35 (0.22,0.55)	**<0.001**	1.09 (1.01,1.17)	**0.01**	0.35 (0.22,0.56)	**<0.001**	1.09 (1.01,1.17)	**0.01**
CFR for intrapartum-related hypoxia	0.77 (0.56,1.06)	0.11	0.98 (0.93,1.03)	0.42	0.77 (0.57,1.04)	0.09	0.98 (0.93,1.03)	0.40
CFR for prematurity	1.01 (0.73,1.41)	0.95	0.94 (0.90,0.98)	**0.004**	1.01 (0.64,1.59)	0.96	0.94 (0.88,0.99)	**0.04**
CFR for infections	2.25 (0.38,13.3)	0.37	1.047 (0.86,1.27)	0.68	2.25 (0.17,28.9)	0.54	1.04 (0.78,1.39)	0.77

CFR: Case fatality rate; IRR: Incidence risk ratio

For peer review only

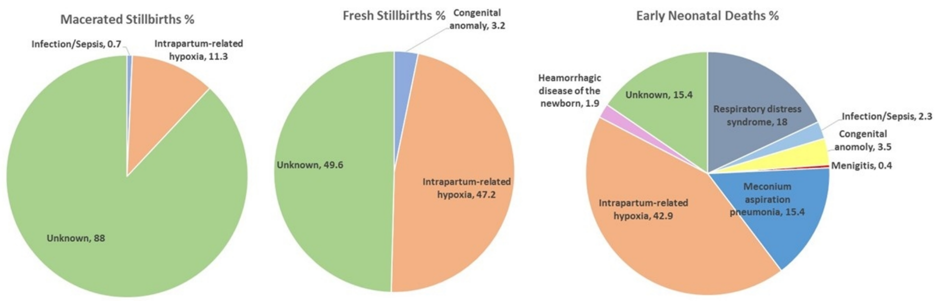

Figure 1: Cause of death for macerated stillbirths (n=142), fresh stillbirths (n=125) and early neonatal deaths (n=261)

338x190mm (96 x 96 DPI)

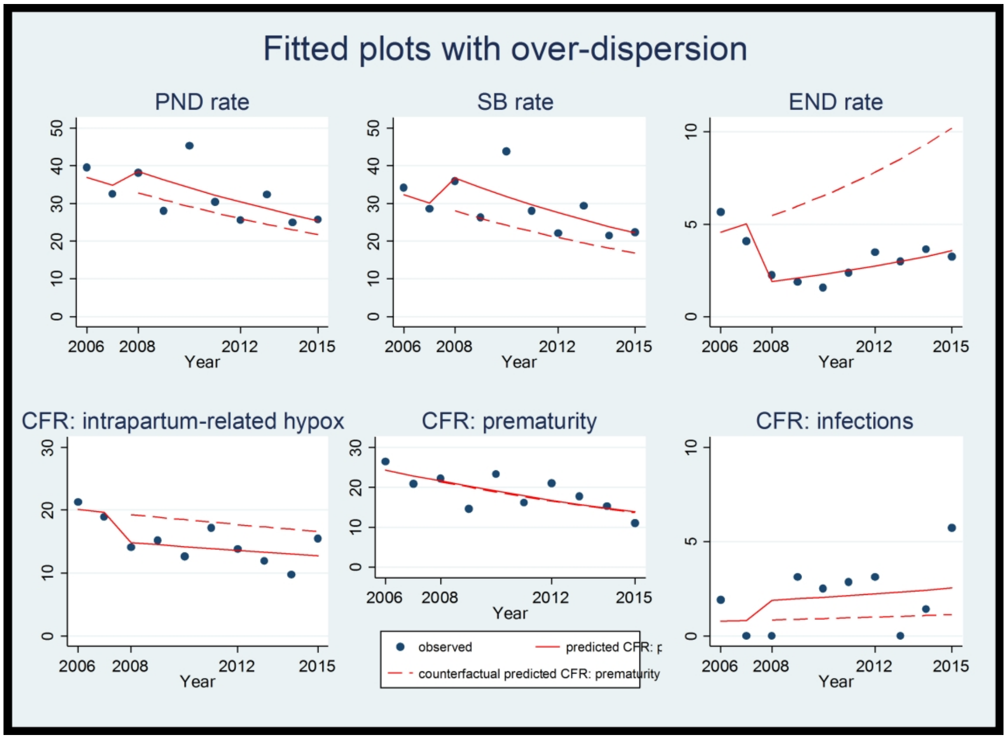

Figure 2: Interrupted time series with overdispersion

Supplementary Materials

Supplementary Table 1: **Stillbirth rate (per 1000 births), early neonatal mortality rate (per 1000 live births) and perinatal mortality rate (per 1000 births).**

Year	Total Births	Live Births	Stillbirth	Stillbirth Rate	Early NND	Early NND Rate	Perinatal Mortality	Perinatal Mortality Rate
2006	6468	6020	213	32.9	96	16	309	47.8
2007	7290	6881	210	28.8	193	28.1	403	55.3
2008	8386	8408	291	34.7	145	17.3	436	52.0
2009	8452	7995	207	24.5	114	14.3	321	38.0
2010	9513	8877	229	24.1	132	14.9	362	38.0
2011	8050	7610	220	27.3	166	21.8	386	47.9
2012	6732	6593	149	22.1	167	25.4	316	47.0
2013	6376	6011	183	28.7	117	19.4	300	47.0
2014	6026	5764	136	22.6	110	19	246	40.8
2015	5462	5222	120	22	112	21.5	232	42.6

For peer review only

<u>Supplementary Table 2:</u> Cause of death for stillbirth and early neonatal death.

Cause of Death	Macerated Stillbirth n=142 (%)	Fresh Stillbirth n=124 (%)	Early Neonatal Death n=259 (%)	TOTAL n=526 (%)
Respiratory distress syndrome	-	-	47 (18.0)	**47 (8.9)**
Infections / Septicaemia	1 (0.7)	0	6 (2.3)	**7 (1.3)**
Congenital anomaly	0	4 (3.2)	9 (3.5)	**13 (2.5)**
Meningitis	-	-	1 (0.4)	**1 (0.2)**
Meconium aspiration pneumonia	-	-	40 (15.4)	**40 (7.6)**
Intrapartum-related hypoxia	16 (11.3)	59 (47.6)	111 (42.9)	**186 (35.4)**
Haemorrhagic disease of newborn	-	-	5 (1.9)	**5 (1.0)**
Unknown	125 (88.0)	61 (49.6)	40 (15.4)	**227 (43.2)**

For peer review only

Supplementary Table 3: Case fatality rates (CFR)

Cause of death		2006	2007	2008	2009	2010	2011	2012	2013	2014	2015
**Intrapartum-related hypoxia**	Intrapartum-related hypoxia admissions	202	291	334	302	316	297	247	252	195	187
	Intrapartum-related hypoxia deaths	43	55	47	46	40	51	34	30	19	29
	**Intrapartum-related hypoxia CFR (%)**	**21.3**	**18.9**	**14.1**	**15.2**	**12.7**	**17.2**	**13.8**	**11.9**	**9.7**	**15.5**
**Prematurity**	Prematurity admissions	163	168	194	246	210	328	286	304	336	371
	Premature deaths	43	35	43	36	49	53	60	54	51	41
	**Prematurity CFR (%)**	**26.4**	**20.8**	**22.2**	**14.6**	**23.3**	**16.2**	**21.0**	**17.8**	**15.2**	**11.1**
**Infections**	Number of admissions for infections	104	149	82	128	120	140	129	171	141	87
	Deaths due to infections	2	0	0	4	3	4	4	0	2	5
	**Infections CFR (%)**	**1.9**	**0**	**0**	**3.1**	**2.5**	**2.9**	**3.1**	**0.0**	**1.4**	**5.7**

For peer review only

Supplementary Table 4: Standard of care

Standard of care	MSB (%)	FSB (%)	ENND (%)	Total (%)
Optimal	82 (67.8)	58 (49.2)	112 (45.7)	262 (53.0)
Probably acceptable	21 (17.4)	28 (23.7)	58 (23.7)	107 (21.7)
Probably sub-optimal	12 (9.9)	24 (20.3)	46 (18.8)	82 (16.6)
Sub-optimal	6 (5.0)	8 (6.8)	29 (11.8)	43 (8.7)
**Total**	**121 (100)**	**118 (100)**	**245 (100)**	**494 (100)**

For peer review only

BMJ Open

Prospective Study to Explore Changes in Quality of Care and Perinatal Outcomes After Implementation of Perinatal Death Audit in Uganda

Journal:	*BMJ Open*
Manuscript ID	bmjopen-2018-027504.R3
Article Type:	Research
Date Submitted by the Author:	12-Nov-2019
Complete List of Authors:	Kirabira, Victoria; Nsambya Hospital<br>Aminu, Mamuda; Liverpool School of Tropical Medicine, Centre for Maternal and Newborn Health ; Liverpool School of Tropical Medicine<br>Dewez, Juan; Liverpool School of Tropical Medicine, Centre for Maternal and Newborn Health<br>Byaruhanga, Romano; Nsambya Hospital<br>Okong, Pius; Nsambya Hospital<br>van den Broek, Nynke; Liverpool School of Tropical Medicine, Centre for Maternal and Newborn Health
**Primary Subject Heading**:	Obstetrics and gynaecology
Secondary Subject Heading:	Global health, Paediatrics, Public health, Reproductive medicine
Keywords:	AUDIT, Reproductive medicine < GYNAECOLOGY, Clinical audit < HEALTH SERVICES ADMINISTRATION & MANAGEMENT, Quality in health care < HEALTH SERVICES ADMINISTRATION & MANAGEMENT, stillbirth, neonatal death

SCHOLARONE™
Manuscripts

I, the Submitting Author has the right to grant and does grant on behalf of all authors of the Work (as defined in the below author licence), an exclusive licence and/or a non-exclusive licence for contributions from authors who are: i) UK Crown employees; ii) where BMJ has agreed a CC-BY licence shall apply, and/or iii) in accordance with the terms applicable for US Federal Government officers or employees acting as part of their official duties; on a worldwide, perpetual, irrevocable, royalty-free basis to BMJ Publishing Group Ltd ("BMJ") its licensees and where the relevant Journal is co-owned by BMJ to the co-owners of the Journal, to publish the Work in this journal and any other BMJ products and to exploit all rights, as set out in our licence.

The Submitting Author accepts and understands that any supply made under these terms is made by BMJ to the Submitting Author unless you are acting as an employee on behalf of your employer or a postgraduate student of an affiliated institution which is paying any applicable article publishing charge ("APC") for Open Access articles. Where the Submitting Author wishes to make the Work available on an Open Access basis (and intends to pay the relevant APC), the terms of reuse of such Open Access shall be governed by a Creative Commons licence – details of these licences and which Creative Commons licence will apply to this Work are set out in our licence referred to above.

Other than as permitted in any relevant BMJ Author's Self Archiving Policies, I confirm this Work has not been accepted for publication elsewhere, is not being considered for publication elsewhere and does not duplicate material already published. I confirm all authors consent to publication of this Work and authorise the granting of this licence.

For peer review only

Prospective Study to Explore Changes in Quality of Care and Perinatal Outcomes After Implementation of Perinatal Death Audit in Uganda

---

Victoria Nakibuuka Kirabira[a], Mamuda Aminu[b§], Juan Emmanuel Dewez[b], Romano Byaruhanga[a], Pius Okong[a], Nynke van den Broek[b]

[a] Nsambya Hospital, P.O. Box 7146, Kampala, Uganda

[b] Centre for Maternal and Newborn Health, Liverpool School of Tropical Medicine, Pembroke Place, Liverpool L3 5QA, UK

[§]Corresponding Author Contact Details:

Dr Mamuda Aminu
Centre for Maternal and Newborn Health,
Liverpool School of Tropical Medicine, Pembroke Place, Liverpool L3 5QA.
Tel: +44(0)151 705 3342
Email: Mamuda.Aminu@lstmed.ac.uk

Author email addresses:

VN Kirabira	nakibuukarv@yahoo.com
JE Dewez	manuel.dewez@lshtm.ac.uk
R Byaruhanga	byaruhangarn@yahoo.com
P Okong	puisokong@yahoo.com
NR van den Broek	Nynke.Broek@lstmed.ac.uk

Running Title: Perinatal death audit in Uganda

Keywords: perinatal death, cause of perinatal death, quality improvement

***

Word Count (Body Only): 3680

Abstract: 300

For peer review only

ABSTRACT

Objective: To assess the effects of perinatal death audit on perinatal outcomes in a tertiary hospital in Kampala

Design: Interrupted time series (ITS) analysis

Setting: Nsambya Hospital, Uganda

Participants: Live births and stillbirths

Interventions: Perinatal death audit

Primary and secondary outcome measures: Primary outcomes: perinatal mortality rate, stillbirth rate, early neonatal mortality rate. Secondary outcomes: case fatality rates (CFR) for asphyxia, complications of prematurity and neonatal sepsis.

Results

526 perinatal deaths were audited: 142 (27.0%) fresh stillbirths, 125 (23.8%) macerated stillbirths and 259 (49.2%) early neonatal deaths.

The ITS analysis showed a decrease in perinatal death (PND) rates without the introduction of perinatal death audits (IRR (95% CI) for time=0.94, p<0.001), but an increase in PND (IRR (95% CI) =1.17 (1.0,-1.34), p=0.0021) following the intervention. However, when over-dispersion was included in the model, there were no statistically significant differences in PND with or without the intervention (p=0.06 and p=0.44 respectively).

Stillbirth rates exhibited a similar pattern. By contrast, early neonatal death rates showed an overall upward trend without the intervention (IRR (95% CI)= 1.09 (1.01-1.17), p=0.01), but a decrease following the introduction of the perinatal death audits (IRR (95% CI)= 0.35 (0.22-0.56), p<0.001), when over-dispersion was included.

The CFR for prematurity showed a downward trend over time (IRR (95% CI) =0.94 (0.88-0.99), p=0.04) but not for the intervention. With regards CFRs for intrapartum-related hypoxia or infection, no statistically significant effect was detected for either time or the intervention.

Conclusion

The introduction of perinatal death audit showed no statistically significant effect on perinatal mortality or stillbirth rate, but a significant decrease in early neonatal mortality rate. No effect was detected on case fatality rates for prematurity, intrapartum-related hypoxia or infections. These findings should encourage more research to assess the effectiveness of perinatal death reviews on perinatal deaths in general, but also on stillbirths and neonatal deaths in particular, in low-resource settings.

For peer review only - http://bmjopen.bmj.com/site/about/guidelines.xhtml

Strengths and limitations of this study

- This study is one of the few studies assessing the effect of perinatal death audits in a resource-limited setting.
- The inherent limitation of the methods used to assess the cause of death was the dependence on hospital records, which were often incomplete, inaccurate or both.
- The interrupted time series design was useful to assess the effect of the intervention, but only two time points of data were documented before the introduction of the audit, which could have influenced the effect on outcomes of interest.
- Additional studies, such as ITS studies with longer pre-intervention time points of data collection, stepped-wedge randomised controlled trials, and perhaps post mortem autopsies, must confirm the findings of this study.

For peer review only

For peer review only - http://bmjopen.bmj.com/site/about/guidelines.xhtml

BACKGROUND

Currently, about 2.7 million neonatal deaths and 2.6 million stillbirths occur worldwide every year. Of these, the majority occur in low- and middle-income countries, and more than half (55%) of the global stillbirths occur in sub-Saharan Africa and South Asia [1]. Uganda's perinatal mortality is estimated to be 36 deaths per 1000 births and remains unacceptably high. Almost 50% of these are fresh stillbirths [2]. The neonatal mortality rate (21 per 1000 live births) in Uganda is also high, contributing 50% of the infant mortality rate [3]. It is crucial that interventions are put in place to reduce preventable neonatal deaths and stillbirths.

Perinatal death audit (or review) can be used to identify areas of care which can be strengthened to improve perinatal outcomes [4]. It involves reviewing cases of mortality with a view to identifying avoidable factors and implementing changes to prevent future deaths. A population-based cohort study of 943 perinatal mortalities that occurred in 90 hospitals in the Netherlands reported a decrease in substandard care (from 10% to 5%) and perinatal mortality (from 2.3 to 2.0/1000 births; $p<0.00001$) after the introduction of perinatal death audit [5].

Similarly, in a systematic review focusing on low- and middle-income countries, a meta-analysis of 7 before-and-after studies reported a 30% (95% confidence interval, 21%–38%) reduction in perinatal mortality after introduction of perinatal death audit in healthcare facilities [6]. However, the authors considered most studies included in the review as of low quality. Thus, there is a paucity of data from low-resource settings on the use of and effectiveness of perinatal death audit to improve the quality of care provided to mothers and their babies. The systematic review recommended more research in key areas, including exploring the effect of facility-based perinatal death audit on changing the quality of care [6].

The aim of this study is to assess the changes in perinatal outcomes observed after the introduction of perinatal death audit in a tertiary hospital in Uganda and to describe the changes in healthcare provided to mother and babies resulting from the perinatal death audits.

METHODS

Study setting

For peer review only - http://bmjopen.bmj.com/site/about/guidelines.xhtml

This study was conducted at Nsambya Hospital, a private not-for-profit tertiary hospital in Kampala, Uganda, which serves as a referral centre for Makindye Health Sub-district in Kampala. The facility receives 7,000 deliveries per year and provides 24-hour comprehensive emergency obstetric and newborn care services, with a staff of 6 obstetricians, 18 resident doctors, 5 intern doctors and 40 midwives.

The newborn care unit admits 2,500 babies annually and has a paediatrician/neonatologist, two residents and up to four midwives on duty per shift. It has 40 beds, including 10 neonatal intensive beds, and provides essential newborn care including; oxygen therapy, surfactant and low-cost bubble continuous positive airway pressure (CPAP), kangaroo mother care and phototherapy.

Data collection

Perinatal death audit using locally developed tools and guidelines started in 2008 and was led by a multidisciplinary audit team comprising of obstetricians, paediatricians, resident doctors, midwives and administrators. Team meetings were held weekly, chaired by the most senior obstetrician or paediatrician. Perinatal death was defined as "death of a fetus greater than a birth weight of 1000g if measured, or a gestational age of at least 28 completed weeks or more, or a body length (crown-heel) of at least 35cm or more"[7] and included all stillbirths and deaths within the first week of life (early neonatal deaths). The number of perinatal deaths was documented daily using clinical handover reports and registers in the labour ward and neonatal special care unit. Due to the large numbers of deaths, one-quarter of the cases (every 4th case) were selected for audit from each of the following categories: fresh stillbirths, macerated stillbirths and early neonatal deaths.

The number of admissions in the newborn unit, reasons for admission, death and case fatalities rates for intrapartum-related hypoxia, infection and prematurity were collected for the period before the perinatal audit was introduced (2006-2007) and subsequently for the period after commencement of the perinatal death audit (2008-2015). The data was obtained and aggregated monthly through a retrospective analysis of registers and patient records. Numbers of live and total births were also documented to enable rates calculation.

Intervention: the audit process

For each case selected for review, an audit form was completed by a resident doctor, who also provided a narrative summary for each case. Sources of information included: clinical handover books, maternity

For peer review only - http://bmjopen.bmj.com/site/about/guidelines.xhtml

registers, newborn admission register, and clinical notes for mothers and newborns. Each case was then presented to the rest of the audit team for detailed discussion. The information presented to the team allowed reviewing the cause of death and assigning a more precise cause of death by consensus. Care provided was judged against existing local protocols and national guidelines. No clinical autopsies were performed due to lack of a full-time pathologist at the hospital.

The quality of care provided at the hospital was also graded by consensus and classified into four categories:

- Optimal care generally accepted practice and all standards of care were followed.
- Suboptimal care but different management would have made no difference to the outcome (probably acceptable)
- Suboptimal care where different management might have made a difference to the outcome - an avoidable factor of uncertain clarity or influence on the outcome (probably suboptimal)
- Different management would reasonably be expected to have made a difference to the outcome. A clearly avoidable factor implying that the adverse outcome could have been prevented) (suboptimal)

The overall grading of the care provided to the mother and baby, the identified specific areas of suboptimal care and the recommendations for improvement were documented. This grading included referred mothers as well, whereas there were delays from the referring site, suboptimal care can also occur in tertiary hospital as well, such as delay in performing a Caesarean section

The summary of each case included a list of the actions to be taken. In each case, a specific person was appointed to lead the implementation of the relevant actions. During monthly meetings, implementation progress was compared with the action plan by the wider audit team. A number of interventions, such as neonatal resuscitation training, the introduction of Kangaroo Mother Care (KMC), building of a new maternity theatre, were thus introduced gradually over the years to address the gaps in care. Whereas maternal death reviews had been introduced before the perinatal death audits, they emphasized more care to the mother than the baby.

Data analysis

For peer review only - http://bmjopen.bmj.com/site/about/guidelines.xhtml

For peer review only

SPSS© (IBM, version 22) was used for descriptive analyses. Stillbirth and perinatal mortality rates were expressed per 1000 total births, while the early neonatal mortality rate was expressed per 1000 live births. Case fatality rate (CFR) was defined as the proportion of deaths among cases diagnosed with the disease. Statistical significance was calculated at 95% confidence interval or p-value of <0.05.

Data for the six primary and secondary outcomes were each analysed using interrupted time series (ITS) models in Stata©, following Cochrane checklist for assessing the quality of ITS studies as a guide. Poisson regression models of counts were fitted with covariates for intervention and year, and the relevant population size (log-transformed) used as the offset. As proposed by Bernal et al [8] over-dispersion was considered (by using an additional option: scale(x2)) since it often arises in the context of count data. Auto-correlation was also inspected using plots. The 5% level was used to determine statistical significance.

Since low birth weight and prematurity are both associated with a higher risk of perinatal mortality, a one-way between-groups analysis of variance (ANOVA) was conducted to explore changes in birth weight and gestational age over time.

A narrative summary description was used to report actions agreed and implemented following an audit. These were documented chronologically as derived from minutes of the audit meetings.

Ethical approval

Approval for the study was obtained from the Nsambya Hospital Ethical Review Committee.

Patient and public involvement

Individual patient consent was not applicable as the research team extracted anonymized data from case notes. Patients and the public were not involved in this study.

RESULTS

Demographic and clinical characteristics:

A total of 58,997 births and 2,616 perinatal deaths occurred between 2008 and 2015 (Supplementary Table 1). Of these, 603 (23%) perinatal deaths were selected for audit. After review, 526 were found to

For peer review only - http://bmjopen.bmj.com/site/about/guidelines.xhtml

meet the definition of perinatal mortality: 142 (27.0%) fresh stillbirths, 125 (23.8%) macerated stillbirths and 259 (49.2%) early neonatal deaths (Table 1).

The majority of cases came to the hospital directly, with 17% referrals. Mothers had a mean age of 27.3 years (SD: 5.1); median parity of 2 (SD of 1.6). Just over one-third of fresh stillbirths and early neonatal deaths were delivered by Caesarean section. The mean birth weight of babies was 2610 grams (SD: 969.4). Between 23.2% and 39.4% were born prematurely (Table 1).

Cause of death:

Overall, intrapartum-related hypoxia was the leading cause of perinatal mortality in this setting, accounting for 35.4% of all cases (Figure 1 and Supplementary Table 2). This is followed by respiratory distress syndrome (8.9%) and meconium aspiration pneumonia (7.6%). Cause of death in 43.2% could not be ascertained: 15.4% for neonatal deaths, 49.2% for fresh stillbirths and 88.0% for macerated stillbirths. However, the proportion of deaths for which cause of death could not be established ("cause unknown") decreased from 34.1% (overall) in 2008 to 2.7% in 2013.

Trend in perinatal mortality rate:

The ITS analysis showed that, without modelling over-dispersion, there would have been a downward trend (a decrease) in perinatal death rates (Incidence risk ratio (IRR) (95% CI) for time=0.94, p<0.001), while a statistically significant increase (IRR (95% CI) =1.17 (1.0,-1.34), p=0.0021) was observed following the introduction of perinatal death audits. However, when over-dispersion was included in the model, both effects were not statistically significant (Table 2, Figure 2, and Supplementary Table 3).

Stillbirth rates exhibited a similar pattern. By contrast, early neonatal death rates showed an overall upward trend (IRR (95% CI)= 1.09 (1.01-1.17), p=0.01) but a a decrease following the introduction of the perinatal death audits (IRR (95% CI) = 0.35 (0.22-0.56), p<0.001) when over-dispersion was included in the model (Table 2, and Figures 2). The ANOVA to explore changes in birth weight and gestational age over time showed no statistically significant change in birthweight over time (p = 0.872), and no change in gestational age at birth over time (p = 0.219).

The CFR for prematurity showed a downward trend over time (IRR (95% CI)=0.94 (0.88-0.99), p=0.04) but a downward trend was not observed with the intervention. With regards to the CFRs for intrapartum-

For peer review only - http://bmjopen.bmj.com/site/about/guidelines.xhtml

related hypoxia and infections, no statistically significant effect was detected with or without the intervention (Table 2, Figures 2, and Supplementary Table 3).

For each outcome considered, the autocorrelation and partial correlation plots provided no evidence that there was autocorrelation not accounted for in the models used.

Quality improvement:

More than half of the cases (53%) were deemed to have had optimal care. The rest had probably acceptable care (21.7%), probably sub-optimal care (16.6%) or sub-optimal care (8.7%) (Supplementary Table 4). Among the different types of mortalities, sub-optimal care was most common among early neonatal deaths (11.8%), as compared to fresh stillbirth (6.8%) and macerated stillbirth (5.0%).

DISCUSSION

Main findings

This study assessed the effect of perinatal death audit to improve the quality of perinatal care and perinatal outcomes in a tertiary hospital in Uganda over a period of 10 years.

Intrapartum-related hypoxia was the leading cause of perinatal mortality (accounting for 35.4%), followed by respiratory distress syndrome (8.9%) and meconium aspiration pneumonia (7.6%). Cause of death was unknown in 43.2% of cases. The proportion of deaths with unknown cause decreased from 34.1% (overall) in 2008 to 2.7% in 2013.

There was no effect of perinatal death audit on perinatal mortality or stillbirth rates. However, the intervention led to a decrease in neonatal mortality rate [IRR (95% CI)= 0.35 (0.22-0.56), p<0.001)]. It was noted that the high numbers of stillbirth inflated the numbers of perinatal death. Thus, even though the audit made a difference to early neonatal deaths, this difference is neutralised when combined with stillbirths.

The CFR for prematurity showed a downward trend over time (IRR (95% CI)=0.94 (0.88-0.99), p=0.04), but this was not related to the perinatal death audits. The CFRs for intrapartum-related hypoxia and infections were not affected by the intervention.

For peer review only - http://bmjopen.bmj.com/site/about/guidelines.xhtml

More than half of the cases (53%) were deemed to have had optimal care, with the rest of the cases having varying levels of acceptability. Early neonatal deaths had the highest proportion of cases with suboptimal care (11.8%). Case fatality rates decreased between 2006 and 2015 for intrapartum-related hypoxia (21.3% to 15.5%) and for complications of prematurity (26.4% to 11.1%). However, case fatality rates for infection increased from 1.9% in 2006 to 5.7% in 2015.

Quality improvement

In 2007 and 2008, the perinatal death review team observed many cases of delay between the decision for, and the actual conduct of, Caesarean Section, which was associated with high numbers of intrapartum-related hypoxia. To reduce the time taken to prepare mothers for the procedure, the team recommended the involvement of doctors in the preparation. These ensured mothers were catheterized and blood samples were taken for grouping and cross-matching at the time of decision. During the same period, a new dedicated operating theatre was built close to the maternity ward.

In 2008, two major factors contributing to perinatal mortality were identified to have recurred consistently: poor neonatal resuscitation and poor use of the partographs. Both of these were thought to contribute to perinatal death due to intrapartum-related hypoxia. To tackle the problem of poor resuscitation skills, maternity staff were re-trained in resuscitation and mentored by the most senior staff. In addition, senior staff were involved earlier in deliveries among high-risk patients. The hospital also acquired more resuscitation equipment, including dedicated Ambu bags, masks and Resuscitaires for the labour ward and theatre. Midwives were re-trained in the use of the partograph. Human resource challenges were addressed with more midwives assigned to the maternity unit.

Training in neonatal resuscitation was done on a three-monthly basis. In 2007, asphyxiated babies were only given 10% dextrose. In subsequent years after the training, the care was expanded to include providing warmth, stimulation and ventilation with Ambu bag and provision of oxygen. This was implemented both in the labour wards and theatre.

In 2009, the case fatality rate for complications of prematurity was observed to begin to rise (Figure 2). This prompted action to address the problem of hypothermia, which was observed to be related to many of the cases. The team introduced Kangaroo Mother Care (KMC) for stable preterm babies. They also recommended the administration of dexamethasone to mothers at risk of preterm birth, and use of radiant warmers in the theatre, labour room and the newborn unit to improve thermal control. For babies

For peer review only - http://bmjopen.bmj.com/site/about/guidelines.xhtml

with respiratory distress, Continuous Positive Airway Pressure (CPAP) was introduced, and rescue surfactant was made available for use in 2014.

In 2012, light-emitting diode phototherapy was introduced for the management of babies with neonatal jaundice. No additional specific actions to improve the management of neonatal infections were documented as this was not considered a major cause of mortality when compared to intrapartum-related hypoxia and complications of prematurity. However, all interventions were observed to be sustained throughout the period under study. This study assessed the introduction of perinatal audits in a low resource setting. There are few studies exploring the use of perinatal death audit to improve care in low-resource settings. In a similar study of 305 perinatal deaths in Tanzania, suboptimal care was identified in 80% of the cases. However, there was no report of changes implemented or on the effect of the changes [9].

Strengths and limitations

This study is one of the few studies assessing the effect of perinatal death audits in a resource-limited setting, over a period of ten years. While it is difficult to attribute all changes in perinatal outcomes to perinatal death audit alone, the process has helped to identify areas of healthcare that need support for improvement in outcomes.

The results should be interpreted in light of the limitations. The attribution of cause of death was based on hospital records, which were often incomplete, inaccurate or both. In terms of the ITS design, there were only two timepoints of data before the introduction of the audits, which limits the capacity of the analysis to examine evidence of a change in slope with the adoption of the intervention. A third timepoint, as suggested in the Cochrane standards for ITS, would have addressed this issue.

Implications for future practice and research

Problems identified through audit:

Perinatal death audit was integrated as part of routine care to help identify deficiencies in the clinical care pathway and to formulate priority actions to ensure improved quality of care and reduced loss of life. Poor use of the partograph and fetal heart monitoring, delay in performing Caesarean sections when these were indicated, poor neonatal resuscitation skills and poor management of preterm babies, were

For peer review only - http://bmjopen.bmj.com/site/about/guidelines.xhtml

identified as areas requiring improvement in the quality of care. In addition, there was evidence of significant delays incurred when a referral from a lower level healthcare facility occurred. These findings are similar to a study from Tanzania where 47% of perinatal deaths audited had poor fetal heart monitoring, and 23% had delays in performing Caesarean section [9,10].

The interventions for improvement included: training in the use of the partograph, fetal heart monitoring, neonatal resuscitation, measures to reduce delay in performing a Caesarean section by involving doctors in the timely preparation of mothers before transfer to the theatre and the building of a separate maternity theatre. The implementation of these interventions was monitored during the weekly audit meetings. Training in neonatal resuscitation has been conducted in Malaysia and Malawi with similar success in the acquisition of skills [11,12].

Interventions for prematurity-related complications:

The interventions implemented included: reduction of hypothermia through the provision of radiant warmers and incubators for the critically-ill babies in the labour wards, theatre and the newborn unit. KMC and immediate skin-to-skin care at birth were initiated in 2009 and 2012, respectively. The use of skin-to-skin care was noted to improve from 40% to 80% of live births after repeated training. KMC has been adopted and is used for all stable low birth weight babies in the newborn unit. A Cochrane systematic review has established that KMC is associated with reduced risk of neonatal mortality (by 51%), nosocomial infection or sepsis (by 58%) and hypothermia (by 77%) [13]. The interventions for reduction of respiratory distress included: use of antenatal steroids, bubble CPAP and use of rescue surfactant. Antenatal steroids, bubble CPAP and rescue surfactant have been found to have low coverage in low-resource settings despite their potential to reduce mortality by 48%, 31% and 32%, respectively [14,15,16]. The majority (98%) of the preterm babies who needed it were able to pay for it and actually received it. This is a private not for profit hospital, majority of the patients that are admitted are able to afford the rates offered by the hospital. Low-cost surfactant was obtained from South Africa / India which cost about 200 dollars per vial. Surfactant is a product that is paid for in our hospital just like other commodities like antibiotics or Caesarean section. The cost of surfactant is less than an operation like Caesarean section.

Intrapartum-related hypoxia and prematurity-related deaths:

For peer review only - http://bmjopen.bmj.com/site/about/guidelines.xhtml

In this study, the case fatality rate for intrapartum-related hypoxia was reduced from 21.3% to 15.5%. For stillbirth, intrapartum-related hypoxia has been reported to account for between 3.1% and 25% of all cases of stillbirths in low- and middle-income countries [17]. The change may be attributable to the improvement in resuscitation practices, following training and provision of equipment. Training in neonatal resuscitation has been reported to reduce deaths in babies with intrapartum-related hypoxia by 30% and early neonatal deaths by 38% [18,19]. An analysis of seven facility-based studies estimated that training in neonatal resuscitation reduced the neonatal mortality rate from 43% to 17%. However, a study in Malawi reported that resuscitation training did not reduce mortality [12]. In Malawi, factors such as availability and replacement of resuscitation equipment and mentorship after training were not part of the improvement package. Additionally, in our study, the training was repeated on a three-monthly basis and continuous feedback and mentoring were provided which helped ensure continuous improvement in quality. Although these interventions had already been known to work, audit acted as a precursor to implement them.

It is noteworthy that intrapartum-related hypoxia was documented as a cause of death even for macerated stillbirth. Since maceration could set in only six hours post-mortem [20], it is not uncommon in low-resource settings to find a macerated intrapartum death due to delays in an intervention during labour. It could also be because healthcare providers often misclassify fresh stillbirth as macerated stillbirth [21].

Other factors that might have contributed to the reduction in case fatality rates were partograph use, improved fetal heart monitoring and having a dedicated maternity theatre to reduce waiting times for Caesarean section.

The case fatality rate for prematurity was reduced by more than half. One of the major interventions was the introduction of KMC, which has been shown to reduce mortality and morbidity in low-resource settings by 40% and 34%, respectively [13]. Moreover, CPAP was also introduced, which has been reported to improve survival of preterm babies by 27% among resource-limited settings [22]. Finally, in 2014 surfactant administration was introduced, which may have further reduced the mortality in prematurity-related deaths. Although surfactant is not yet provided in many developing countries as demonstrated in this study, it can be given safely under the supervision of experienced personnel.

For peer review only - http://bmjopen.bmj.com/site/about/guidelines.xhtml

This study suggests that a significant reduction in case fatality rate for intrapartum-related hypoxia can be expected to occur after the implementation of multiple interventions to improve the inpatient care of small and sick newborns. It further confirms the findings of a Delphi study that estimated supportive care in a Special Care Baby Unit could avert 70% of neonatal deaths due to preterm birth complications, and that 90% could be averted with the availability of Neonatal Intensive Care Units (NICUs) [23].

The fact that only 2.3% of early neonatal deaths were attributed to infection is a surprising finding. In another study, infections accounted for around 14% of early neonatal deaths [24]. Infection is commonest in the lowest socio-economic groups and this could be because most cases of neonatal sepsis were late neonatal deaths (after 1 week) and so would be missed by the audit. Furthermore, 15.4% of early neonatal deaths had an unknown cause of death. It is possible that this represents an underestimation of the proportion of deaths attributable to infection. In addition, some cases of respiratory distress syndrome may actually have been cases of neonatal infection, as it is often very difficult to differentiate between the two conditions without sophisticated diagnostic tests.

The relatively large sample size in this study provided a good opportunity to document the distribution of the causes and factors contributing to perinatal mortality. Nevertheless, in 43% of the deaths, a cause of death could not be established. This proportion was highest for macerated stillbirths (88% unknown) with 49% of fresh stillbirths and 15% of early neonatal deaths assigned to the category “unknown”. This may be related to the availability of information about the deaths: the longer the baby died before birth, the more difficult it becomes to obtain information about the circumstances of the death. Furthermore, diagnosis is easier for newborns than for stillbirths since clinicians usually have more time to examine and monitor a sick newborn baby during the period of admission and before death. Another possible reason for the inability to establish the cause of death may be the lack of diagnostic capacity, relating to both staff and equipment. To reduce the proportion of deaths with an unknown cause in low-resource settings, health care providers should keep clinical records as complete and as accurate as possible.

<u>Effectiveness of perinatal death audit:</u>

Ideally, the results of this study should be confirmed by randomised controlled trials conducted in the same context of care.

For peer review only - http://bmjopen.bmj.com/site/about/guidelines.xhtml

Other reports found that perinatal death audit did not improve perinatal outcomes. In a five-year study of health facilities in South Africa, it was observed that among the 54 health facilities that started perinatal death audit, only 30% had a decrease in perinatal mortality, while 35% had an increase [25]. The study identified several facility-related factors that contributed to the increase in perinatal mortality, including lack of use of antenatal steroids, lack of nursing personnel, fetal distress not detected during the antepartum period and incorrect use of the partograph. Additional ITS studies with longer pre-intervention time points of data collection in low and middle-income countries should confirm or refute our findings understanding of the effects of this intervention in these settings. Finally, randomised controlled trials using a stepped wedge design, are needed to assess the effect of perinatal death audit on perinatal outcomes.

A systematic review and meta-analysis of the effect of perinatal audits in low-resource settings showed a reduction of perinatal deaths of 30% attributable to this intervention [6]. A large randomized controlled trial conducted in Mali and Senegal which aimed to assess the effect of a complex intervention (including maternal death audits) on maternal and perinatal outcomes, showed that maternal audits reduced ENMR by 24%, while there was no effect on SBR [26].These findings on perinatal outcomes are in line with those of our study, even though the intervention was different (we assessed perinatal death audits, while the RCT assessed maternal death audits). This suggests that although perinatal death audits seem promising in reducing perinatal mortality, the role of these audits on SBR and ENMR remains unclear.

CONCLUSION

This study highlights the potential of perinatal death review to identify how care pathways can be improved over time to reduce adverse perinatal outcomes in low-resource settings. This study shows that the introduction of perinatal death audit allowed for the identification of the main causes of death and contributing factors, and prioritise areas in the provision of healthcare that need improvement. However, although the intervention showed a decrease in early neonatal death, the perinatal audit process did not have an impact on PMR and SBR. These findings should encourage more healthcare providers to undertake research on perinatal death reviews, especially in low-resource settings to assess the effectiveness of perinatal death audit on perinatal deaths in general, but also on stillbirths and neonatal deaths in particular.

For peer review only - http://bmjopen.bmj.com/site/about/guidelines.xhtml

DECLARATIONS

Ethical approval and consent to publish

Approval for the study was obtained from the Nsambya Hospital Ethical Review Committee. Individual patient consent was not applicable as the research team extracted anonymized data from case notes.

Acknowledgements

We would like to thank all healthcare providers who took part in the review process. We particularly appreciate the contributions of Dr. Musana Othniel, Dr. Sebunya Robert, Dr. Nazziwa Rita, Dr. Catherine Nyagabyaki, Sr. Nabwami Immaculate, Dr. Tumwine Gilbert and Dr. Zaake.

We are also grateful to Robert Lloyd for his contribution to data processing, to Dr Sarah White for help with part of the statistical analysis, to Prof. Mathews Mathai for reviewing the manuscript and to Caroline Hercod for editing the manuscript.

Disclosure of Interests

All authors declare no competing interests.

Funding

No funding was received for the data collection. However, the cost of data processing, analysis and publication was sponsored by the 'Making it Happen' Programme funded by DFID/UKAid.

Availability of data and materials

The datasets used and/or analysed during the current study are available from the corresponding author upon reasonable request.

Contributions to Authorship

VN contributed to study design, data collection, analysis and writing up. MA processed and analyzed the data and contributed to all drafts of the paper. JED contributed to the conception of the paper, to the analysis, and writing of the paper. RB took part in writing the study proposal, data collection and review

For peer review only - http://bmjopen.bmj.com/site/about/guidelines.xhtml

of cases. PO contributed to the study proposal, data collection and case reviews. NVDB oversaw the conception and analysis and contributed to the initial and final drafts of the paper.

REFERENCES

1. Lawn JE, Blencowe H, Pattinson R, *et al*. Stillbirths: Where? When? Why? How to make the data count? *Lancet*. 2011;**377**:1448-63. doi: 10.1016/S0140-6736(10)62187-3
2. Uganda Bureau of Statistics. Uganda Demographic and Health Survey 2011. Uganda Bureau of Statistics: Kampala 2011. https://dhsprogram.com/pubs/pdf/fr264/fr264.pdf
3. Uganda Ministry of Health, Reproductive Health Division. Annual Maternal and Perinatal Death Review Report 2013/2014. Ministry of Health: Kampala, 2014.
4. Buchmann EJ. Towards greater effectiveness of perinatal death audit in low- and middle-income countries. *BJOG*. 2014;**121**(Supp 4):S134-6. doi: 10.1111/1471-0528.12904
5. Eskes M, Waelput AJM, Erwich JJHM, *et al*. Term perinatal mortality audit in the Netherlands 2010–2012: a population based cohort study. *BMJ Open*. 2014;**4**:e005652. doi: 10.1136/bmjopen-2014-005652
6. Pattinson R, Keber K, Waiswa P, *et al*. Perinatal Mortality Audit: Counting, accountability, and overcoming challenges in scaling up in low- and middle-income countries. *Int J Gynaecol Obstet*. 2009;**109**(Supp 1):113-22. doi: 10.1016/j.ijgo.2009.07.011.
7. World Health Organization. ICD-10 International statistical classification of diseases and related health problems. World Health Organization: Geneva, 2004.
8. Bernal JL, Cummins S, Gasparrini A. Interrupted time series regression for the evaluation of public health interventions: a tutorial. *Int J Epidemiol.* 2017;**46**:348-55 doi: 10.1093/ije/dyw098
9. Kidanto HL, Mogren I, van Roosmalen J, *et al*. Introduction of a qualitative perinatal audit at Muhimbili National Hospital, Dar es Salaam, Tanzania. *BMC Pregn Childb*. 2009;**9**:45. doi: 10.1186/1471-2393-9-45
10. Kidanto HL, Massawe SN, Nyström L, *et al*. Analysis of perinatal mortality at a teaching hospital in Dar es Salaam, Tanzania. *Afr J Reprod Health*. 2006;**10**:72-80.
11. Boo NY. Neonatal resuscitation programme in Malaysia: an eight-year experience. *Singapore Med J*. 2009;**50**:152-9.
12. Hole MK, Olmsted K, Kiromera A, *et al*. Neonatal Resuscitation Curriculum in Malawi, Africa: Did It Change In-Hospital Mortality? *Int J Pediatr*. 2012;**2012**:408689. doi: 10.1155/2012/408689
13. Conde-Agudelo A, Belizán JM, Diaz-Rossello J. Kangaroo mother care to reduce morbidity and mortality in low birthweight infants. *Cochrane Database Syst Rev*. 2011;**3**:CD002771. doi: 10.1002/14651858.CD002771.pub2.
14. Mwansa-Kambafwile J, Cousens S, Hansen T, *et al*. Antenatal steroids in preterm labour for the prevention of neonatal deaths due to complications of preterm birth. *Int J Epidemiol*. 2010;**39**(Supp 1):i122–33. doi: 10.1093/ije/dyq029.
15. Ho JJ, Subramaniam P, Henderson-Smart DJ, *et al*. Continuous distending pressure for respiratory distress syndrome in preterm infants. *Cochrane Database Syst Rev*. 2002;**2**:CD002271.

For peer review only - http://bmjopen.bmj.com/site/about/guidelines.xhtml

16. Soll R, Özek E. Prophylactic animal derived surfactant extract for preventing morbidity and mortality in preterm infants. *Cochrane Database Syst Rev*. 2002;**4**:CD000511.
17. Aminu M, Unkels R, Mdegela M, *et al*. Causes of and factors associated with stillbirth in low- and middle-income countries: a systematic literature review. *BJOG*. 2014;**121**(Supp 4):141-53. doi: 10.1111/1471-0528.12995.
18. Lawn J, Shibuya K, Stein C. No cry at birth: Global estimates of intrapartum stillbirths and intrapartum-related neonatal deaths. *Bull World Health Organ*. 2005;**83**:409-17.
19. Lee ACC, Cousens S, Wall SN, *et al*. Neonatal resuscitation and immediate newborn assessment and stimulation for the prevention of neonatal deaths. *BMC Public Health*. 2011;**11**(Supp 3):S12. doi: 10.1186/1471-2458-11-S3-S12
20. Genest DR, Singer DB. Estimating the time of death in stillborn fetuses: III. External fetal examination; a study of 86 stillborns. *Obstet Gynecol*. 1992;**80**:593-600.
21. Gold KJ, Abdul-Mumin AS, Boggs ME, *et al*. Assessment of “fresh” versus “macerated” as accurate markers of time since intrauterine fetal demise in low income countries. *Int J Gynaecol Obstet*. 2014;**125**:223-7. doi: 10.1016/j.ijgo.2013.12.006
22. Kawaza K, Machen HE, Brown J, *et al*. Efficacy of a low-cost bubble CPAP system in treatment of respiratory distress in a neonatal ward in Malawi. *PLoS One*. 2014;**9**:e86327. doi: 10.1371/journal.pone.0086327
23. Bhutta ZA, Das JK, Bahl R, *et al*. Can available interventions end preventable deaths in mothers, newborn babies, and stillbirths, and at what cost? *Lancet*. 2014;**384**:347-70. doi: 10.1016/S0140-6736(14)60792-3
24. Oza S, Lawn LE, Hogan DR, *et al*. Neonatal cause-of-death estimates for the early and late neonatal periods for 194 countries: 2000-2013. *Bull World Health Organ*. 2015;**93**:19-28. doi: 10.2471/BLT.14.139790
25. Allanson ER, Pattinson RC. Quality-of-care audits and perinatal mortality in South Africa. *Bull World Health Organ*. 2015;**93**:424-8. doi: 10.2471/BLT.14.144683
26. Dumont A, Fournier P, Abrahamowicz M, Traoré M, Haddad S, Fraser WD. Quality of care, risk management, and technology in obstetrics to reduce hospital-based maternal mortality in Senegal and Mali (QUARITE): a cluster-randomised trial. The Lancet. 2013 2013/07/13/;382(9887):146-57

For peer review only - http://bmjopen.bmj.com/site/about/guidelines.xhtml

Figure and Table Captions

Figure 1: Cause of death for macerated stillbirths (n=142), fresh stillbirths (n=125) and early neonatal deaths (n=261)

Figure 2: Interrupted time series with overdispersion

Table 1: Demographic and clinical characteristics

Table 2: Interrupted time series analysis for selected perinatal outcomes

Supplementary Table 1: Stillbirth rate (per 1000 births), early neonatal mortality rate (per 1000 live births) and perinatal mortality rate (per 1000 births)

Supplementary Table 2: Cause of death for stillbirth and early neonatal death

Supplementary Table 3: Case fatality rates (CFR)

Supplementary Table 4: Standard of care

For peer review only

For peer review only - http://bmjopen.bmj.com/site/about/guidelines.xhtml

Table 1: Demographic and clinical characteristics

Characteristics	Fresh stillbirth n=125 (%)	Macerated stillbirth n=142 (%)	Early neonatal death n=259 (%)	Total n=526 (%)
**Referral**				
From other healthcare facility	32 (25.6)	15 (10.6)	37 (14.3)	84 (16.0)
From Traditional Birth Attendant	1 (0.8)	1 (0.7)	1 (0.4)	3 (0.6)
**Parity**				
1	35 (28.0)	48 (33.8)	129 (49.8)	212 (40.3)
2 to 4	64 (51.2)	67 (47.2)	84 (32.4)	215 (40.9)
5 or more	10 (8.0)	15 (10.6)	18 (6.9)	43 (8.2)
**Antenatal care**				
At least 1 ANC visit	107 (85.6)	129 (90.8)	234 (90.3)	470 (89.4)
4 or more ANC visits	38 (30.4)	47 (33.1)	98 (37.8)	183 (34.8)
**Type of pregnancy**				
Single pregnancy	113 (90.4)	131 (92.3)	227 (87.6)	471 (89.5)
Multiple pregnancy	2 (1.6)	5 (3.5)	14 (5.4)	21 (4.0)
**Mode of delivery**				
Spontaneous vaginal delivery	67 (53.6)	123 (86.6)	143 (55.2)	333 (63.3)
Assisted breech delivery	6 (4.8)	2 (1.4)	10 (3.9)	18 (3.4)
Instrumental delivery	4 (3.2)	1 (0.7)	3 (1.2)	8 (1.5)
Caesarean section	45 (36.0)	10 (7.0)	92 (35.5)	147 (27.9)
**Gestational age at delivery**				
<37 weeks	29 (23.2)	56 (39.4)	74 (28.6)	159 (30.2)
37-41 weeks	60 (48.0)	57 (40.1)	132 (51.0)	249 (47.3)
>41 weeks	5 (4.0)	4 (2.8)	9 (3.5)	18 (3.4)
**Baby's sex**				
Male	68 (54.4)	80 (56.3)	146 (56.4)	294 (55.9)
Female	52 (41.6)	57 (40.1)	99 (38.2)	208 (39.5)
**Birthweight**				
<1000g	1 (0.8)	16 (11.3)	9 (3.5)	26 (4.9)
1000-2499g	30 (24.0)	52 (36.6)	81 (31.3)	163 (31.0)
2500-4000g	77 (61.6)	61 (43.0)	149 (57.5)	287 (54.6)
>4000g	3 (2.4)	7 (4.9)	6 (2.3)	16 (3.0)

For peer review only

Table 2: Interrupted time series analysis for selected perinatal outcomes

Outcome	With over-dispersion			
	Intervention		Time	
	IRR (95% CI)	p-value	IRR (95% CI)	p-value
Perinatal death rate	1.17 (0.79, 1.74)	0.44	0.94 (0.89,1.00)	0.06
Stillbirth rate	1.31 (0.84,2.06)	0.23	0.93 (0.87,1.00)	**0.039**
Early neonatal death rate	0.35 (0.22,0.56)	**<0.001**	1.09 (1.01,1.17)	**0.01**
CFR for intrapartum-related hypoxia	0.77 (0.57,1.04)	0.09	0.98 (0.93,1.03)	0.40
CFR for prematurity	1.01 (0.64,1.59)	0.96	0.94 (0.88,0.99)	**0.04**
CFR for infections	2.25 (0.17,28.9)	0.54	1.04 (0.78,1.39)	0.77

CFR: Case fatality rate; IRR: Incidence risk ratio

For peer review only

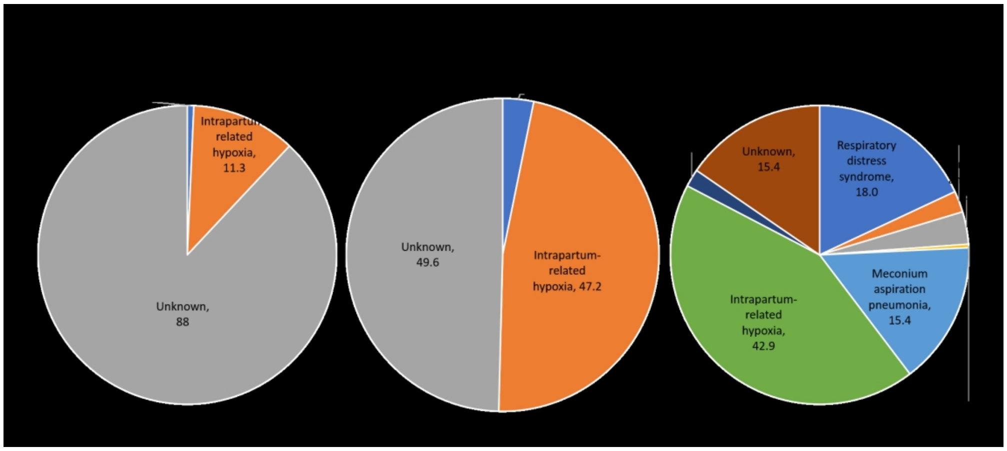

Figure 1: Cause of death for macerated stillbirths (n=142), fresh stillbirths (n=125) and early neonatal deaths (n=261)

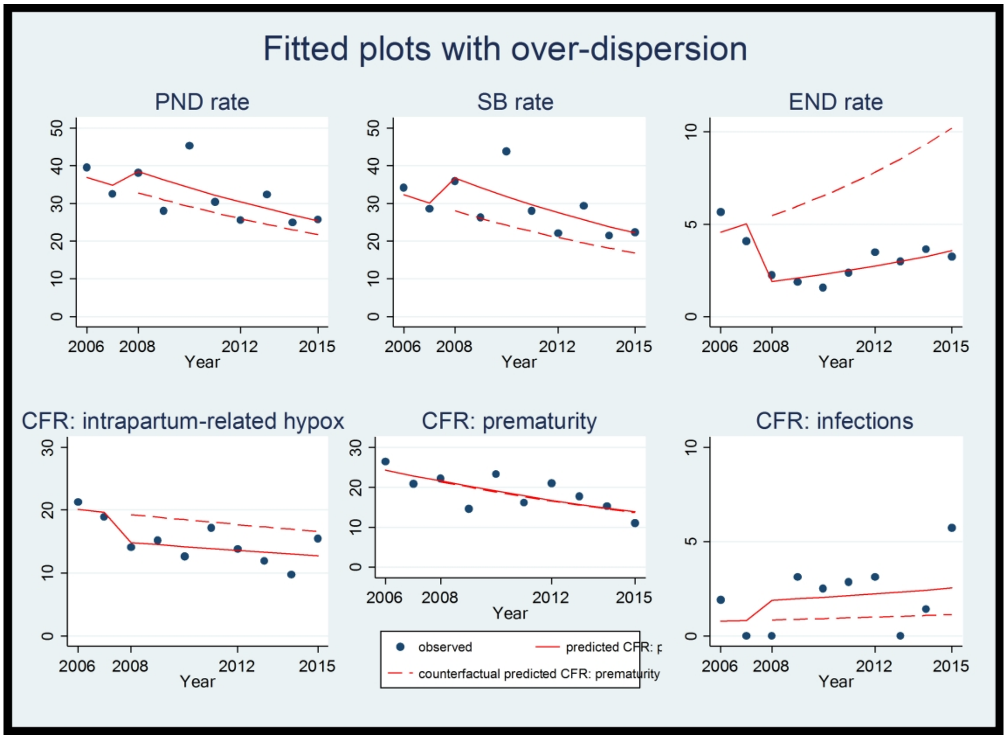

Figure 2: Interrupted time series with overdispersion

Supplementary Materials

Supplementary Table 1: Stillbirth rate (per 1000 births), early neonatal mortality rate (per 1000 live births) and perinatal mortality rate (per 1000 births).

Year	Total Births	Live Births	Stillbirth	Stillbirth Rate	Early NND	Early NND Rate	Perinatal Mortality	Perinatal Mortality Rate
2006	6468	6020	213	32.9	96	16	309	47.8
2007	7290	6881	210	28.8	193	28.1	403	55.3
2008	8386	8408	291	34.7	145	17.3	436	52.0
2009	8452	7995	207	24.5	114	14.3	321	38.0
2010	9513	8877	229	24.1	132	14.9	362	38.0
2011	8050	7610	220	27.3	166	21.8	386	47.9
2012	6732	6593	149	22.1	167	25.4	316	47.0
2013	6376	6011	183	28.7	117	19.4	300	47.0
2014	6026	5764	136	22.6	110	19	246	40.8
2015	5462	5222	120	22	112	21.5	232	42.6

For peer review only

<u>Supplementary Table 2:</u> Cause of death for stillbirth and early neonatal death.

**Cause of Death**	**Macerated Stillbirth n=142 (%)**	**Fresh Stillbirth n=124 (%)**	**Early Neonatal Death n=259 (%)**	**TOTAL n=526 (%)**
Respiratory distress syndrome	-	-	47 (18.0)	**47 (8.9)**
Infections / Septicaemia	1 (0.7)	0	6 (2.3)	**7 (1.3)**
Congenital anomaly	0	4 (3.2)	9 (3.5)	**13 (2.5)**
Meningitis	-	-	1 (0.4)	**1 (0.2)**
Meconium aspiration pneumonia	-	-	40 (15.4)	**40 (7.6)**
Intrapartum-related hypoxia	16 (11.3)	59 (47.6)	111 (42.9)	**186 (35.4)**
Haemorrhagic disease of newborn	-	-	5 (1.9)	**5 (1.0)**
Unknown	125 (88.0)	61 (49.6)	40 (15.4)	**227 (43.2)**

For peer review only

Supplementary Table 3: Case fatality rates (CFR)

Cause of death		2006	2007	2008	2009	2010	2011	2012	2013	2014	2015
**Intrapartum-related hypoxia**	Intrapartum-related hypoxia admissions	202	291	334	302	316	297	247	252	195	187
	Intrapartum-related hypoxia deaths	43	55	47	46	40	51	34	30	19	29
	**Intrapartum-related hypoxia CFR (%)**	**21.3**	**18.9**	**14.1**	**15.2**	**12.7**	**17.2**	**13.8**	**11.9**	**9.7**	**15.5**
**Prematurity**	Prematurity admissions	163	168	194	246	210	328	286	304	336	371
	Premature deaths	43	35	43	36	49	53	60	54	51	41
	**Prematurity CFR (%)**	**26.4**	**20.8**	**22.2**	**14.6**	**23.3**	**16.2**	**21.0**	**17.8**	**15.2**	**11.1**
**Infections**	Number of admissions for infections	104	149	82	128	120	140	129	171	141	87
	Deaths due to infections	2	0	0	4	3	4	4	0	2	5
	**Infections CFR (%)**	**1.9**	**0**	**0**	**3.1**	**2.5**	**2.9**	**3.1**	**0.0**	**1.4**	**5.7**

For peer review only

Supplementary Table 4: Standard of care

Standard of care	MSB (%)	FSB (%)	ENND (%)	Total (%)
Optimal	82 (67.8)	58 (49.2)	112 (45.7)	262 (53.0)
Probably acceptable	21 (17.4)	28 (23.7)	58 (23.7)	107 (21.7)
Probably sub-optimal	12 (9.9)	24 (20.3)	46 (18.8)	82 (16.6)
Sub-optimal	6 (5.0)	8 (6.8)	29 (11.8)	43 (8.7)
**Total**	**121 (100)**	**118 (100)**	**245 (100)**	**494 (100)**

For peer review only

BMJ Open

Prospective Study to Explore Changes in Quality of Care and Perinatal Outcomes After Implementation of Perinatal Death Audit in Uganda

Journal:	*BMJ Open*
Manuscript ID	bmjopen-2018-027504.R4
Article Type:	Research
Date Submitted by the Author:	15-Jan-2020
Complete List of Authors:	Kirabira, Victoria; Nsambya Hospital<br>Aminu, Mamuda; Liverpool School of Tropical Medicine, Centre for Maternal and Newborn Health ; Liverpool School of Tropical Medicine<br>Dewez, Juan; Liverpool School of Tropical Medicine, Centre for Maternal and Newborn Health<br>Byaruhanga, Romano; Nsambya Hospital<br>Okong, Pius; Nsambya Hospital<br>van den Broek, Nynke; Liverpool School of Tropical Medicine, Centre for Maternal and Newborn Health
**Primary Subject Heading**:	Obstetrics and gynaecology
Secondary Subject Heading:	Global health, Paediatrics, Public health, Reproductive medicine
Keywords:	AUDIT, Reproductive medicine < GYNAECOLOGY, Clinical audit < HEALTH SERVICES ADMINISTRATION & MANAGEMENT, Quality in health care < HEALTH SERVICES ADMINISTRATION & MANAGEMENT, stillbirth, neonatal death

SCHOLARONE™
Manuscripts

I, the Submitting Author has the right to grant and does grant on behalf of all authors of the Work (as defined in the below author licence), an exclusive licence and/or a non-exclusive licence for contributions from authors who are: i) UK Crown employees; ii) where BMJ has agreed a CC-BY licence shall apply, and/or iii) in accordance with the terms applicable for US Federal Government officers or employees acting as part of their official duties; on a worldwide, perpetual, irrevocable, royalty-free basis to BMJ Publishing Group Ltd ("BMJ") its licensees and where the relevant Journal is co-owned by BMJ to the co-owners of the Journal, to publish the Work in this journal and any other BMJ products and to exploit all rights, as set out in our licence.

The Submitting Author accepts and understands that any supply made under these terms is made by BMJ to the Submitting Author unless you are acting as an employee on behalf of your employer or a postgraduate student of an affiliated institution which is paying any applicable article publishing charge ("APC") for Open Access articles. Where the Submitting Author wishes to make the Work available on an Open Access basis (and intends to pay the relevant APC), the terms of reuse of such Open Access shall be governed by a Creative Commons licence – details of these licences and which Creative Commons licence will apply to this Work are set out in our licence referred to above.

Other than as permitted in any relevant BMJ Author's Self Archiving Policies, I confirm this Work has not been accepted for publication elsewhere, is not being considered for publication elsewhere and does not duplicate material already published. I confirm all authors consent to publication of this Work and authorise the granting of this licence.

For peer review only

Prospective Study to Explore Changes in Quality of Care and Perinatal Outcomes After Implementation of Perinatal Death Audit in Uganda

----

Victoria Nakibuuka Kirabira[a], Mamuda Aminu[b§], Juan Emmanuel Dewez[b], Romano Byaruhanga[a], Pius Okong[a], Nynke van den Broek[b]

[a] Nsambya Hospital, P.O. Box 7146, Kampala, Uganda

[b] Centre for Maternal and Newborn Health, Liverpool School of Tropical Medicine, Pembroke Place, Liverpool L3 5QA, UK

[§]Corresponding Author Contact Details:

Dr Mamuda Aminu
Centre for Maternal and Newborn Health,
Liverpool School of Tropical Medicine, Pembroke Place, Liverpool L3 5QA.
Tel: +44(0)151 705 3342
Email: Mamuda.Aminu@lstmed.ac.uk

Author email addresses:

VN Kirabira	nakibuukarv@yahoo.com
JE Dewez	manuel.dewez@lshtm.ac.uk
R Byaruhanga	byaruhangarn@yahoo.com
P Okong	puisokong@yahoo.com
NR van den Broek	Nynke.Broek@lstmed.ac.uk

Running Title: Perinatal death audit in Uganda

Keywords: perinatal death, cause of perinatal death, quality improvement

***

Word Count (Body Only): 3680

Abstract: 300

For peer review only - http://bmjopen.bmj.com/site/about/guidelines.xhtml

For peer review only

ABSTRACT

Objective: To assess the effects of perinatal death audit on perinatal outcomes in a tertiary hospital in Kampala

Design: Interrupted time series (ITS) analysis

Setting: Nsambya Hospital, Uganda

Participants: Live births and stillbirths

Interventions: Perinatal death audit

Primary and secondary outcome measures: Primary outcomes: perinatal mortality rate, stillbirth rate, early neonatal mortality rate. Secondary outcomes: case fatality rates (CFR) for asphyxia, complications of prematurity and neonatal sepsis.

Results

526 perinatal deaths were audited: 142 (27.0%) fresh stillbirths, 125 (23.8%) macerated stillbirths and 259 (49.2%) early neonatal deaths.

The ITS analysis showed a decrease in perinatal death (PND) rates without the introduction of perinatal death audits (IRR (95% CI) for time=0.94, p<0.001), but an increase in PND (IRR (95% CI) =1.17 (1.0,-1.34), p=0.0021) following the intervention. However, when over-dispersion was included in the model, there were no statistically significant differences in PND with or without the intervention (p=0.06 and p=0.44 respectively).

Stillbirth rates exhibited a similar pattern. By contrast, early neonatal death rates showed an overall upward trend without the intervention (IRR (95% CI)= 1.09 (1.01-1.17), p=0.01), but a decrease following the introduction of the perinatal death audits (IRR (95% CI)= 0.35 (0.22-0.56), p<0.001), when over-dispersion was included.

The CFR for prematurity showed a downward trend over time (IRR (95% CI) =0.94 (0.88-0.99), p=0.04) but not for the intervention. With regards CFRs for intrapartum-related hypoxia or infection, no statistically significant effect was detected for either time or the intervention.

Conclusion

The introduction of perinatal death audit showed no statistically significant effect on perinatal mortality or stillbirth rate, but a significant decrease in early neonatal mortality rate. No effect was detected on case fatality rates for prematurity, intrapartum-related hypoxia or infections. These findings should encourage more research to assess the effectiveness of perinatal death reviews on perinatal deaths in general, but also on stillbirths and neonatal deaths in particular, in low-resource settings.

For peer review only

For peer review only - http://bmjopen.bmj.com/site/about/guidelines.xhtml

Strengths and limitations of this study

- This study is one of the few studies assessing the effect of perinatal death audits in a resource-limited setting.
- The inherent limitation of the methods used to assess the cause of death was the dependence on hospital records, which were often incomplete, inaccurate or both.
- The interrupted time series design was useful to assess the effect of the intervention, but only two time points of data were documented before the introduction of the audit, which could have influenced the effect on outcomes of interest.
- Additional studies, such as ITS studies with longer pre-intervention time points of data collection, stepped-wedge randomised controlled trials, and perhaps post mortem autopsies, must confirm the findings of this study.

For peer review only

For peer review only - http://bmjopen.bmj.com/site/about/guidelines.xhtml

BACKGROUND

Currently, about 2.7 million neonatal deaths and 2.6 million stillbirths occur worldwide every year. Of these, the majority occur in low- and middle-income countries, and more than half (55%) of the global stillbirths occur in sub-Saharan Africa and South Asia [1]. Uganda's perinatal mortality is estimated to be 36 deaths per 1000 births and remains unacceptably high. Almost 50% of these are fresh stillbirths [2]. The neonatal mortality rate (21 per 1000 live births) in Uganda is also high, contributing 50% of the infant mortality rate [3]. It is crucial that interventions are put in place to reduce preventable neonatal deaths and stillbirths.

Perinatal death audit (or review) can be used to identify areas of care which can be strengthened to improve perinatal outcomes [4]. It involves reviewing cases of mortality with a view to identifying avoidable factors and implementing changes to prevent future deaths. A population-based cohort study of 943 perinatal mortalities that occurred in 90 hospitals in the Netherlands reported a decrease in substandard care (from 10% to 5%) and perinatal mortality (from 2.3 to 2.0/1000 births; $p<0.00001$) after the introduction of perinatal death audit [5].

Similarly, in a systematic review focusing on low- and middle-income countries, a meta-analysis of 7 before-and-after studies reported a 30% (95% confidence interval, 21%–38%) reduction in perinatal mortality after introduction of perinatal death audit in healthcare facilities [6]. However, the authors considered most studies included in the review as of low quality. Thus, there is a paucity of data from low-resource settings on the use of and effectiveness of perinatal death audit to improve the quality of care provided to mothers and their babies. The systematic review recommended more research in key areas, including exploring the effect of facility-based perinatal death audit on changing the quality of care [6].

The aim of this study is to assess the changes in perinatal outcomes observed after the introduction of perinatal death audit in a tertiary hospital in Uganda and to describe the changes in healthcare provided to mother and babies resulting from the perinatal death audits.

METHODS

Study setting

For peer review only - http://bmjopen.bmj.com/site/about/guidelines.xhtml

This study was conducted at Nsambya Hospital, a private not-for-profit tertiary hospital in Kampala, Uganda, which serves as a referral centre for Makindye Health Sub-district in Kampala. The facility receives 7,000 deliveries per year and provides 24-hour comprehensive emergency obstetric and newborn care services, with a staff of 6 obstetricians, 18 resident doctors, 5 intern doctors and 40 midwives.

The newborn care unit admits 2,500 babies annually and has a paediatrician/neonatologist, two residents and up to four midwives on duty per shift. It has 40 beds, including 10 neonatal intensive beds, and provides essential newborn care including; oxygen therapy, surfactant and low-cost bubble continuous positive airway pressure (CPAP), kangaroo mother care and phototherapy.

Data collection

Perinatal death audit using locally developed tools and guidelines started in 2008 and was led by a multidisciplinary audit team comprising of obstetricians, paediatricians, resident doctors, midwives and administrators. Team meetings were held weekly, chaired by the most senior obstetrician or paediatrician. Perinatal death was defined as “death of a fetus greater than a birth weight of 1000g if measured, or a gestational age of at least 28 completed weeks or more, or a body length (crown-heel) of at least 35cm or more”[7] and included all stillbirths and deaths within the first week of life (early neonatal deaths). The number of perinatal deaths was documented daily using clinical handover reports and registers in the labour ward and neonatal special care unit. Due to the large numbers of deaths, one-quarter of the cases (every 4th case) were selected for audit from each of the following categories: fresh stillbirths, macerated stillbirths and early neonatal deaths.

The number of admissions in the newborn unit, reasons for admission, death and case fatalities rates for intrapartum-related hypoxia, infection and prematurity were collected for the period before the perinatal audit was introduced (2006-2007) and subsequently for the period after commencement of the perinatal death audit (2008-2015). The data was obtained and aggregated monthly through a retrospective analysis of registers and patient records. Numbers of live and total births were also documented to enable rates calculation.

Intervention: the audit process

For each case selected for review, an audit form was completed by a resident doctor, who also provided a narrative summary for each case. Sources of information included: clinical handover books, maternity

For peer review only - http://bmjopen.bmj.com/site/about/guidelines.xhtml

registers, newborn admission register, and clinical notes for mothers and newborns. Each case was then presented to the rest of the audit team for detailed discussion. The information presented to the team allowed reviewing the cause of death and assigning a more precise cause of death by consensus. Care provided was judged against existing local protocols and national guidelines. No clinical autopsies were performed due to lack of a full-time pathologist at the hospital.

The quality of care provided at the hospital was also graded by consensus and classified into four categories:

- Optimal care generally accepted practice and all standards of care were followed.
- Suboptimal care but different management would have made no difference to the outcome (probably acceptable)
- Suboptimal care where different management might have made a difference to the outcome - an avoidable factor of uncertain clarity or influence on the outcome (probably suboptimal)
- Different management would reasonably be expected to have made a difference to the outcome. A clearly avoidable factor implying that the adverse outcome could have been prevented) (suboptimal)

The overall grading of the care provided to the mother and baby, the identified specific areas of suboptimal care and the recommendations for improvement were documented. This grading included referred mothers as well, whereas there were delays from the referring site, suboptimal care can also occur in tertiary hospital as well, such as delay in performing a Caesarean section

The summary of each case included a list of the actions to be taken. In each case, a specific person was appointed to lead the implementation of the relevant actions. During monthly meetings, implementation progress was compared with the action plan by the wider audit team. A number of interventions, such as neonatal resuscitation training, the introduction of Kangaroo Mother Care (KMC), building of a new maternity theatre, were thus introduced gradually over the years to address the gaps in care. Whereas maternal death reviews had been introduced before the perinatal death audits, they emphasized more care to the mother than the baby.

Data analysis

For peer review only - http://bmjopen.bmj.com/site/about/guidelines.xhtml

For peer review only

SPSS© (IBM, version 22) was used for descriptive analyses. Stillbirth and perinatal mortality rates were expressed per 1000 total births, while the early neonatal mortality rate was expressed per 1000 live births. Case fatality rate (CFR) was defined as the proportion of deaths among cases diagnosed with the disease. Statistical significance was calculated at 95% confidence interval or p-value of <0.05.

Data for the six primary and secondary outcomes were each analysed using interrupted time series (ITS) models in Stata©, following Cochrane checklist for assessing the quality of ITS studies as a guide. Poisson regression models of counts were fitted with covariates for intervention and year, and the relevant population size (log-transformed) used as the offset. As proposed by Bernal et al [8] over-dispersion was considered (by using an additional option: scale(x2)) since it often arises in the context of count data. Auto-correlation was also inspected using plots. The 5% level was used to determine statistical significance.

Since low birth weight and prematurity are both associated with a higher risk of perinatal mortality, a one-way between-groups analysis of variance (ANOVA) was conducted to explore changes in birth weight and gestational age over time.

A narrative summary description was used to report actions agreed and implemented following an audit. These were documented chronologically as derived from minutes of the audit meetings.

Ethical approval

Approval for the study was obtained from the Nsambya Hospital Ethical Review Committee.

Patient and public involvement

Individual patient consent was not applicable as the research team extracted anonymized data from case notes. Patients and the public were not involved in this study.

RESULTS

Demographic and clinical characteristics:

A total of 58,997 births and 2,616 perinatal deaths occurred between 2008 and 2015 (Supplementary Table 1). Of these, 603 (23%) perinatal deaths were selected for audit. After review, 526 were found to

For peer review only - http://bmjopen.bmj.com/site/about/guidelines.xhtml

meet the definition of perinatal mortality: 142 (27.0%) fresh stillbirths, 125 (23.8%) macerated stillbirths and 259 (49.2%) early neonatal deaths (Table 1).

The majority of cases came to the hospital directly, with 17% referrals. Mothers had a mean age of 27.3 years (SD: 5.1); median parity of 2 (SD of 1.6). Just over one-third of fresh stillbirths and early neonatal deaths were delivered by Caesarean section. The mean birth weight of babies was 2610 grams (SD: 969.4). Between 23.2% and 39.4% were born prematurely (Table 1).

Cause of death:

Overall, intrapartum-related hypoxia was the leading cause of perinatal mortality in this setting, accounting for 35.4% of all cases (Figure 1 and Supplementary Table 2). This is followed by respiratory distress syndrome (8.9%) and meconium aspiration pneumonia (7.6%). Cause of death in 43.2% could not be ascertained: 15.4% for neonatal deaths, 49.2% for fresh stillbirths and 88.0% for macerated stillbirths. However, the proportion of deaths for which cause of death could not be established ("cause unknown") decreased from 34.1% (overall) in 2008 to 2.7% in 2013.

Trend in perinatal mortality rate:

The ITS analysis showed that, without modelling over-dispersion, there would have been a downward trend (a decrease) in perinatal death rates (Incidence risk ratio (IRR) (95% CI) for time=0.94, p<0.001), while a statistically significant increase (IRR (95% CI) =1.17 (1.0,-1.34), p=0.0021) was observed following the introduction of perinatal death audits. However, when over-dispersion was included in the model, both effects were not statistically significant (Table 2, Figure 2, and Supplementary Table 3).

Stillbirth rates exhibited a similar pattern. By contrast, early neonatal death rates showed an overall upward trend (IRR (95% CI)= 1.09 (1.01-1.17), p=0.01) but a a decrease following the introduction of the perinatal death audits (IRR (95% CI) = 0.35 (0.22-0.56), p<0.001) when over-dispersion was included in the model (Table 2, and Figures 2). The ANOVA to explore changes in birth weight and gestational age over time showed no statistically significant change in birthweight over time (p = 0.872), and no change in gestational age at birth over time (p = 0.219).

The CFR for prematurity showed a downward trend over time (IRR (95% CI)=0.94 (0.88-0.99), p=0.04) but a downward trend was not observed with the intervention. With regards to the CFRs for intrapartum-

For peer review only - http://bmjopen.bmj.com/site/about/guidelines.xhtml

For peer review only

related hypoxia and infections, no statistically significant effect was detected with or without the intervention (Table 2, Figures 2, and Supplementary Table 3).

For each outcome considered, the autocorrelation and partial correlation plots provided no evidence that there was autocorrelation not accounted for in the models used.

Quality improvement:

More than half of the cases (53%) were deemed to have had optimal care. The rest had probably acceptable care (21.7%), probably sub-optimal care (16.6%) or sub-optimal care (8.7%) (Supplementary Table 4). Among the different types of mortalities, sub-optimal care was most common among early neonatal deaths (11.8%), as compared to fresh stillbirth (6.8%) and macerated stillbirth (5.0%).

DISCUSSION

Main findings

This study assessed the effect of perinatal death audit to improve the quality of perinatal care and perinatal outcomes in a tertiary hospital in Uganda over a period of 10 years.

Intrapartum-related hypoxia was the leading cause of perinatal mortality (accounting for 35.4%), followed by respiratory distress syndrome (8.9%) and meconium aspiration pneumonia (7.6%). Cause of death was unknown in 43.2% of cases. The proportion of deaths with unknown cause decreased from 34.1% (overall) in 2008 to 2.7% in 2013.

There was no effect of perinatal death audit on perinatal mortality or stillbirth rates. However, the intervention led to a decrease in neonatal mortality rate [IRR (95% CI)= 0.35 (0.22-0.56), p<0.001)]. It was noted that the high numbers of stillbirth inflated the numbers of perinatal death. Thus, even though the audit made a difference to early neonatal deaths, this difference is neutralised when combined with stillbirths.

The CFR for prematurity showed a downward trend over time (IRR (95% CI)=0.94 (0.88-0.99), p=0.04), but this was not related to the perinatal death audits. The CFRs for intrapartum-related hypoxia and infections were not affected by the intervention.

For peer review only - http://bmjopen.bmj.com/site/about/guidelines.xhtml

More than half of the cases (53%) were deemed to have had optimal care, with the rest of the cases having varying levels of acceptability. Early neonatal deaths had the highest proportion of cases with suboptimal care (11.8%). Case fatality rates decreased between 2006 and 2015 for intrapartum-related hypoxia (21.3% to 15.5%) and for complications of prematurity (26.4% to 11.1%). However, case fatality rates for infection increased from 1.9% in 2006 to 5.7% in 2015.

Quality improvement

In 2007 and 2008, the perinatal death review team observed many cases of delay between the decision for, and the actual conduct of, Caesarean Section, which was associated with high numbers of intrapartum-related hypoxia. To reduce the time taken to prepare mothers for the procedure, the team recommended the involvement of doctors in the preparation. These ensured mothers were catheterized and blood samples were taken for grouping and cross-matching at the time of decision. During the same period, a new dedicated operating theatre was built close to the maternity ward.

In 2008, two major factors contributing to perinatal mortality were identified to have recurred consistently: poor neonatal resuscitation and poor use of the partographs. Both of these were thought to contribute to perinatal death due to intrapartum-related hypoxia. To tackle the problem of poor resuscitation skills, maternity staff were re-trained in resuscitation and mentored by the most senior staff. In addition, senior staff were involved earlier in deliveries among high-risk patients. The hospital also acquired more resuscitation equipment, including dedicated Ambu bags, masks and Resuscitaires for the labour ward and theatre. Midwives were re-trained in the use of the partograph. Human resource challenges were addressed with more midwives assigned to the maternity unit.

Training in neonatal resuscitation was done on a three-monthly basis. In 2007, asphyxiated babies were only given 10% dextrose. In subsequent years after the training, the care was expanded to include providing warmth, stimulation and ventilation with Ambu bag and provision of oxygen. This was implemented both in the labour wards and theatre.

In 2009, the case fatality rate for complications of prematurity was observed to begin to rise (Figure 2). This prompted action to address the problem of hypothermia, which was observed to be related to many of the cases. The team introduced Kangaroo Mother Care (KMC) for stable preterm babies. They also recommended the administration of dexamethasone to mothers at risk of preterm birth, and use of radiant warmers in the theatre, labour room and the newborn unit to improve thermal control. For babies

For peer review only - http://bmjopen.bmj.com/site/about/guidelines.xhtml

with respiratory distress, Continuous Positive Airway Pressure (CPAP) was introduced, and rescue surfactant was made available for use in 2014.

In 2012, light-emitting diode phototherapy was introduced for the management of babies with neonatal jaundice. No additional specific actions to improve the management of neonatal infections were documented as this was not considered a major cause of mortality when compared to intrapartum-related hypoxia and complications of prematurity. However, all interventions were observed to be sustained throughout the period under study. This study assessed the introduction of perinatal audits in a low resource setting. There are few studies exploring the use of perinatal death audit to improve care in low-resource settings. In a similar study of 305 perinatal deaths in Tanzania, suboptimal care was identified in 80% of the cases. However, there was no report of changes implemented or on the effect of the changes [9].

Strengths and limitations

This study is one of the few studies assessing the effect of perinatal death audits in a resource-limited setting, over a period of ten years. While it is difficult to attribute all changes in perinatal outcomes to perinatal death audit alone, the process has helped to identify areas of healthcare that need support for improvement in outcomes.

The results should be interpreted in light of the limitations. The attribution of cause of death was based on hospital records, which were often incomplete, inaccurate or both. In terms of the ITS design, there were only two timepoints of data before the introduction of the audits, which limits the capacity of the analysis to examine evidence of a change in slope with the adoption of the intervention. A third timepoint, as suggested in the Cochrane standards for ITS, would have addressed this issue.

Implications for future practice and research

Problems identified through audit:

Perinatal death audit was integrated as part of routine care to help identify deficiencies in the clinical care pathway and to formulate priority actions to ensure improved quality of care and reduced loss of life. Poor use of the partograph and fetal heart monitoring, delay in performing Caesarean sections when these were indicated, poor neonatal resuscitation skills and poor management of preterm babies, were

For peer review only - http://bmjopen.bmj.com/site/about/guidelines.xhtml

identified as areas requiring improvement in the quality of care. In addition, there was evidence of significant delays incurred when a referral from a lower level healthcare facility occurred. These findings are similar to a study from Tanzania where 47% of perinatal deaths audited had poor fetal heart monitoring, and 23% had delays in performing Caesarean section [9,10].

The interventions for improvement included: training in the use of the partograph, fetal heart monitoring, neonatal resuscitation, measures to reduce delay in performing a Caesarean section by involving doctors in the timely preparation of mothers before transfer to the theatre and the building of a separate maternity theatre. The implementation of these interventions was monitored during the weekly audit meetings. Training in neonatal resuscitation has been conducted in Malaysia and Malawi with similar success in the acquisition of skills [11,12].

<u>Interventions for prematurity-related complications:</u>

The interventions implemented included: reduction of hypothermia through the provision of radiant warmers and incubators for the critically-ill babies in the labour wards, theatre and the newborn unit. KMC and immediate skin-to-skin care at birth were initiated in 2009 and 2012, respectively. The use of skin-to-skin care was noted to improve from 40% to 80% of live births after repeated training. KMC has been adopted and is used for all stable low birth weight babies in the newborn unit. A Cochrane systematic review has established that KMC is associated with reduced risk of neonatal mortality (by 51%), nosocomial infection or sepsis (by 58%) and hypothermia (by 77%) [13]. The interventions for reduction of respiratory distress included: use of antenatal steroids, bubble CPAP and use of rescue surfactant. Antenatal steroids, bubble CPAP and rescue surfactant have been found to have low coverage in low-resource settings despite their potential to reduce mortality by 48%, 31% and 32%, respectively [14,15,16]. The majority (98%) of the preterm babies who needed it were able to pay for it and actually received it. This is a private not for profit hospital, majority of the patients that are admitted are able to afford the rates offered by the hospital. Low-cost surfactant was obtained from South Africa / India which cost about 200 dollars per vial. Surfactant is a product that is paid for in our hospital just like other commodities like antibiotics or Caesarean section. The cost of surfactant is less than an operation like Caesarean section.

<u>Intrapartum-related hypoxia and prematurity-related deaths:</u>

For peer review only - http://bmjopen.bmj.com/site/about/guidelines.xhtml

In this study, the case fatality rate for intrapartum-related hypoxia was reduced from 21.3% to 15.5%. For stillbirth, intrapartum-related hypoxia has been reported to account for between 3.1% and 25% of all cases of stillbirths in low- and middle-income countries [17]. The change may be attributable to the improvement in resuscitation practices, following training and provision of equipment. Training in neonatal resuscitation has been reported to reduce deaths in babies with intrapartum-related hypoxia by 30% and early neonatal deaths by 38% [18,19]. An analysis of seven facility-based studies estimated that training in neonatal resuscitation reduced the neonatal mortality rate from 43% to 17%. However, a study in Malawi reported that resuscitation training did not reduce mortality [12]. In Malawi, factors such as availability and replacement of resuscitation equipment and mentorship after training were not part of the improvement package. Additionally, in our study, the training was repeated on a three-monthly basis and continuous feedback and mentoring were provided which helped ensure continuous improvement in quality. Although these interventions had already been known to work, audit acted as a precursor to implement them.

It is noteworthy that intrapartum-related hypoxia was documented as a cause of death even for macerated stillbirth. Since maceration could set in only six hours post-mortem [20], it is not uncommon in low-resource settings to find a macerated intrapartum death due to delays in an intervention during labour. It could also be because healthcare providers often misclassify fresh stillbirth as macerated stillbirth [21].

Other factors that might have contributed to the reduction in case fatality rates were partograph use, improved fetal heart monitoring and having a dedicated maternity theatre to reduce waiting times for Caesarean section.

The case fatality rate for prematurity was reduced by more than half. One of the major interventions was the introduction of KMC, which has been shown to reduce mortality and morbidity in low-resource settings by 40% and 34%, respectively [13]. Moreover, CPAP was also introduced, which has been reported to improve survival of preterm babies by 27% among resource-limited settings [22]. Finally, in 2014 surfactant administration was introduced, which may have further reduced the mortality in prematurity-related deaths. Although surfactant is not yet provided in many developing countries as demonstrated in this study, it can be given safely under the supervision of experienced personnel.

For peer review only - http://bmjopen.bmj.com/site/about/guidelines.xhtml

This study suggests that a significant reduction in case fatality rate for intrapartum-related hypoxia can be expected to occur after the implementation of multiple interventions to improve the inpatient care of small and sick newborns. It further confirms the findings of a Delphi study that estimated supportive care in a Special Care Baby Unit could avert 70% of neonatal deaths due to preterm birth complications, and that 90% could be averted with the availability of Neonatal Intensive Care Units (NICUs) [23].

The fact that only 2.3% of early neonatal deaths were attributed to infection is a surprising finding. In another study, infections accounted for around 14% of early neonatal deaths [24]. Infection is commonest in the lowest socio-economic groups and this could be because most cases of neonatal sepsis were late neonatal deaths (after 1 week) and so would be missed by the audit. Furthermore, 15.4% of early neonatal deaths had an unknown cause of death. It is possible that this represents an underestimation of the proportion of deaths attributable to infection. In addition, some cases of respiratory distress syndrome may actually have been cases of neonatal infection, as it is often very difficult to differentiate between the two conditions without sophisticated diagnostic tests.

The relatively large sample size in this study provided a good opportunity to document the distribution of the causes and factors contributing to perinatal mortality. Nevertheless, in 43% of the deaths, a cause of death could not be established. This proportion was highest for macerated stillbirths (88% unknown) with 49% of fresh stillbirths and 15% of early neonatal deaths assigned to the category “unknown”. This may be related to the availability of information about the deaths: the longer the baby died before birth, the more difficult it becomes to obtain information about the circumstances of the death. Furthermore, diagnosis is easier for newborns than for stillbirths since clinicians usually have more time to examine and monitor a sick newborn baby during the period of admission and before death. Another possible reason for the inability to establish the cause of death may be the lack of diagnostic capacity, relating to both staff and equipment. To reduce the proportion of deaths with an unknown cause in low-resource settings, health care providers should keep clinical records as complete and as accurate as possible.

<u>Effectiveness of perinatal death audit:</u>

Ideally, the results of this study should be confirmed by randomised controlled trials conducted in the same context of care.

For peer review only - http://bmjopen.bmj.com/site/about/guidelines.xhtml

Other reports found that perinatal death audit did not improve perinatal outcomes. In a five-year study of health facilities in South Africa, it was observed that among the 54 health facilities that started perinatal death audit, only 30% had a decrease in perinatal mortality, while 35% had an increase [25]. The study identified several facility-related factors that contributed to the increase in perinatal mortality, including lack of use of antenatal steroids, lack of nursing personnel, fetal distress not detected during the antepartum period and incorrect use of the partograph. Additional ITS studies with longer pre-intervention time points of data collection in low and middle-income countries should confirm or refute our findings understanding of the effects of this intervention in these settings. Finally, randomised controlled trials using a stepped wedge design, are needed to assess the effect of perinatal death audit on perinatal outcomes.

A systematic review and meta-analysis of the effect of perinatal audits in low-resource settings showed a reduction of perinatal deaths of 30% attributable to this intervention [6]. A large randomized controlled trial conducted in Mali and Senegal which aimed to assess the effect of a complex intervention (including maternal death audits) on maternal and perinatal outcomes, showed that maternal audits reduced ENMR by 24%, while there was no effect on SBR [26].These findings on perinatal outcomes are in line with those of our study, even though the intervention was different (we assessed perinatal death audits, while the RCT assessed maternal death audits). This suggests that although perinatal death audits seem promising in reducing perinatal mortality, the role of these audits on SBR and ENMR remains unclear.

CONCLUSION

This study highlights the potential of perinatal death review to identify how care pathways can be improved over time to reduce adverse perinatal outcomes in low-resource settings. This study shows that the introduction of perinatal death audit allowed for the identification of the main causes of death and contributing factors, and prioritise areas in the provision of healthcare that need improvement. However, although the intervention showed a decrease in early neonatal death, the perinatal audit process did not have an impact on PMR and SBR. These findings should encourage more healthcare providers to undertake research on perinatal death reviews, especially in low-resource settings to assess the effectiveness of perinatal death audit on perinatal deaths in general, but also on stillbirths and neonatal deaths in particular.

For peer review only - http://bmjopen.bmj.com/site/about/guidelines.xhtml

DECLARATIONS

Ethical approval and consent to publish

Approval for the study was obtained from the Nsambya Hospital Ethical Review Committee. Individual patient consent was not applicable as the research team extracted anonymized data from case notes.

Acknowledgements

We would like to thank all healthcare providers who took part in the review process. We particularly appreciate the contributions of Dr. Musana Othniel, Dr. Sebunya Robert, Dr. Nazziwa Rita, Dr. Catherine Nyagabyaki, Sr. Nabwami Immaculate, Dr. Tumwine Gilbert and Dr. Zaake.

We are also grateful to Robert Lloyd for his contribution to data processing, to Dr Sarah White for help with part of the statistical analysis, to Prof. Mathews Mathai for reviewing the manuscript and to Caroline Hercod for editing the manuscript.

Disclosure of Interests

All authors declare no competing interests.

Funding

No funding was received for the data collection. However, the cost of data processing, analysis and publication was sponsored by the 'Making it Happen' Programme funded by DFID/UKAid.

Availability of data and materials

The datasets used and/or analysed during the current study are available from the corresponding author upon reasonable request.

Contributions to Authorship

VN contributed to study design, data collection, analysis and writing up. MA processed and analyzed the data and contributed to all drafts of the paper. JED contributed to the conception of the paper, to the analysis, and writing of the paper. RB took part in writing the study proposal, data collection and review

For peer review only - http://bmjopen.bmj.com/site/about/guidelines.xhtml

of cases. PO contributed to the study proposal, data collection and case reviews. NVDB oversaw the conception and analysis and contributed to the initial and final drafts of the paper.

REFERENCES

1. Lawn JE, Blencowe H, Pattinson R, *et al*. Stillbirths: Where? When? Why? How to make the data count? *Lancet*. 2011;**377**:1448-63. doi: 10.1016/S0140-6736(10)62187-3
2. Uganda Bureau of Statistics. Uganda Demographic and Health Survey 2011. Uganda Bureau of Statistics: Kampala 2011. https://dhsprogram.com/pubs/pdf/fr264/fr264.pdf
3. Uganda Ministry of Health, Reproductive Health Division. Annual Maternal and Perinatal Death Review Report 2013/2014. Ministry of Health: Kampala, 2014.
4. Buchmann EJ. Towards greater effectiveness of perinatal death audit in low- and middle-income countries. *BJOG*. 2014;**121**(Supp 4):S134-6. doi: 10.1111/1471-0528.12904
5. Eskes M, Waelput AJM, Erwich JJHM, *et al*. Term perinatal mortality audit in the Netherlands 2010–2012: a population based cohort study. *BMJ Open*. 2014;**4**:e005652. doi: 10.1136/bmjopen-2014-005652
6. Pattinson R, Keber K, Waiswa P, *et al*. Perinatal Mortality Audit: Counting, accountability, and overcoming challenges in scaling up in low- and middle-income countries. *Int J Gynaecol Obstet*. 2009;**109**(Supp 1):113-22. doi: 10.1016/j.ijgo.2009.07.011.
7. World Health Organization. ICD-10 International statistical classification of diseases and related health problems. World Health Organization: Geneva, 2004.
8. Bernal JL, Cummins S, Gasparrini A. Interrupted time series regression for the evaluation of public health interventions: a tutorial. *Int J Epidemiol.* 2017;**46**:348-55 doi: 10.1093/ije/dyw098
9. Kidanto HL, Mogren I, van Roosmalen J, *et al*. Introduction of a qualitative perinatal audit at Muhimbili National Hospital, Dar es Salaam, Tanzania. *BMC Pregn Childb*. 2009;**9**:45. doi: 10.1186/1471-2393-9-45
10. Kidanto HL, Massawe SN, Nyström L, *et al*. Analysis of perinatal mortality at a teaching hospital in Dar es Salaam, Tanzania. *Afr J Reprod Health*. 2006;**10**:72-80.
11. Boo NY. Neonatal resuscitation programme in Malaysia: an eight-year experience. *Singapore Med J*. 2009;**50**:152-9.
12. Hole MK, Olmsted K, Kiromera A, *et al*. Neonatal Resuscitation Curriculum in Malawi, Africa: Did It Change In-Hospital Mortality? *Int J Pediatr*. 2012;**2012**:408689. doi: 10.1155/2012/408689
13. Conde-Agudelo A, Belizán JM, Diaz-Rossello J. Kangaroo mother care to reduce morbidity and mortality in low birthweight infants. *Cochrane Database Syst Rev*. 2011;**3**:CD002771. doi: 10.1002/14651858.CD002771.pub2.
14. Mwansa-Kambafwile J, Cousens S, Hansen T, *et al*. Antenatal steroids in preterm labour for the prevention of neonatal deaths due to complications of preterm birth. *Int J Epidemiol*. 2010;**39**(Supp 1):i122–33. doi: 10.1093/ije/dyq029.
15. Ho JJ, Subramaniam P, Henderson-Smart DJ, *et al*. Continuous distending pressure for respiratory distress syndrome in preterm infants. *Cochrane Database Syst Rev*. 2002;**2**:CD002271.

For peer review only - http://bmjopen.bmj.com/site/about/guidelines.xhtml

16. Soll R, Özek E. Prophylactic animal derived surfactant extract for preventing morbidity and mortality in preterm infants. *Cochrane Database Syst Rev*. 2002;**4**:CD000511.
17. Aminu M, Unkels R, Mdegela M, *et al*. Causes of and factors associated with stillbirth in low- and middle-income countries: a systematic literature review. *BJOG*. 2014;**121**(Supp 4):141-53. doi: 10.1111/1471-0528.12995.
18. Lawn J, Shibuya K, Stein C. No cry at birth: Global estimates of intrapartum stillbirths and intrapartum-related neonatal deaths. *Bull World Health Organ*. 2005;**83**:409-17.
19. Lee ACC, Cousens S, Wall SN, *et al*. Neonatal resuscitation and immediate newborn assessment and stimulation for the prevention of neonatal deaths. *BMC Public Health*. 2011;**11**(Supp 3):S12. doi: 10.1186/1471-2458-11-S3-S12
20. Genest DR, Singer DB. Estimating the time of death in stillborn fetuses: III. External fetal examination; a study of 86 stillborns. *Obstet Gynecol*. 1992;**80**:593-600.
21. Gold KJ, Abdul-Mumin AS, Boggs ME, *et al*. Assessment of "fresh" versus "macerated" as accurate markers of time since intrauterine fetal demise in low income countries. *Int J Gynaecol Obstet*. 2014;**125**:223-7. doi: 10.1016/j.ijgo.2013.12.006
22. Kawaza K, Machen HE, Brown J, *et al*. Efficacy of a low-cost bubble CPAP system in treatment of respiratory distress in a neonatal ward in Malawi. *PLoS One*. 2014;**9**:e86327. doi: 10.1371/journal.pone.0086327
23. Bhutta ZA, Das JK, Bahl R, *et al*. Can available interventions end preventable deaths in mothers, newborn babies, and stillbirths, and at what cost? *Lancet*. 2014;**384**:347-70. doi: 10.1016/S0140-6736(14)60792-3
24. Oza S, Lawn LE, Hogan DR, *et al*. Neonatal cause-of-death estimates for the early and late neonatal periods for 194 countries: 2000-2013. *Bull World Health Organ*. 2015;**93**:19-28. doi: 10.2471/BLT.14.139790
25. Allanson ER, Pattinson RC. Quality-of-care audits and perinatal mortality in South Africa. *Bull World Health Organ*. 2015;**93**:424-8. doi: 10.2471/BLT.14.144683
26. Dumont A, Fournier P, Abrahamowicz M, Traoré M, Haddad S, Fraser WD. Quality of care, risk management, and technology in obstetrics to reduce hospital-based maternal mortality in Senegal and Mali (QUARITE): a cluster-randomised trial. The Lancet. 2013 2013/07/13/;382(9887):146-57

For peer review only

For peer review only - http://bmjopen.bmj.com/site/about/guidelines.xhtml

Figure and Table Captions

Figure 1: Cause of death for macerated stillbirths (n=142), fresh stillbirths (n=125) and early neonatal deaths (n=261)

Figure 2: Interrupted time series with overdispersion

Table 1: Demographic and clinical characteristics

Table 2: Interrupted time series analysis for selected perinatal outcomes

Supplementary Table 1: Stillbirth rate (per 1000 births), early neonatal mortality rate (per 1000 live births) and perinatal mortality rate (per 1000 births)

Supplementary Table 2: Cause of death for stillbirth and early neonatal death

Supplementary Table 3: Case fatality rates (CFR)

Supplementary Table 4: Standard of care

For peer review only

For peer review only - http://bmjopen.bmj.com/site/about/guidelines.xhtml

Table 1: Demographic and clinical characteristics

Characteristics	Fresh stillbirth n=125 (%)	Macerated stillbirth n=142 (%)	Early neonatal death n=259 (%)	Total n=526 (%)
**Referral**				
From other healthcare facility	32 (25.6)	15 (10.6)	37 (14.3)	84 (16.0)
From Traditional Birth Attendant	1 (0.8)	1 (0.7)	1 (0.4)	3 (0.6)
**Parity**				
1	35 (28.0)	48 (33.8)	129 (49.8)	212 (40.3)
2 to 4	64 (51.2)	67 (47.2)	84 (32.4)	215 (40.9)
5 or more	10 (8.0)	15 (10.6)	18 (6.9)	43 (8.2)
**Antenatal care**				
At least 1 ANC visit	107 (85.6)	129 (90.8)	234 (90.3)	470 (89.4)
4 or more ANC visits	38 (30.4)	47 (33.1)	98 (37.8)	183 (34.8)
**Type of pregnancy**				
Single pregnancy	113 (90.4)	131 (92.3)	227 (87.6)	471 (89.5)
Multiple pregnancy	2 (1.6)	5 (3.5)	14 (5.4)	21 (4.0)
**Mode of delivery**				
Spontaneous vaginal delivery	67 (53.6)	123 (86.6)	143 (55.2)	333 (63.3)
Assisted breech delivery	6 (4.8)	2 (1.4)	10 (3.9)	18 (3.4)
Instrumental delivery	4 (3.2)	1 (0.7)	3 (1.2)	8 (1.5)
Caesarean section	45 (36.0)	10 (7.0)	92 (35.5)	147 (27.9)
**Gestational age at delivery**				
<37 weeks	29 (23.2)	56 (39.4)	74 (28.6)	159 (30.2)
37-41 weeks	60 (48.0)	57 (40.1)	132 (51.0)	249 (47.3)
>41 weeks	5 (4.0)	4 (2.8)	9 (3.5)	18 (3.4)
**Baby's sex**				
Male	68 (54.4)	80 (56.3)	146 (56.4)	294 (55.9)
Female	52 (41.6)	57 (40.1)	99 (38.2)	208 (39.5)
**Birthweight**				
<1000g	1 (0.8)	16 (11.3)	9 (3.5)	26 (4.9)
1000-2499g	30 (24.0)	52 (36.6)	81 (31.3)	163 (31.0)
2500-4000g	77 (61.6)	61 (43.0)	149 (57.5)	287 (54.6)
>4000g	3 (2.4)	7 (4.9)	6 (2.3)	16 (3.0)

For peer review only - http://bmjopen.bmj.com/site/about/guidelines.xhtml

Table 2: Interrupted time series analysis for selected perinatal outcomes

Outcome	With over-dispersion			
	Intervention		Time	
	IRR (95% CI)	p-value	IRR (95% CI)	p-value
Perinatal death rate	1.17 (0.79, 1.74)	0.44	0.94 (0.89,1.00)	0.06
Stillbirth rate	1.31 (0.84,2.06)	0.23	0.93 (0.87,1.00)	**0.039**
Early neonatal death rate	0.35 (0.22,0.56)	**<0.001**	1.09 (1.01,1.17)	**0.01**
CFR for intrapartum-related hypoxia	0.77 (0.57,1.04)	0.09	0.98 (0.93,1.03)	0.40
CFR for prematurity	1.01 (0.64,1.59)	0.96	0.94 (0.88,0.99)	**0.04**
CFR for infections	2.25 (0.17,28.9)	0.54	1.04 (0.78,1.39)	0.77

CFR: Case fatality rate; IRR: Incidence risk ratio

For peer review only

For peer review only - http://bmjopen.bmj.com/site/about/guidelines.xhtml

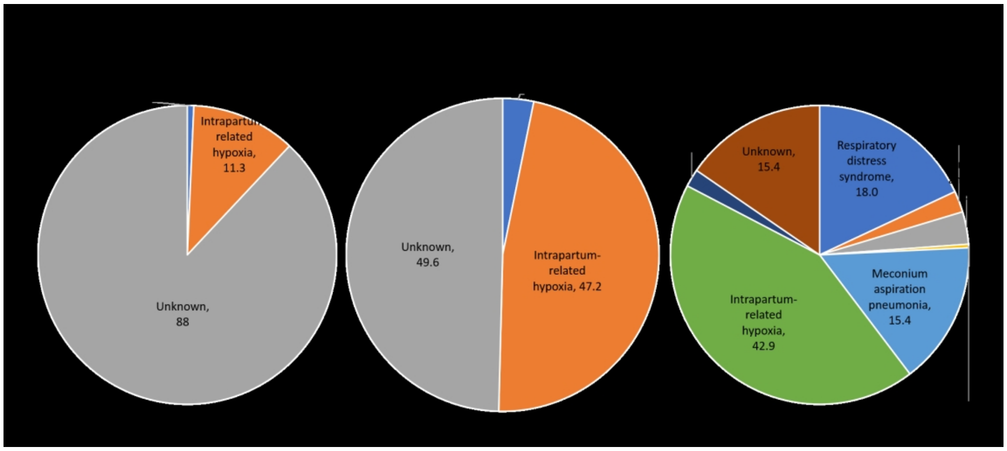

Figure 1: Cause of death for macerated stillbirths (n=142), fresh stillbirths (n=125) and early neonatal deaths (n=261)

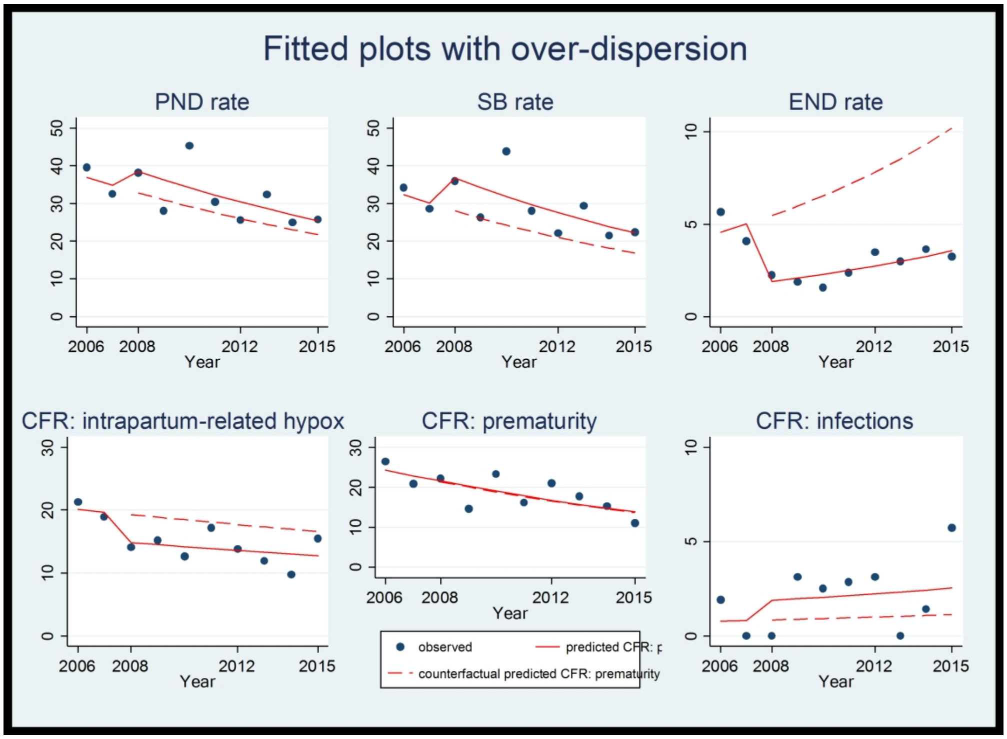

Figure 2: Interrupted time series with overdispersion

Supplementary Materials

Supplementary Table 1: Stillbirth rate (per 1000 births), early neonatal mortality rate (per 1000 live births) and perinatal mortality rate (per 1000 births).

Year	Total Births	Live Births	Stillbirth	Stillbirth Rate	Early NND	Early NND Rate	Perinatal Mortality	Perinatal Mortality Rate
2006	6468	6020	213	32.9	96	16	309	47.8
2007	7290	6881	210	28.8	193	28.1	403	55.3
2008	8386	8408	291	34.7	145	17.3	436	52.0
2009	8452	7995	207	24.5	114	14.3	321	38.0
2010	9513	8877	229	24.1	132	14.9	362	38.0
2011	8050	7610	220	27.3	166	21.8	386	47.9
2012	6732	6593	149	22.1	167	25.4	316	47.0
2013	6376	6011	183	28.7	117	19.4	300	47.0
2014	6026	5764	136	22.6	110	19	246	40.8
2015	5462	5222	120	22	112	21.5	232	42.6

For peer review only

Supplementary Table 2: **Cause of death for stillbirth and early neonatal death.**

Cause of Death	Macerated Stillbirth n=142 (%)	Fresh Stillbirth n=124 (%)	Early Neonatal Death n=259 (%)	TOTAL n=526 (%)
Respiratory distress syndrome	-	-	47 (18.0)	**47 (8.9)**
Infections / Septicaemia	1 (0.7)	0	6 (2.3)	**7 (1.3)**
Congenital anomaly	0	4 (3.2)	9 (3.5)	**13 (2.5)**
Meningitis	-	-	1 (0.4)	**1 (0.2)**
Meconium aspiration pneumonia	-	-	40 (15.4)	**40 (7.6)**
Intrapartum-related hypoxia	16 (11.3)	59 (47.6)	111 (42.9)	**186 (35.4)**
Haemorrhagic disease of newborn	-	-	5 (1.9)	**5 (1.0)**
Unknown	125 (88.0)	61 (49.6)	40 (15.4)	**227 (43.2)**

For peer review only

Supplementary Table 3: Case fatality rates (CFR)

Cause of death		2006	2007	2008	2009	2010	2011	2012	2013	2014	2015
**Intrapartum-related hypoxia**	Intrapartum-related hypoxia admissions	202	291	334	302	316	297	247	252	195	187
	Intrapartum-related hypoxia deaths	43	55	47	46	40	51	34	30	19	29
	**Intrapartum-related hypoxia CFR (%)**	**21.3**	**18.9**	**14.1**	**15.2**	**12.7**	**17.2**	**13.8**	**11.9**	**9.7**	**15.5**
**Prematurity**	Prematurity admissions	163	168	194	246	210	328	286	304	336	371
	Premature deaths	43	35	43	36	49	53	60	54	51	41
	**Prematurity CFR (%)**	**26.4**	**20.8**	**22.2**	**14.6**	**23.3**	**16.2**	**21.0**	**17.8**	**15.2**	**11.1**
**Infections**	Number of admissions for infections	104	149	82	128	120	140	129	171	141	87
	Deaths due to infections	2	0	0	4	3	4	4	0	2	5
	**Infections CFR (%)**	**1.9**	**0**	**0**	**3.1**	**2.5**	**2.9**	**3.1**	**0.0**	**1.4**	**5.7**

For peer review only

Supplementary Table 4: Standard of care

Standard of care	MSB (%)	FSB (%)	ENND (%)	Total (%)
Optimal	82 (67.8)	58 (49.2)	112 (45.7)	262 (53.0)
Probably acceptable	21 (17.4)	28 (23.7)	58 (23.7)	107 (21.7)
Probably sub-optimal	12 (9.9)	24 (20.3)	46 (18.8)	82 (16.6)
Sub-optimal	6 (5.0)	8 (6.8)	29 (11.8)	43 (8.7)
**Total**	**121 (100)**	**118 (100)**	**245 (100)**	**494 (100)**

For peer review only
